# Supplementary material for: The Citizen Phage Library: Rapid Isolation of Phages for the Treatment of Antibiotic Resistant Infections in the UK
Source: Microorganisms. 2024 Jan 25;12(2):253. doi: 10.3390/microorganisms12020253 (PMC10893117; doi:10.3390/microorganisms12020253)
Supplement: Supplementary file 1 [file microorganisms-2789885 Supplementary Material_02_Figure S6.pdf › microorganisms-2789885 Supplementary Material_02_Figure S6.pdf]

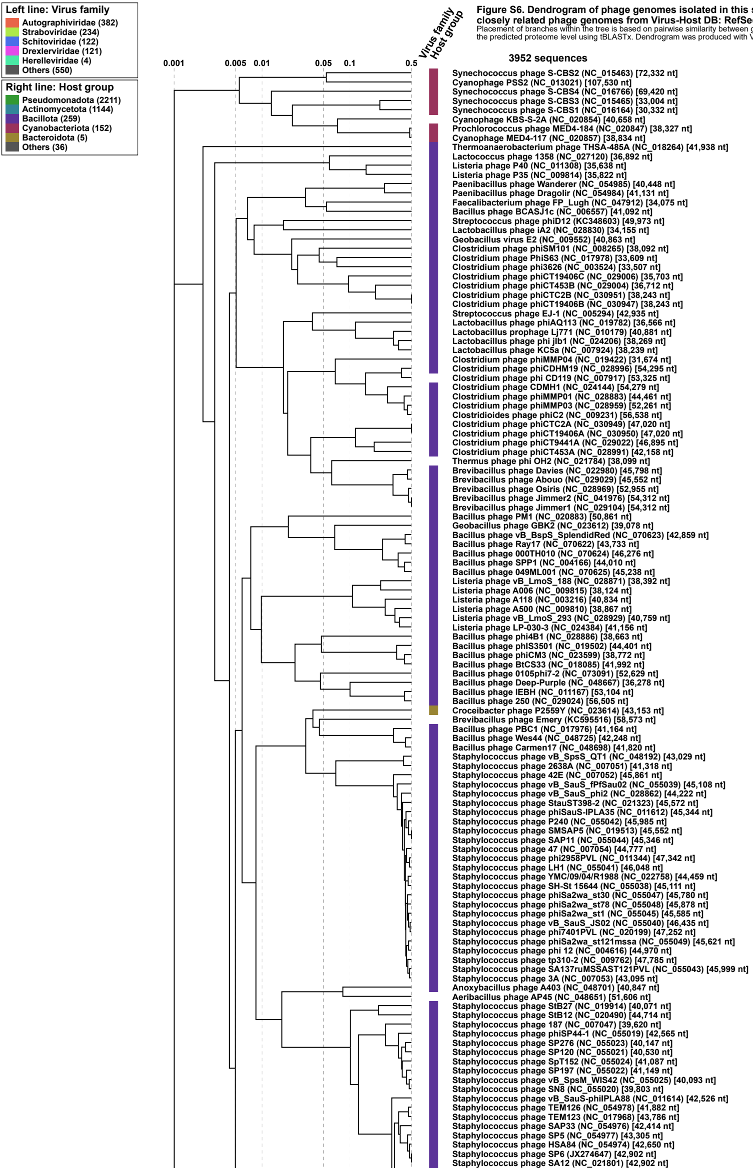

Figure S6. Dendrogram of phage genomes isolated in this study, with 3952 closely related phage genomes from Virus-Host DB: RefSeq release 220.
Placement of branches within the tree is based on pairwise similarity between genomes, calculated at the predicted proteome level using tBLASTx. Dendrogram was produced with VipTree server v. 4.0.

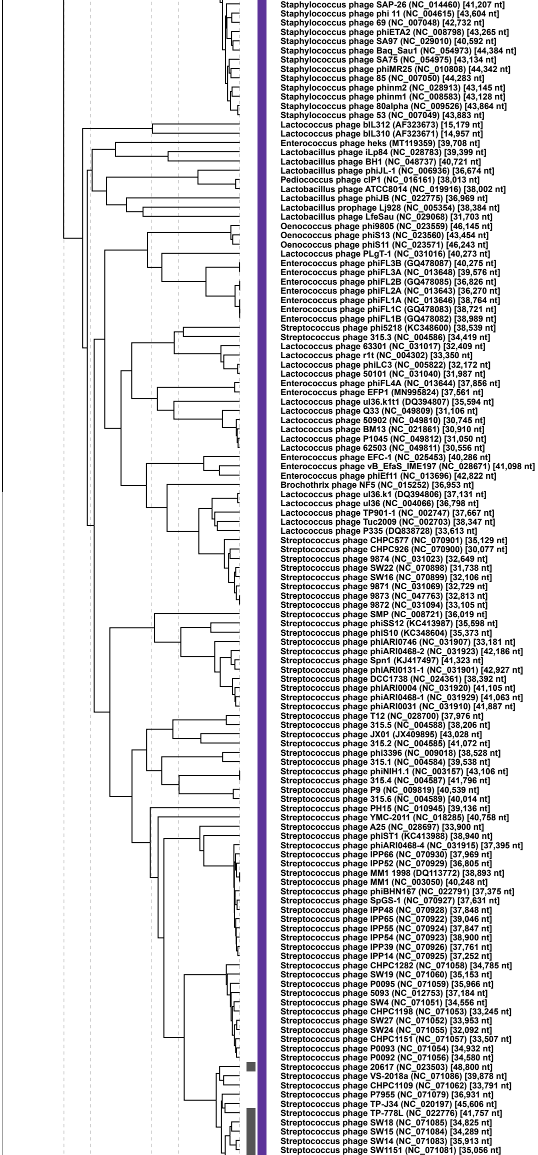

Staphylococcus phage SAP-26 (NC_014460) [41,207 nt]
Staphylococcus phage phi 11 (NC_004615) [43,604 nt]
Staphylococcus phage 69 (NC_007048) [42,732 nt]
Staphylococcus phage phiETA2 (NC_008798) [43,265 nt]
Staphylococcus phage SA97 (NC_029010) [40,592 nt]
Staphylococcus phage Baq_Sau1 (NC_054973) [44,384 nt]
Staphylococcus phage SA75 (NC_054975) [43,134 nt]
Staphylococcus phage phiMR25 (NC_010808) [44,342 nt]
Staphylococcus phage 85 (NC_007050) [44,283 nt]
Staphylococcus phage phinm2 (NC_028913) [43,145 nt]
Staphylococcus phage phinm1 (NC_008583) [43,128 nt]
Staphylococcus phage 80alpha (NC_009526) [43,864 nt]
Staphylococcus phage 53 (NC_007049) [43,883 nt]
Lactococcus phage bIL312 (AF323673) [15,179 nt]
Lactococcus phage bIL310 (AF323671) [14,957 nt]
Enterococcus phage heks (MT119359) [39,708 nt]
Lactobacillus phage iLp84 (NC_028783) [39,399 nt]
Lactobacillus phage BH1 (NC_048737) [40,721 nt]
Lactobacillus phage phiJL-1 (NC_006936) [36,674 nt]
Pediococcus phage clP1 (NC_016161) [38,013 nt]
Lactobacillus phage ATCC8014 (NC_019916) [38,002 nt]
Lactobacillus phage phiJB (NC_022775) [36,969 nt]
Lactobacillus prophage Lj928 (NC_005354) [38,384 nt]
Lactobacillus phage LfeSau (NC_029068) [31,703 nt]
Oenococcus phage phi9805 (NC_023559) [46,145 nt]
Oenococcus phage phiS13 (NC_023560) [43,454 nt]
Oenococcus phage phiS11 (NC_023571) [46,243 nt]
Lactococcus phage PLgT-1 (NC_031016) [40,273 nt]
Enterococcus phage phiFL3B (GQ478087) [40,275 nt]
Enterococcus phage phiFL3A (NC_013648) [39,576 nt]
Enterococcus phage phiFL2B (GQ478085) [36,826 nt]
Enterococcus phage phiFL2A (NC_013643) [36,270 nt]
Enterococcus phage phiFL1A (NC_013646) [38,764 nt]
Enterococcus phage phiFL1C (GQ478083) [38,721 nt]
Enterococcus phage phiFL1B (GQ478082) [38,989 nt]
Streptococcus phage phi5218 (KC348600) [38,539 nt]
Streptococcus phage 315.3 (NC_004586) [34,419 nt]
Lactococcus phage 63301 (NC_031017) [32,409 nt]
Lactococcus phage r1t (NC_004302) [33,350 nt]
Lactococcus phage phiLC3 (NC_005822) [32,172 nt]
Lactococcus phage 50101 (NC_031040) [31,987 nt]
Enterococcus phage phiFL4A (NC_013644) [37,856 nt]
Enterococcus phage EFP1 (MN995824) [37,561 nt]
Lactococcus phage ul36.k1t1 (DQ394807) [35,594 nt]
Lactococcus phage Q33 (NC_049809) [31,106 nt]
Lactococcus phage 50902 (NC_049810) [30,745 nt]
Lactococcus phage BM13 (NC_021861) [30,910 nt]
Lactococcus phage P1045 (NC_049812) [31,050 nt]
Lactococcus phage 62503 (NC_049811) [30,556 nt]
Enterococcus phage EFC-1 (NC_025453) [40,286 nt]
Enterococcus phage vB_EfaS_IME197 (NC_028671) [41,098 nt]
Enterococcus phage phiEf11 (NC_013696) [42,822 nt]
Brochothrix phage NF5 (NC_015252) [36,953 nt]
Lactococcus phage ul36.k1 (DQ394806) [37,131 nt]
Lactococcus phage ul36 (NC_004066) [36,798 nt]
Lactococcus phage TP901-1 (NC_002747) [37,667 nt]
Lactococcus phage Tuc2009 (NC_002703) [38,347 nt]
Lactococcus phage P335 (DQ838728) [33,613 nt]
Streptococcus phage CHPC577 (NC_070901) [35,129 nt]
Streptococcus phage CHPC926 (NC_070900) [30,077 nt]
Streptococcus phage 9874 (NC_031023) [32,649 nt]
Streptococcus phage SW22 (NC_070898) [31,738 nt]
Streptococcus phage SW16 (NC_070899) [32,106 nt]
Streptococcus phage 9871 (NC_031069) [32,729 nt]
Streptococcus phage 9873 (NC_047763) [32,813 nt]
Streptococcus phage 9872 (NC_031094) [33,105 nt]
Streptococcus phage SMP (NC_008721) [36,019 nt]
Streptococcus phage phiSS12 (KC413987) [35,598 nt]
Streptococcus phage phiS10 (KC348604) [35,373 nt]
Streptococcus phage phiARI0746 (NC_031907) [33,181 nt]
Streptococcus phage phiARI0468-2 (NC_031923) [42,186 nt]
Streptococcus phage Spn1 (KJ417497) [41,323 nt]
Streptococcus phage phiARI0131-1 (NC_031901) [42,927 nt]
Streptococcus phage DCC1738 (NC_024361) [38,392 nt]
Streptococcus phage phiARI0004 (NC_031920) [41,105 nt]
Streptococcus phage phiARI0468-1 (NC_031929) [41,063 nt]
Streptococcus phage phiARI0031 (NC_031910) [41,887 nt]
Streptococcus phage T12 (NC_028700) [37,976 nt]
Streptococcus phage 315.5 (NC_004588) [38,206 nt]
Streptococcus phage JX01 (JX409895) [43,028 nt]
Streptococcus phage 315.2 (NC_004585) [41,072 nt]
Streptococcus phage phi3396 (NC_009018) [38,528 nt]
Streptococcus phage 315.1 (NC_004584) [39,538 nt]
Streptococcus phage phiNIH1.1 (NC_003157) [43,106 nt]
Streptococcus phage 315.4 (NC_004587) [41,796 nt]
Streptococcus phage P9 (NC_009819) [40,539 nt]
Streptococcus phage 315.6 (NC_004589) [40,014 nt]
Streptococcus phage PH15 (NC_010945) [39,136 nt]
Streptococcus phage YMC-2011 (NC_018285) [40,758 nt]
Streptococcus phage A25 (NC_028697) [33,900 nt]
Streptococcus phage phiST1 (KC413988) [38,940 nt]
Streptococcus phage phiARI0468-4 (NC_031915) [37,395 nt]
Streptococcus phage IPP66 (NC_070930) [37,969 nt]
Streptococcus phage IPP52 (NC_070929) [36,805 nt]
Streptococcus phage MM1 1998 (DQ113772) [38,893 nt]
Streptococcus phage MM1 (NC_003050) [40,248 nt]
Streptococcus phage phiBHN167 (NC_022791) [37,375 nt]
Streptococcus phage SpGS-1 (NC_070927) [37,631 nt]
Streptococcus phage IPP48 (NC_070928) [37,848 nt]
Streptococcus phage IPP65 (NC_070922) [39,046 nt]
Streptococcus phage IPP55 (NC_070924) [37,847 nt]
Streptococcus phage IPP54 (NC_070923) [38,900 nt]
Streptococcus phage IPP39 (NC_070926) [37,761 nt]
Streptococcus phage IPP14 (NC_070925) [37,252 nt]
Streptococcus phage CHPC1282 (NC_071058) [34,785 nt]
Streptococcus phage SW19 (NC_071060) [35,153 nt]
Streptococcus phage P0095 (NC_071059) [35,966 nt]
Streptococcus phage 5093 (NC_012753) [37,184 nt]
Streptococcus phage SW4 (NC_071051) [34,556 nt]
Streptococcus phage CHPC1198 (NC_071053) [33,245 nt]
Streptococcus phage SW27 (NC_071052) [33,953 nt]
Streptococcus phage SW24 (NC_071055) [32,092 nt]
Streptococcus phage CHPC1151 (NC_071057) [33,507 nt]
Streptococcus phage P0093 (NC_071054) [34,932 nt]
Streptococcus phage P0092 (NC_071056) [34,580 nt]
Streptococcus phage 20617 (NC_023503) [48,800 nt]
Streptococcus phage VS-2018a (NC_071086) [39,878 nt]
Streptococcus phage CHPC1109 (NC_071062) [33,791 nt]
Streptococcus phage P7955 (NC_071079) [36,931 nt]
Streptococcus phage TP-J34 (NC_020197) [45,606 nt]
Streptococcus phage TP-778L (NC_022776) [41,757 nt]
Streptococcus phage SW18 (NC_071085) [34,825 nt]
Streptococcus phage SW15 (NC_071084) [34,289 nt]
Streptococcus phage SW14 (NC_071083) [35,913 nt]
Streptococcus phage SW1151 (NC_071081) [35,056 nt]

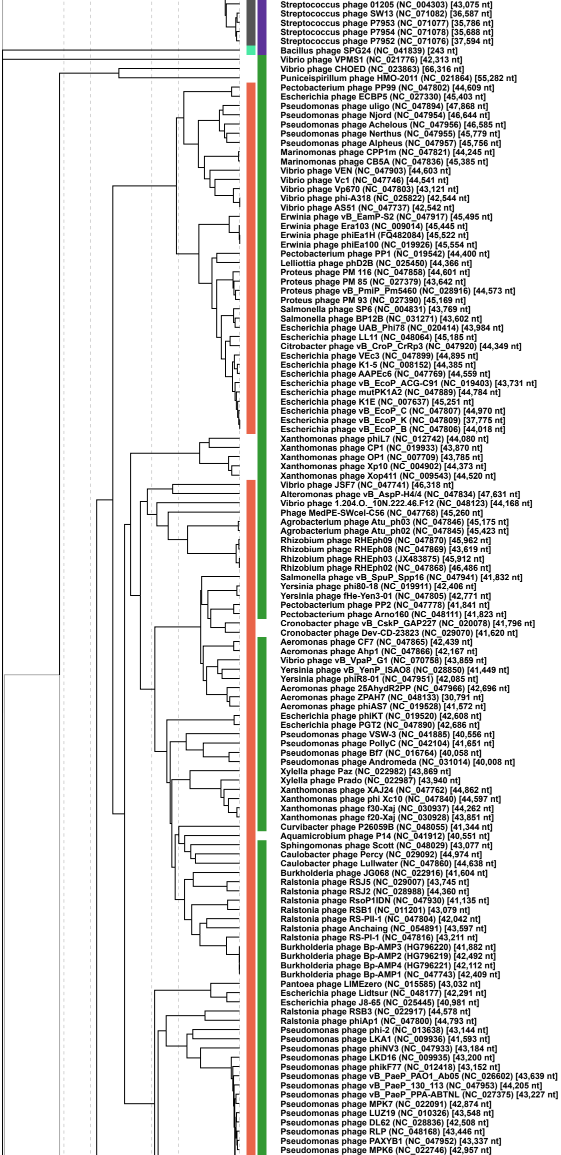

Streptococcus phage 01205 (NC_004303) [43,075 nt]
Streptococcus phage SW13 (NC_071082) [36,587 nt]
Streptococcus phage P7953 (NC_071077) [35,786 nt]
Streptococcus phage P7954 (NC_071078) [35,688 nt]
Streptococcus phage P7952 (NC_071076) [37,594 nt]
Bacillus phage SPG24 (NC_041839) [243 nt]
Vibrio phage VPMS1 (NC_021776) [42,313 nt]
Vibrio phage CHOED (NC_023863) [66,316 nt]
Puniceispirillum phage HMO-2011 (NC_021864) [55,282 nt]
Pectobacterium phage PP99 (NC_047802) [44,609 nt]
Escherichia phage ECBP5 (NC_027330) [45,403 nt]
Pseudomonas phage uligo (NC_047894) [47,868 nt]
Pseudomonas phage Njord (NC_047954) [46,644 nt]
Pseudomonas phage Achelous (NC_047956) [46,585 nt]
Pseudomonas phage Nerthus (NC_047955) [45,779 nt]
Pseudomonas phage Alpheus (NC_047957) [45,756 nt]
Marinomonas phage CPP1m (NC_047821) [44,245 nt]
Marinomonas phage CB5A (NC_047836) [45,385 nt]
Vibrio phage VEN (NC_047903) [44,603 nt]
Vibrio phage Vc1 (NC_047746) [44,541 nt]
Vibrio phage Vp670 (NC_047803) [43,121 nt]
Vibrio phage phi-A318 (NC_025822) [42,544 nt]
Vibrio phage AS51 (NC_047737) [42,542 nt]
Erwinia phage vB_EamP-S2 (NC_047917) [45,495 nt]
Erwinia phage Era103 (NC_009014) [45,445 nt]
Erwinia phage phiEa1H (FQ482084) [45,522 nt]
Erwinia phage phiEa100 (NC_019926) [45,554 nt]
Pectobacterium phage PP1 (NC_019542) [44,400 nt]
Lelliottia phage phD2B (NC_025450) [44,366 nt]
Proteus phage PM 116 (NC_047858) [44,601 nt]
Proteus phage PM 85 (NC_027379) [43,642 nt]
Proteus phage vB_PmiP_Pm5460 (NC_028916) [44,573 nt]
Proteus phage PM 93 (NC_027390) [45,169 nt]
Salmonella phage SP6 (NC_004831) [43,769 nt]
Salmonella phage BP12B (NC_031271) [43,602 nt]
Escherichia phage UAB_Phi78 (NC_020414) [43,984 nt]
Escherichia phage LL11 (NC_048064) [45,185 nt]
Citrobacter phage vB_CroP_CrRp3 (NC_047920) [44,349 nt]
Escherichia phage VEc3 (NC_047899) [44,895 nt]
Escherichia phage K1-5 (NC_008152) [44,385 nt]
Escherichia phage AAPEc6 (NC_047769) [44,559 nt]
Escherichia phage vB_EcoP_ACG-C91 (NC_019403) [43,731 nt]
Escherichia phage mutPK1A2 (NC_047889) [44,784 nt]
Escherichia phage K1E (NC_007637) [45,251 nt]
Escherichia phage vB_EcoP_C (NC_047807) [44,970 nt]
Escherichia phage vB_EcoP_K (NC_047809) [37,775 nt]
Escherichia phage vB_EcoP_B (NC_047806) [44,018 nt]
Xanthomonas phage phiL7 (NC_012742) [44,080 nt]
Xanthomonas phage CP1 (NC_019933) [43,870 nt]
Xanthomonas phage OP1 (NC_007709) [43,785 nt]
Xanthomonas phage Xp10 (NC_004902) [44,373 nt]
Xanthomonas phage Xop411 (NC_009543) [44,520 nt]
Vibrio phage JSF7 (NC_047741) [46,318 nt]
Alteromonas phage vB_AspP-H4/4 (NC_047834) [47,631 nt]
Vibrio phage 1.204.O._10N.222.46.F12 (NC_048123) [44,168 nt]
Phage MedPE-SWcel-C56 (NC_047768) [45,260 nt]
Agrobacterium phage Atu_ph03 (NC_047846) [45,175 nt]
Agrobacterium phage Atu_ph02 (NC_047845) [45,423 nt]
Rhizobium phage RHEph09 (NC_047870) [45,962 nt]
Rhizobium phage RHEph08 (NC_047869) [43,619 nt]
Rhizobium phage RHEph03 (JX483875) [45,912 nt]
Rhizobium phage RHEph02 (NC_047868) [46,486 nt]
Salmonella phage vB_SpuP_Spp16 (NC_047941) [41,832 nt]
Yersinia phage phi80-18 (NC_019911) [42,406 nt]
Yersinia phage fHe-Yen3-01 (NC_047805) [42,771 nt]
Pectobacterium phage PP2 (NC_047778) [41,841 nt]
Pectobacterium phage Arno160 (NC_048111) [41,823 nt]
Cronobacter phage vB_CskP_GAP227 (NC_020078) [41,796 nt]
Cronobacter phage Dev-CD-23823 (NC_029070) [41,620 nt]
Aeromonas phage CF7 (NC_047865) [42,439 nt]
Aeromonas phage Ahp1 (NC_047866) [42,167 nt]
Vibrio phage vB_VpaP_G1 (NC_070758) [43,859 nt]
Yersinia phage vB_YenP_ISAO8 (NC_028850) [41,449 nt]
Yersinia phage phiR8-01 (NC_047951) [42,085 nt]
Aeromonas phage 25AhydR2PP (NC_047966) [42,696 nt]
Aeromonas phage ZPAH7 (NC_048133) [30,791 nt]
Aeromonas phage phiAS7 (NC_019528) [41,572 nt]
Escherichia phage phiKT (NC_019520) [42,608 nt]
Escherichia phage PGT2 (NC_047890) [42,686 nt]
Pseudomonas phage VSW-3 (NC_041885) [40,556 nt]
Pseudomonas phage PollyC (NC_042104) [41,651 nt]
Pseudomonas phage Bf7 (NC_016764) [40,058 nt]
Pseudomonas phage Andromeda (NC_031014) [40,008 nt]
Xylella phage Paz (NC_022982) [43,869 nt]
Xylella phage Prado (NC_022987) [43,940 nt]
Xanthomonas phage XAJ24 (NC_047762) [44,862 nt]
Xanthomonas phage phi Xc10 (NC_047840) [44,597 nt]
Xanthomonas phage f30-Xaj (NC_030937) [44,262 nt]
Xanthomonas phage f20-Xaj (NC_030928) [43,851 nt]
Curvibacter phage P26059B (NC_048055) [41,344 nt]
Aquamicrobium phage P14 (NC_041912) [40,551 nt]
Sphingomonas phage Scott (NC_048029) [43,077 nt]
Caulobacter phage Percy (NC_029092) [44,974 nt]
Caulobacter phage Lullwater (NC_047860) [44,638 nt]
Burkholderia phage JG068 (NC_022916) [41,604 nt]
Ralstonia phage RSJ5 (NC_029007) [43,745 nt]
Ralstonia phage RSJ2 (NC_028988) [44,360 nt]
Ralstonia phage RsoP1IDN (NC_047930) [41,135 nt]
Ralstonia phage RSB1 (NC_011201) [43,079 nt]
Ralstonia phage RS-PII-1 (NC_047804) [42,042 nt]
Ralstonia phage Anchaing (NC_054891) [43,597 nt]
Ralstonia phage RS-PI-1 (NC_047816) [43,211 nt]
Burkholderia phage Bp-AMP3 (HG796220) [41,882 nt]
Burkholderia phage Bp-AMP2 (HG796219) [42,492 nt]
Burkholderia phage Bp-AMP4 (HG796221) [42,112 nt]
Burkholderia phage Bp-AMP1 (NC_047743) [42,409 nt]
Pantoea phage LIMEzero (NC_015585) [43,032 nt]
Escherichia phage Lidtsur (NC_048177) [42,291 nt]
Escherichia phage J8-65 (NC_025445) [40,981 nt]
Ralstonia phage RSB3 (NC_022917) [44,578 nt]
Ralstonia phage phiAp1 (NC_047800) [44,793 nt]
Pseudomonas phage phi-2 (NC_013638) [43,144 nt]
Pseudomonas phage LKA1 (NC_009936) [41,593 nt]
Pseudomonas phage phiNV3 (NC_047933) [43,184 nt]
Pseudomonas phage LKD16 (NC_009935) [43,200 nt]
Pseudomonas phage phikF77 (NC_012418) [43,152 nt]
Pseudomonas phage vB_PaeP_PAO1_Ab05 (NC_026602) [43,639 nt]
Pseudomonas phage vB_PaeP_130_113 (NC_047953) [44,205 nt]
Pseudomonas phage vB_PaeP_PPA-ABTNL (NC_027375) [43,227 nt]
Pseudomonas phage MPK7 (NC_022091) [42,874 nt]
Pseudomonas phage LUZ19 (NC_010326) [43,548 nt]
Pseudomonas phage DL62 (NC_028836) [42,508 nt]
Pseudomonas phage RLP (NC_048168) [43,446 nt]
Pseudomonas phage PAXYB1 (NC_047952) [43,337 nt]
Pseudomonas phage MPK6 (NC_022746) [42,957 nt]

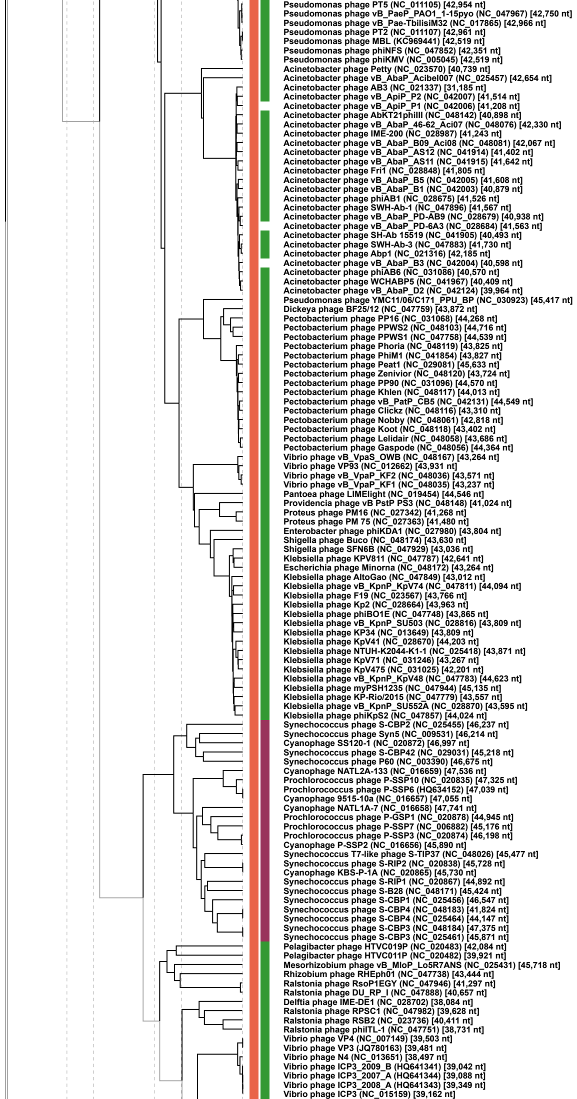
Pseudomonas phage PT5 (NC_011105) [42,954 nt]
Pseudomonas phage vB_PaeP_PAO1_1-15pyo (NC_047967) [42,750 nt]
Pseudomonas phage vB_Pae-TbilisiM32 (NC_017865) [42,966 nt]
Pseudomonas phage PT2 (NC_011107) [42,961 nt]
Pseudomonas phage MBL (KC969441) [42,519 nt]
Pseudomonas phage phiNFS (NC_047852) [42,351 nt]
Pseudomonas phage phiKMV (NC_005045) [42,519 nt]
Acinetobacter phage Petty (NC_023570) [40,739 nt]
Acinetobacter phage vB_AbaP_Acibel007 (NC_025457) [42,654 nt]
Acinetobacter phage AB3 (NC_021337) [31,185 nt]
Acinetobacter phage vB_ApiP_P2 (NC_042007) [41,514 nt]
Acinetobacter phage vB_ApiP_P1 (NC_042006) [41,208 nt]
Acinetobacter phage AbKT21phiIII (NC_048142) [40,898 nt]
Acinetobacter phage vB_AbaP_46-62_Aci07 (NC_048076) [42,330 nt]
Acinetobacter phage IME-200 (NC_028987) [41,243 nt]
Acinetobacter phage vB_AbaP_B09_Aci08 (NC_048081) [42,067 nt]
Acinetobacter phage vB_AbaP_AS12 (NC_041914) [41,402 nt]
Acinetobacter phage vB_AbaP_AS11 (NC_041915) [41,642 nt]
Acinetobacter phage Fri1 (NC_028848) [41,805 nt]
Acinetobacter phage vB_AbaP_B5 (NC_042005) [41,608 nt]
Acinetobacter phage vB_AbaP_B1 (NC_042003) [40,879 nt]
Acinetobacter phage phiAB1 (NC_028675) [41,526 nt]
Acinetobacter phage SWH-Ab-1 (NC_047896) [41,567 nt]
Acinetobacter phage vB_AbaP_PD-AB9 (NC_028679) [40,938 nt]
Acinetobacter phage vB_AbaP_PD-6A3 (NC_028684) [41,563 nt]
Acinetobacter phage SH-Ab 15519 (NC_041905) [40,493 nt]
Acinetobacter phage SWH-Ab-3 (NC_047883) [41,730 nt]
Acinetobacter phage Abp1 (NC_021316) [42,185 nt]
Acinetobacter phage vB_AbaP_B3 (NC_042004) [40,598 nt]
Acinetobacter phage phiAB6 (NC_031086) [40,570 nt]
Acinetobacter phage WCHABP5 (NC_041967) [40,409 nt]
Acinetobacter phage vB_AbaP_D2 (NC_042124) [39,964 nt]
Pseudomonas phage YMC11/06/C171_PPU_BP (NC_030923) [45,417 nt]
Dickeya phage BF25/12 (NC_047759) [43,872 nt]
Pectobacterium phage PP16 (NC_031068) [44,268 nt]
Pectobacterium phage PPWS2 (NC_048103) [44,716 nt]
Pectobacterium phage PPWS1 (NC_047758) [44,539 nt]
Pectobacterium phage Phoria (NC_048119) [43,825 nt]
Pectobacterium phage PhiM1 (NC_041854) [43,827 nt]
Pectobacterium phage Peat1 (NC_029081) [45,633 nt]
Pectobacterium phage Zenivior (NC_048120) [43,724 nt]
Pectobacterium phage PP90 (NC_031096) [44,570 nt]
Pectobacterium phage Khlen (NC_048117) [44,013 nt]
Pectobacterium phage vB_PatP_CB5 (NC_042131) [44,549 nt]
Pectobacterium phage Clickz (NC_048116) [43,310 nt]
Pectobacterium phage Nobby (NC_048061) [42,818 nt]
Pectobacterium phage Koot (NC_048118) [43,402 nt]
Pectobacterium phage Lelidair (NC_048058) [43,686 nt]
Pectobacterium phage Gaspode (NC_048056) [44,364 nt]
Vibrio phage vB_VpaS_OWB (NC_048167) [43,264 nt]
Vibrio phage VP93 (NC_012662) [43,931 nt]
Vibrio phage vB_VpaP_KF2 (NC_048036) [43,571 nt]
Vibrio phage vB_VpaP_KF1 (NC_048035) [43,237 nt]
Pantoea phage LIMElight (NC_019454) [44,546 nt]
Providencia phage vB PstP PS3 (NC_048148) [41,024 nt]
Proteus phage PM16 (NC_027342) [41,268 nt]
Proteus phage PM 75 (NC_027363) [41,480 nt]
Enterobacter phage phiKDA1 (NC_027980) [43,804 nt]
Shigella phage Buco (NC_048174) [43,630 nt]
Shigella phage SFN6B (NC_047929) [43,036 nt]
Klebsiella phage KPV811 (NC_047787) [42,641 nt]
Escherichia phage Minorna (NC_048172) [43,264 nt]
Klebsiella phage AltoGao (NC_047849) [43,012 nt]
Klebsiella phage vB_KpnP_KpV74 (NC_047811) [44,094 nt]
Klebsiella phage F19 (NC_023567) [43,766 nt]
Klebsiella phage Kp2 (NC_028664) [43,963 nt]
Klebsiella phage phiBO1E (NC_047748) [43,865 nt]
Klebsiella phage vB_KpnP_SU503 (NC_028816) [43,809 nt]
Klebsiella phage KP34 (NC_013649) [43,809 nt]
Klebsiella phage KpV41 (NC_028670) [44,203 nt]
Klebsiella phage NTUH-K2044-K1-1 (NC_025418) [43,871 nt]
Klebsiella phage KpV71 (NC_031246) [43,267 nt]
Klebsiella phage KpV475 (NC_031025) [42,201 nt]
Klebsiella phage vB_KpnP_KpV48 (NC_047783) [44,623 nt]
Klebsiella phage myPSH1235 (NC_047944) [45,135 nt]
Klebsiella phage KP-Rio/2015 (NC_047779) [43,557 nt]
Klebsiella phage vB_KpnP_SU552A (NC_028870) [43,595 nt]
Klebsiella phage phiKpS2 (NC_047857) [44,024 nt]
Synechococcus phage S-CBP2 (NC_025455) [46,237 nt]
Synechococcus phage Syn5 (NC_009531) [46,214 nt]
Cyanophage SS120-1 (NC_020872) [46,997 nt]
Synechococcus phage S-CBP42 (NC_029031) [45,218 nt]
Synechococcus phage P60 (NC_003390) [46,675 nt]
Cyanophage NATL2A-133 (NC_016659) [47,536 nt]
Prochlorococcus phage P-SSP10 (NC_020835) [47,325 nt]
Prochlorococcus phage P-SSP6 (HQ634152) [47,039 nt]
Cyanophage 9515-10a (NC_016657) [47,055 nt]
Cyanophage NATL1A-7 (NC_016658) [47,741 nt]
Prochlorococcus phage P-GSP1 (NC_020878) [44,945 nt]
Prochlorococcus phage P-SSP7 (NC_006882) [45,176 nt]
Prochlorococcus phage P-SSP3 (NC_020874) [46,198 nt]
Cyanophage P-SSP2 (NC_016656) [45,890 nt]
Synechococcus T7-like phage S-TIP37 (NC_048026) [45,477 nt]
Synechococcus phage S-RIP2 (NC_020838) [45,728 nt]
Cyanophage KBS-P-1A (NC_020865) [45,730 nt]
Synechococcus phage S-RIP1 (NC_020867) [44,892 nt]
Synechococcus phage S-B28 (NC_048171) [45,424 nt]
Synechococcus phage S-CBP1 (NC_025456) [46,547 nt]
Synechococcus phage S-CBP4 (NC_048183) [41,824 nt]
Synechococcus phage S-CBP4 (NC_025464) [44,147 nt]
Synechococcus phage S-CBP3 (NC_048184) [47,375 nt]
Synechococcus phage S-CBP3 (NC_025461) [45,871 nt]
Pelagibacter phage HTVC019P (NC_020483) [42,084 nt]
Pelagibacter phage HTVC011P (NC_020482) [39,921 nt]
Mesorhizobium phage vB_MloP_Lo5R7ANS (NC_025431) [45,718 nt]
Rhizobium phage RHEph01 (NC_047738) [43,444 nt]
Ralstonia phage RsoP1EGY (NC_047946) [41,297 nt]
Ralstonia phage DU_RP_I (NC_047888) [40,657 nt]
Delftia phage IME-DE1 (NC_028702) [38,084 nt]
Ralstonia phage RPSC1 (NC_047982) [39,628 nt]
Ralstonia phage RSB2 (NC_023736) [40,411 nt]
Ralstonia phage phiITL-1 (NC_047751) [38,731 nt]
Vibrio phage VP4 (NC_007149) [39,503 nt]
Vibrio phage VP3 (JQ780163) [39,481 nt]
Vibrio phage N4 (NC_013651) [38,497 nt]
Vibrio phage ICP3_2009_B (HQ641341) [39,042 nt]
Vibrio phage ICP3_2007_A (HQ641344) [39,088 nt]
Vibrio phage ICP3_2008_A (HQ641343) [39,349 nt]
Vibrio phage ICP3 (NC_015159) [39,162 nt]

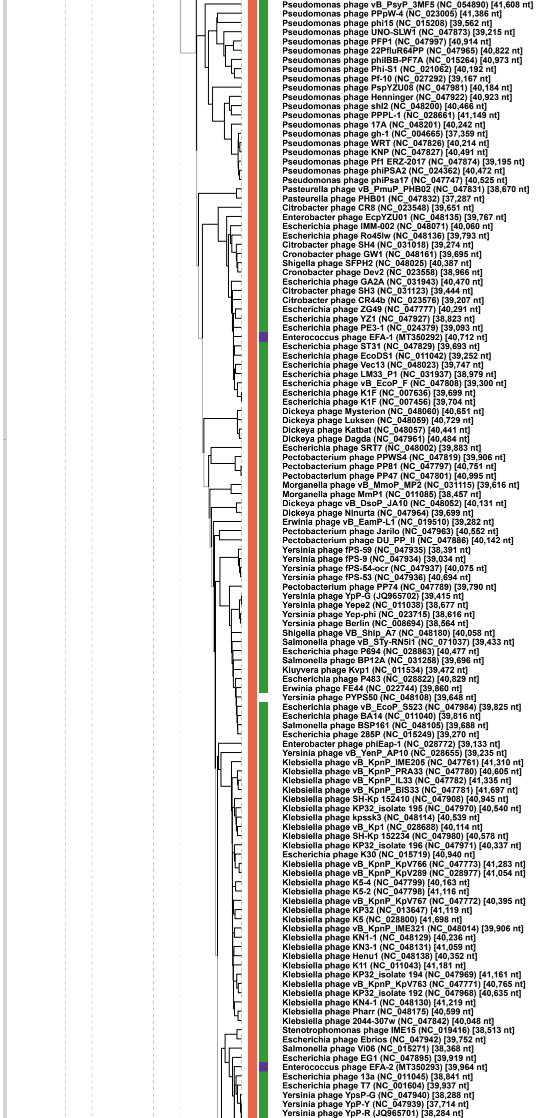
Pseudomonas phage vB_PsyP_3MF5 (NC_054890) [41,608 nt]
Pseudomonas phage PPpW-4 (NC_023005) [41,386 nt]
Pseudomonas phage phi15 (NC_015208) [39,562 nt]
Pseudomonas phage UNO-SLW1 (NC_047873) [39,215 nt]
Pseudomonas phage PFP1 (NC_047997) [40,914 nt]
Pseudomonas phage 22PfluR64PP (NC_047965) [40,822 nt]
Pseudomonas phage philBB-PF7A (NC_015264) [40,973 nt]
Pseudomonas phage Phi-S1 (NC_021062) [40,192 nt]
Pseudomonas phage Pf-10 (NC_027292) [39,167 nt]
Pseudomonas phage PspYZU08 (NC_047981) [40,184 nt]
Pseudomonas phage Henninger (NC_047922) [40,923 nt]
Pseudomonas phage shl2 (NC_048200) [40,466 nt]
Pseudomonas phage PPPL-1 (NC_028661) [41,149 nt]
Pseudomonas phage 17A (NC_048201) [40,242 nt]
Pseudomonas phage gh-1 (NC_004665) [37,359 nt]
Pseudomonas phage WRT (NC_047826) [40,214 nt]
Pseudomonas phage KNP (NC_047827) [40,491 nt]
Pseudomonas phage Pf1 ERZ-2017 (NC_047874) [39,195 nt]
Pseudomonas phage phiPSA2 (NC_024362) [40,472 nt]
Pseudomonas phage phiPsa17 (NC_047747) [40,525 nt]
Pasteurella phage vB_PmuP_PHB02 (NC_047831) [38,670 nt]
Pasteurella phage PHB01 (NC_047832) [37,287 nt]
Citrobacter phage CR8 (NC_023548) [39,651 nt]
Enterobacter phage EcpYZU01 (NC_048135) [39,767 nt]
Escherichia phage IMM-002 (NC_048071) [40,060 nt]
Escherichia phage Ro45lw (NC_048136) [39,793 nt]
Citrobacter phage SH4 (NC_031018) [39,274 nt]
Cronobacter phage GW1 (NC_048161) [39,695 nt]
Shigella phage SFPH2 (NC_048025) [40,387 nt]
Cronobacter phage Dev2 (NC_023558) [38,966 nt]
Escherichia phage GA2A (NC_031943) [40,470 nt]
Citrobacter phage SH3 (NC_031123) [39,444 nt]
Citrobacter phage CR44b (NC_023576) [39,207 nt]
Escherichia phage ZG49 (NC_047777) [40,291 nt]
Escherichia phage YZ1 (NC_047927) [38,823 nt]
Escherichia phage PE3-1 (NC_024379) [39,093 nt]
Enterococcus phage EFA-1 (MT350292) [40,712 nt]
Escherichia phage ST31 (NC_047829) [39,693 nt]
Escherichia phage EcoDS1 (NC_011042) [39,252 nt]
Escherichia phage Vec13 (NC_048023) [39,747 nt]
Escherichia phage LM33_P1 (NC_031937) [38,979 nt]
Escherichia phage vB_EcoP_F (NC_047808) [39,300 nt]
Escherichia phage K1F (NC_007636) [39,699 nt]
Escherichia phage K1F (NC_007456) [39,704 nt]
Dickeya phage Mysterion (NC_048060) [40,651 nt]
Dickeya phage Luksen (NC_048059) [40,729 nt]
Dickeya phage Katbat (NC_048057) [40,441 nt]
Dickeya phage Dagda (NC_047961) [40,484 nt]
Escherichia phage SRT7 (NC_048002) [39,883 nt]
Pectobacterium phage PPWS4 (NC_047819) [39,906 nt]
Pectobacterium phage PP81 (NC_047797) [40,751 nt]
Pectobacterium phage PP47 (NC_047801) [40,995 nt]
Morganella phage vB_MmoP_MP2 (NC_031115) [39,616 nt]
Morganella phage MmP1 (NC_011085) [38,457 nt]
Dickeya phage vB_DsoP_JA10 (NC_048052) [40,131 nt]
Dickeya phage Ninurta (NC_047964) [39,699 nt]
Erwinia phage vB_EamP-L1 (NC_019510) [39,282 nt]
Pectobacterium phage Jarilo (NC_047963) [40,552 nt]
Pectobacterium phage DU_PP_II (NC_047886) [40,142 nt]
Yersinia phage fPS-59 (NC_047935) [38,391 nt]
Yersinia phage fPS-9 (NC_047934) [39,034 nt]
Yersinia phage fPS-54-ocr (NC_047937) [40,075 nt]
Yersinia phage fPS-53 (NC_047936) [40,694 nt]
Pectobacterium phage PP74 (NC_047789) [39,790 nt]
Yersinia phage YpP-G (JQ965702) [39,415 nt]
Yersinia phage Yepe2 (NC_011038) [38,677 nt]
Yersinia phage Yep-phi (NC_023715) [38,616 nt]
Yersinia phage Berlin (NC_008694) [38,564 nt]
Shigella phage VB_Ship_A7 (NC_048180) [40,058 nt]
Salmonella phage vB_STy-RN5i1 (NC_071037) [39,433 nt]
Escherichia phage P694 (NC_028863) [40,477 nt]
Salmonella phage BP12A (NC_031258) [39,696 nt]
Kluyvera phage Kvp1 (NC_011534) [39,472 nt]
Escherichia phage P483 (NC_028822) [40,829 nt]
Erwinia phage FE44 (NC_022744) [39,860 nt]
Yersinia phage PYPS50 (NC_048108) [39,648 nt]
Escherichia phage vB_EcoP_S523 (NC_047984) [39,825 nt]
Escherichia phage BA14 (NC_011040) [39,816 nt]
Salmonella phage BSP161 (NC_048105) [39,688 nt]
Escherichia phage 285P (NC_015249) [39,270 nt]
Enterobacter phage phiEap-1 (NC_028772) [39,133 nt]
Yersinia phage vB_YenP_AP10 (NC_028655) [39,235 nt]
Klebsiella phage vB_KpnP_IME205 (NC_047761) [41,310 nt]
Klebsiella phage vB_KpnP_PRA33 (NC_047780) [40,605 nt]
Klebsiella phage vB_KpnP_IL33 (NC_047782) [41,335 nt]
Klebsiella phage vB_KpnP_BIS33 (NC_047781) [41,697 nt]
Klebsiella phage SH-Kp 152410 (NC_047908) [40,945 nt]
Klebsiella phage KP32_isolate 195 (NC_047970) [40,540 nt]
Klebsiella phage kpssk3 (NC_048114) [40,539 nt]
Klebsiella phage vB_Kp1 (NC_028688) [40,114 nt]
Klebsiella phage SH-Kp 152234 (NC_047980) [40,578 nt]
Klebsiella phage KP32_isolate 196 (NC_047971) [40,337 nt]
Escherichia phage K30 (NC_015719) [40,940 nt]
Klebsiella phage vB_KpnP_KpV766 (NC_047773) [41,283 nt]
Klebsiella phage vB_KpnP_KpV289 (NC_028977) [41,054 nt]
Klebsiella phage K5-4 (NC_047799) [40,163 nt]
Klebsiella phage K5-2 (NC_047798) [41,116 nt]
Klebsiella phage vB_KpnP_KpV767 (NC_047772) [40,395 nt]
Klebsiella phage KP32 (NC_013647) [41,119 nt]
Klebsiella phage K5 (NC_028800) [41,698 nt]
Klebsiella phage vB_KpnP_IME321 (NC_048014) [39,906 nt]
Klebsiella phage KN1-1 (NC_048129) [40,236 nt]
Klebsiella phage KN3-1 (NC_048131) [41,059 nt]
Klebsiella phage Henu1 (NC_048138) [40,352 nt]
Klebsiella phage K11 (NC_011043) [41,181 nt]
Klebsiella phage KP32_isolate 194 (NC_047969) [41,161 nt]
Klebsiella phage vB_KpnP_KpV763 (NC_047771) [40,765 nt]
Klebsiella phage KP32_isolate 192 (NC_047968) [40,635 nt]
Klebsiella phage KN4-1 (NC_048130) [41,219 nt]
Klebsiella phage Pharr (NC_048175) [40,599 nt]
Klebsiella phage 2044-307w (NC_047842) [40,048 nt]
Stenotrophomonas phage IME15 (NC_019416) [38,513 nt]
Escherichia phage Ebrios (NC_047942) [39,752 nt]
Salmonella phage Vi06 (NC_015271) [38,368 nt]
Escherichia phage EG1 (NC_047895) [39,919 nt]
Enterococcus phage EFA-2 (MT350293) [39,964 nt]
Escherichia phage 13a (NC_011045) [38,841 nt]
Escherichia phage T7 (NC_001604) [39,937 nt]
Yersinia phage YpsP-G (NC_047940) [38,288 nt]
Yersinia phage YpP-Y (NC_047939) [37,714 nt]
Yersinia phage YpP-R (JQ965701) [38,284 nt]

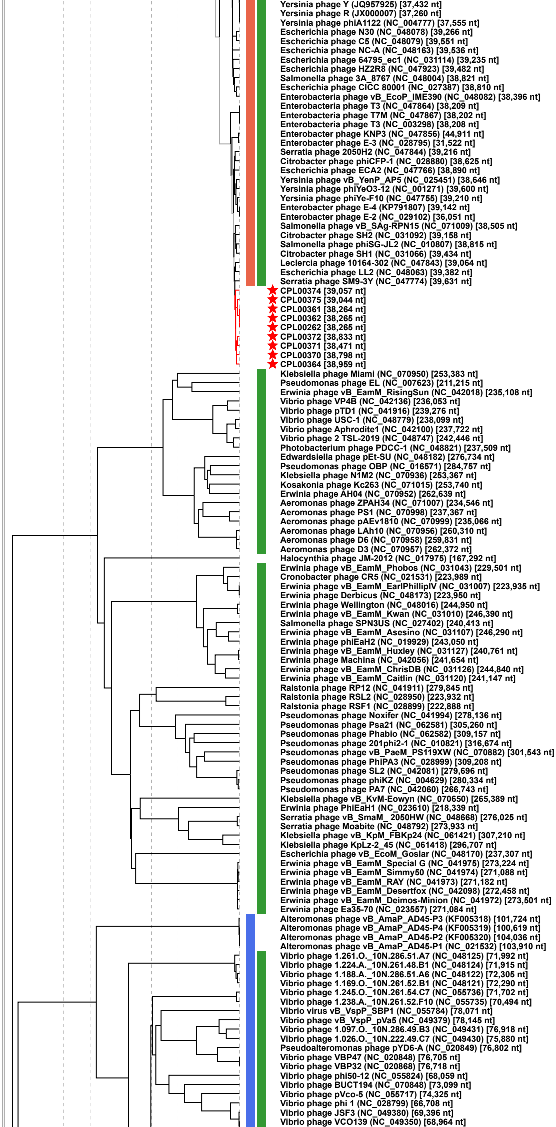
Yersinia phage Y (JQ957925) [37,432 nt]
Yersinia phage R (JX000007) [37,260 nt]
Yersinia phage phiA1122 (NC_004777) [37,555 nt]
Escherichia phage N30 (NC_048078) [39,266 nt]
Escherichia phage C5 (NC_048079) [39,551 nt]
Escherichia phage NC-A (NC_048163) [39,536 nt]
Escherichia phage 64795_ec1 (NC_031114) [39,235 nt]
Escherichia phage HZ2R8 (NC_047923) [39,482 nt]
Salmonella phage 3A_8767 (NC_048004) [38,821 nt]
Escherichia phage CICC 80001 (NC_027387) [38,810 nt]
Enterobacteria phage vB_EcoP_IME390 (NC_048082) [38,396 nt]
Enterobacteria phage T3 (NC_047864) [38,209 nt]
Enterobacteria phage T7M (NC_047867) [38,202 nt]
Enterobacteria phage T3 (NC_003298) [38,208 nt]
Enterobacter phage KNP3 (NC_047856) [44,911 nt]
Enterobacter phage E-3 (NC_028795) [31,522 nt]
Serratia phage 2050H2 (NC_047844) [39,216 nt]
Citrobacter phage phiCFP-1 (NC_028880) [38,625 nt]
Escherichia phage ECA2 (NC_047766) [38,890 nt]
Yersinia phage vB_YenP_AP5 (NC_025451) [38,646 nt]
Yersinia phage phiYeO3-12 (NC_001271) [39,600 nt]
Yersinia phage phiYe-F10 (NC_047755) [39,210 nt]
Enterobacter phage E-4 (KP791807) [39,142 nt]
Enterobacter phage E-2 (NC_029102) [36,051 nt]
Salmonella phage vB_SAg-RPN15 (NC_071009) [38,505 nt]
Citrobacter phage SH2 (NC_031092) [39,158 nt]
Salmonella phage phiSG-JL2 (NC_010807) [38,815 nt]
Citrobacter phage SH1 (NC_031066) [39,434 nt]
Leclercia phage 10164-302 (NC_047843) [39,064 nt]
Escherichia phage LL2 (NC_048063) [39,382 nt]
Serratia phage SM9-3Y (NC_047774) [39,631 nt]
CPL00374 [39,057 nt]
CPL00375 [39,044 nt]
CPL00361 [38,264 nt]
CPL00362 [38,265 nt]
CPL00262 [38,265 nt]
CPL00372 [38,833 nt]
CPL00371 [38,471 nt]
CPL00370 [38,798 nt]
CPL00364 [38,959 nt]
Klebsiella phage Miami (NC_070950) [253,383 nt]
Pseudomonas phage EL (NC_007623) [211,215 nt]
Erwinia phage vB_EamM_RisingSun (NC_042018) [235,108 nt]
Vibrio phage VP4B (NC_042136) [236,053 nt]
Vibrio phage pTD1 (NC_041916) [239,276 nt]
Vibrio phage USC-1 (NC_048779) [238,099 nt]
Vibrio phage Aphrodite1 (NC_042100) [237,722 nt]
Vibrio phage 2 TSL-2019 (NC_048747) [242,446 nt]
Photobacterium phage PDCC-1 (NC_048821) [237,509 nt]
Edwardsiella phage pEt-SU (NC_048182) [276,734 nt]
Pseudomonas phage OBP (NC_016571) [284,757 nt]
Klebsiella phage N1M2 (NC_070936) [253,367 nt]
Kosakonia phage Kc263 (NC_071015) [253,740 nt]
Erwinia phage AH04 (NC_070952) [262,639 nt]
Aeromonas phage ZPAH34 (NC_071007) [234,546 nt]
Aeromonas phage PS1 (NC_070998) [237,367 nt]
Aeromonas phage pAEv1810 (NC_070999) [235,066 nt]
Aeromonas phage LAh10 (NC_070956) [260,310 nt]
Aeromonas phage D6 (NC_070958) [259,831 nt]
Aeromonas phage D3 (NC_070957) [262,372 nt]
Halocynthia phage JM-2012 (NC_017975) [167,292 nt]
Erwinia phage vB_EamM_Phobos (NC_031043) [229,501 nt]
Cronobacter phage CR5 (NC_021531) [223,989 nt]
Erwinia phage vB_EamM_EarlPhillipIV (NC_031007) [223,935 nt]
Erwinia phage Derbicus (NC_048173) [223,950 nt]
Erwinia phage Wellington (NC_048016) [244,950 nt]
Erwinia phage vB_EamM_Kwan (NC_031010) [246,390 nt]
Salmonella phage SPN3US (NC_027402) [240,413 nt]
Erwinia phage vB_EamM_Asesino (NC_031107) [246,290 nt]
Erwinia phage phiEaH2 (NC_019929) [243,050 nt]
Erwinia phage vB_EamM_Huxley (NC_031127) [240,761 nt]
Erwinia phage Machina (NC_042056) [241,654 nt]
Erwinia phage vB_EamM_ChrisDB (NC_031126) [244,840 nt]
Erwinia phage vB_EamM_Caitlin (NC_031120) [241,147 nt]
Ralstonia phage RP12 (NC_041911) [279,845 nt]
Ralstonia phage RSL2 (NC_028950) [223,932 nt]
Ralstonia phage RSF1 (NC_028899) [222,888 nt]
Pseudomonas phage Noxifer (NC_041994) [278,136 nt]
Pseudomonas phage Psa21 (NC_062581) [305,260 nt]
Pseudomonas phage Phabio (NC_062582) [309,157 nt]
Pseudomonas phage 201phi2-1 (NC_010821) [316,674 nt]
Pseudomonas phage vB_PaeM_PS119XW (NC_070882) [301,543 nt]
Pseudomonas phage PhiPA3 (NC_028999) [309,208 nt]
Pseudomonas phage SL2 (NC_042081) [279,696 nt]
Pseudomonas phage phiKZ (NC_004629) [280,334 nt]
Pseudomonas phage PA7 (NC_042060) [266,743 nt]
Klebsiella phage vB_KvM-Eowyn (NC_070650) [265,389 nt]
Erwinia phage PhiEaH1 (NC_023610) [218,339 nt]
Serratia phage vB_SmaM_ 2050HW (NC_048668) [276,025 nt]
Serratia phage Moabite (NC_048792) [273,933 nt]
Klebsiella phage vB_KpM_FBKp24 (NC_061421) [307,210 nt]
Klebsiella phage KpLz-2_45 (NC_061418) [296,707 nt]
Escherichia phage vB_EcoM_Goslar (NC_048170) [237,307 nt]
Erwinia phage vB_EamM_Special G (NC_041975) [273,224 nt]
Erwinia phage vB_EamM_Simmy50 (NC_041974) [271,088 nt]
Erwinia phage vB_EamM_RAY (NC_041973) [271,182 nt]
Erwinia phage vB_EamM_Desertfox (NC_042098) [272,458 nt]
Erwinia phage vB_EamM_Deimos-Minion (NC_041972) [273,501 nt]
Erwinia phage Ea35-70 (NC_023557) [271,084 nt]
Alteromonas phage vB_AmaP_AD45-P3 (KF005318) [101,724 nt]
Alteromonas phage vB_AmaP_AD45-P4 (KF005319) [100,619 nt]
Alteromonas phage vB_AmaP_AD45-P2 (KF005320) [104,036 nt]
Alteromonas phage vB_AmaP_AD45-P1 (NC_021532) [103,910 nt]
Vibrio phage 1.261.O._10N.286.51.A7 (NC_048125) [71,992 nt]
Vibrio phage 1.224.A._10N.261.48.B1 (NC_048124) [71,915 nt]
Vibrio phage 1.188.A._10N.286.51.A6 (NC_048122) [72,305 nt]
Vibrio phage 1.169.O._10N.261.52.B1 (NC_048121) [72,290 nt]
Vibrio phage 1.245.O._10N.261.54.C7 (NC_055736) [71,702 nt]
Vibrio phage 1.238.A._10N.261.52.F10 (NC_055735) [70,494 nt]
Vibrio virus vB_VspP_SBP1 (NC_055784) [78,071 nt]
Vibrio phage vB_VspP_pVa5 (NC_049379) [78,145 nt]
Vibrio phage 1.097.O._10N.286.49.B3 (NC_049431) [76,918 nt]
Vibrio phage 1.026.O._10N.222.49.C7 (NC_049430) [75,880 nt]
Pseudoalteromonas phage pYD6-A (NC_020849) [76,802 nt]
Vibrio phage VBP47 (NC_020848) [76,705 nt]
Vibrio phage VBP32 (NC_020868) [76,718 nt]
Vibrio phage phi50-12 (NC_055824) [68,059 nt]
Vibrio phage BUCT194 (NC_070848) [73,099 nt]
Vibrio phage pVco-5 (NC_055717) [74,325 nt]
Vibrio phage phi 1 (NC_028799) [66,708 nt]
Vibrio phage JSF3 (NC_049380) [69,396 nt]
Vibrio phage VCO139 (NC_049350) [68,964 nt]

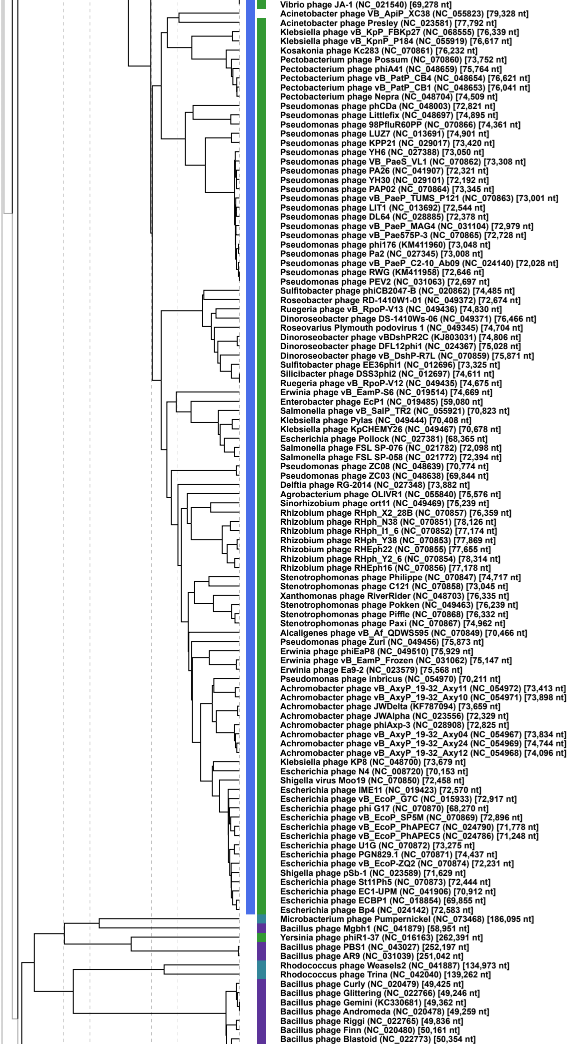
Vibrio phage JA-1 (NC_021540) [69,278 nt]
Acinetobacter phage VB_ApiP_XC38 (NC_055823) [79,328 nt]
Acinetobacter phage Presley (NC_023581) [77,792 nt]
Klebsiella phage vB_KpP_FBKp27 (NC_068555) [76,339 nt]
Klebsiella phage vB_KpnP_P184 (NC_055919) [76,617 nt]
Kosakonia phage Kc283 (NC_070861) [76,232 nt]
Pectobacterium phage Possum (NC_070860) [73,752 nt]
Pectobacterium phage phiA41 (NC_048659) [75,764 nt]
Pectobacterium phage vB_PatP_CB4 (NC_048654) [76,621 nt]
Pectobacterium phage vB_PatP_CB1 (NC_048653) [76,041 nt]
Pectobacterium phage Nepra (NC_048704) [74,509 nt]
Pseudomonas phage phCDa (NC_048003) [72,821 nt]
Pseudomonas phage Littlefix (NC_048697) [74,895 nt]
Pseudomonas phage 98PfluR60PP (NC_070866) [74,361 nt]
Pseudomonas phage LUZ7 (NC_013691) [74,901 nt]
Pseudomonas phage KPP21 (NC_029017) [73,420 nt]
Pseudomonas phage YH6 (NC_027388) [73,050 nt]
Pseudomonas phage VB_PaeS_VL1 (NC_070862) [73,308 nt]
Pseudomonas phage PA26 (NC_041907) [72,321 nt]
Pseudomonas phage YH30 (NC_029101) [72,192 nt]
Pseudomonas phage PAP02 (NC_070864) [73,345 nt]
Pseudomonas phage vB_PaeP_TUMS_P121 (NC_070863) [73,001 nt]
Pseudomonas phage LIT1 (NC_013692) [72,544 nt]
Pseudomonas phage DL64 (NC_028885) [72,378 nt]
Pseudomonas phage vB_PaeP_MAG4 (NC_031104) [72,979 nt]
Pseudomonas phage vB_Pae575P-3 (NC_070865) [72,728 nt]
Pseudomonas phage phi176 (KM411960) [73,048 nt]
Pseudomonas phage Pa2 (NC_027345) [73,008 nt]
Pseudomonas phage vB_PaeP_C2-10_Ab09 (NC_024140) [72,028 nt]
Pseudomonas phage RWG (KM411958) [72,646 nt]
Pseudomonas phage PEV2 (NC_031063) [72,697 nt]
Sulfitobacter phage phiCB2047-B (NC_020862) [74,485 nt]
Roseobacter phage RD-1410W1-01 (NC_049372) [72,674 nt]
Ruegeria phage vB_RpoP-V13 (NC_049436) [74,830 nt]
Dinoroseobacter phage DS-1410Ws-06 (NC_049371) [76,466 nt]
Roseovarius Plymouth podovirus 1 (NC_049345) [74,704 nt]
Dinoroseobacter phage vBDshPR2C (KJ803031) [74,806 nt]
Dinoroseobacter phage DFL12phi1 (NC_024367) [75,028 nt]
Dinoroseobacter phage vB_DshP-R7L (NC_070859) [75,871 nt]
Sulfitobacter phage EE36phi1 (NC_012696) [73,325 nt]
Silicibacter phage DSS3phi2 (NC_012697) [74,611 nt]
Ruegeria phage vB_RpoP-V12 (NC_049435) [74,675 nt]
Erwinia phage vB_EamP-S6 (NC_019514) [74,669 nt]
Enterobacter phage EcP1 (NC_019485) [59,080 nt]
Salmonella phage vB_SalP_TR2 (NC_055921) [70,823 nt]
Klebsiella phage Pylas (NC_049444) [70,408 nt]
Klebsiella phage KpCHEMY26 (NC_049467) [70,678 nt]
Escherichia phage Pollock (NC_027381) [68,365 nt]
Salmonella phage FSL SP-076 (NC_021782) [72,098 nt]
Salmonella phage FSL SP-058 (NC_021772) [72,394 nt]
Pseudomonas phage ZC08 (NC_048639) [70,774 nt]
Pseudomonas phage ZC03 (NC_048638) [69,844 nt]
Delftia phage RG-2014 (NC_027348) [73,882 nt]
Agrobacterium phage OLIVR1 (NC_055840) [75,576 nt]
Sinorhizobium phage ort11 (NC_049469) [75,239 nt]
Rhizobium phage RHph_X2_28B (NC_070857) [76,359 nt]
Rhizobium phage RHph_N38 (NC_070851) [78,126 nt]
Rhizobium phage RHph_I1_6 (NC_070852) [77,174 nt]
Rhizobium phage RHph_Y38 (NC_070853) [77,869 nt]
Rhizobium phage RHEph22 (NC_070855) [77,655 nt]
Rhizobium phage RHph_Y2_6 (NC_070854) [78,314 nt]
Rhizobium phage RHEph16 (NC_070856) [77,178 nt]
Stenotrophomonas phage Philippe (NC_070847) [74,717 nt]
Stenotrophomonas phage C121 (NC_070858) [73,045 nt]
Xanthomonas phage RiverRider (NC_048703) [76,335 nt]
Stenotrophomonas phage Pokken (NC_049463) [76,239 nt]
Stenotrophomonas phage Piffle (NC_070868) [76,332 nt]
Stenotrophomonas phage Paxi (NC_070867) [74,962 nt]
Alcaligenes phage vB_Af_QDWS595 (NC_070849) [70,466 nt]
Pseudomonas phage Zuri (NC_049456) [75,873 nt]
Erwinia phage phiEaP8 (NC_049510) [75,929 nt]
Erwinia phage vB_EamP_Frozen (NC_031062) [75,147 nt]
Erwinia phage Ea9-2 (NC_023579) [75,568 nt]
Pseudomonas phage inbricus (NC_054970) [70,211 nt]
Achromobacter phage vB_AxyP_19-32_Axy11 (NC_054972) [73,413 nt]
Achromobacter phage vB_AxyP_19-32_Axy10 (NC_054971) [73,898 nt]
Achromobacter phage JWDelta (KF787094) [73,659 nt]
Achromobacter phage JWAlpha (NC_023556) [72,329 nt]
Achromobacter phage phiAxp-3 (NC_028908) [72,825 nt]
Achromobacter phage vB_AxyP_19-32_Axy04 (NC_054967) [73,834 nt]
Achromobacter phage vB_AxyP_19-32_Axy24 (NC_054969) [74,744 nt]
Achromobacter phage vB_AxyP_19-32_Axy12 (NC_054968) [74,096 nt]
Klebsiella phage KP8 (NC_048700) [73,679 nt]
Escherichia phage N4 (NC_008720) [70,153 nt]
Shigella virus Moo19 (NC_070850) [72,458 nt]
Escherichia phage IME11 (NC_019423) [72,570 nt]
Escherichia phage vB_EcoP_G7C (NC_015933) [72,917 nt]
Escherichia phage phi G17 (NC_070870) [68,270 nt]
Escherichia phage vB_EcoP_SP5M (NC_070869) [72,896 nt]
Escherichia phage vB_EcoP_PhAPEC7 (NC_024790) [71,778 nt]
Escherichia phage vB_EcoP_PhAPEC5 (NC_024786) [71,248 nt]
Escherichia phage U1G (NC_070872) [73,275 nt]
Escherichia phage PGN829.1 (NC_070871) [74,437 nt]
Escherichia phage vB_EcoP-ZQ2 (NC_070874) [72,231 nt]
Shigella phage pSb-1 (NC_023589) [71,629 nt]
Escherichia phage St11Ph5 (NC_070873) [72,444 nt]
Escherichia phage EC1-UPM (NC_041906) [70,912 nt]
Escherichia phage ECBP1 (NC_018854) [69,855 nt]
Escherichia phage Bp4 (NC_024142) [72,583 nt]
Microbacterium phage Pumpernickel (NC_073468) [186,095 nt]
Bacillus phage Mgbh1 (NC_041879) [58,951 nt]
Yersinia phage phiR1-37 (NC_016163) [262,391 nt]
Bacillus phage PBS1 (NC_043027) [252,197 nt]
Bacillus phage AR9 (NC_031039) [251,042 nt]
Rhodococcus phage Weasels2 (NC_041887) [134,973 nt]
Rhodococcus phage Trina (NC_042040) [139,262 nt]
Bacillus phage Curly (NC_020479) [49,425 nt]
Bacillus phage Glittering (NC_022766) [49,246 nt]
Bacillus phage Gemini (KC330681) [49,362 nt]
Bacillus phage Andromeda (NC_020478) [49,259 nt]
Bacillus phage Riggi (NC_022765) [49,836 nt]
Bacillus phage Finn (NC_020480) [50,161 nt]
Bacillus phage Blastoid (NC_022773) [50,354 nt]

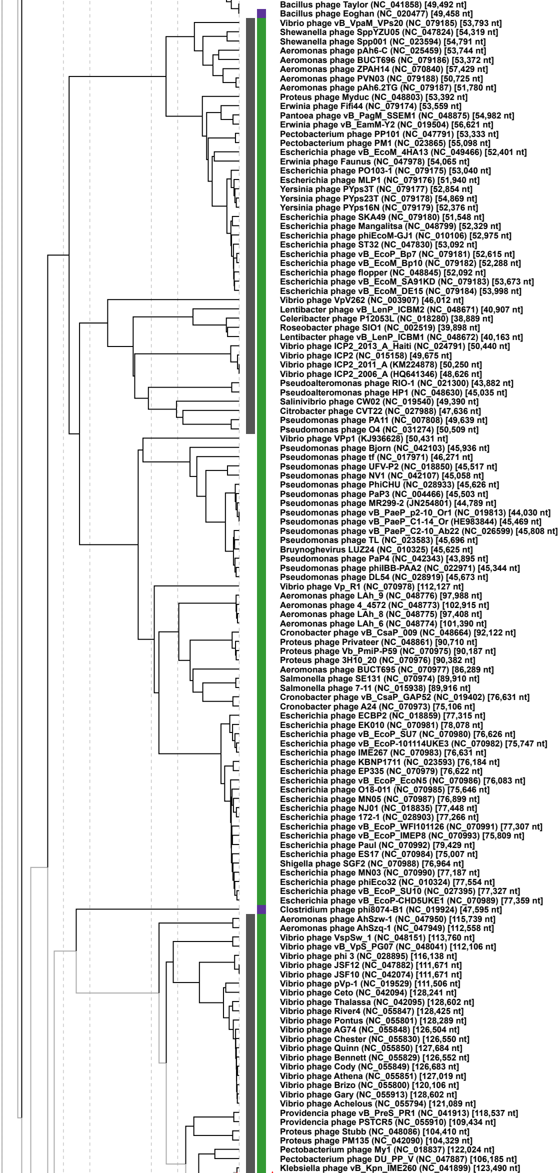

Bacillus phage Taylor (NC_041858) [49,492 nt]
Bacillus phage Eoghan (NC_020477) [49,458 nt]
Vibrio phage vB_VpaM_VPs20 (NC_079185) [53,793 nt]
Shewanella phage SppYZU05 (NC_047824) [54,319 nt]
Shewanella phage Spp001 (NC_023594) [54,791 nt]
Aeromonas phage pAh6-C (NC_025459) [53,744 nt]
Aeromonas phage BUCT696 (NC_079186) [53,372 nt]
Aeromonas phage ZPAH14 (NC_070840) [57,429 nt]
Aeromonas phage PVN03 (NC_079188) [50,725 nt]
Aeromonas phage pAh6.2TG (NC_079187) [51,780 nt]
Proteus phage Myduc (NC_048803) [53,392 nt]
Erwinia phage Fifi44 (NC_079174) [53,559 nt]
Pantoea phage vB_PagM_SSEM1 (NC_048875) [54,982 nt]
Erwinia phage vB_EamM-Y2 (NC_019504) [56,621 nt]
Pectobacterium phage PP101 (NC_047791) [53,333 nt]
Pectobacterium phage PM1 (NC_023865) [55,098 nt]
Escherichia phage vB_EcoM_4HA13 (NC_049466) [52,401 nt]
Erwinia phage Faunus (NC_047978) [54,065 nt]
Escherichia phage PO103-1 (NC_079175) [53,040 nt]
Escherichia phage MLP1 (NC_079176) [51,940 nt]
Yersinia phage PYps3T (NC_079177) [52,854 nt]
Yersinia phage PYps23T (NC_079178) [54,869 nt]
Yersinia phage PYps16N (NC_079179) [52,376 nt]
Escherichia phage SKA49 (NC_079180) [51,548 nt]
Escherichia phage Mangalitsa (NC_048799) [52,329 nt]
Escherichia phage phiEcoM-GJ1 (NC_010106) [52,975 nt]
Escherichia phage ST32 (NC_047830) [53,092 nt]
Escherichia phage vB_EcoP_Bp7 (NC_079181) [52,615 nt]
Escherichia phage vB_EcoM_Bp10 (NC_079182) [52,288 nt]
Escherichia phage flopper (NC_048845) [52,092 nt]
Escherichia phage vB_EcoM_SA91KD (NC_079183) [53,673 nt]
Escherichia phage vB_EcoM_DE15 (NC_079184) [53,998 nt]
Vibrio phage VpV262 (NC_003907) [46,012 nt]
Lentibacter phage vB_LenP_ICBM2 (NC_048671) [40,907 nt]
Celeribacter phage P12053L (NC_018280) [38,889 nt]
Roseobacter phage SIO1 (NC_002519) [39,898 nt]
Lentibacter phage vB_LenP_ICBM1 (NC_048672) [40,163 nt]
Vibrio phage ICP2_2013_A_Haiti (NC_024791) [50,440 nt]
Vibrio phage ICP2 (NC_015158) [49,675 nt]
Vibrio phage ICP2_2011_A (KM224878) [50,250 nt]
Vibrio phage ICP2_2006_A (HQ641346) [48,626 nt]
Pseudoalteromonas phage RIO-1 (NC_021300) [43,882 nt]
Pseudoalteromonas phage HP1 (NC_048630) [45,035 nt]
Salinivibrio phage CW02 (NC_019540) [49,390 nt]
Citrobacter phage CVT22 (NC_027988) [47,636 nt]
Pseudomonas phage PA11 (NC_007808) [49,639 nt]
Pseudomonas phage O4 (NC_031274) [50,509 nt]
Vibrio phage VPp1 (KJ936628) [50,431 nt]
Pseudomonas phage Bjorn (NC_042103) [45,936 nt]
Pseudomonas phage tf (NC_017971) [46,271 nt]
Pseudomonas phage UFV-P2 (NC_018850) [45,517 nt]
Pseudomonas phage NV1 (NC_042107) [45,058 nt]
Pseudomonas phage PhiCHU (NC_028933) [45,626 nt]
Pseudomonas phage PaP3 (NC_004466) [45,503 nt]
Pseudomonas phage MR299-2 (JN254801) [44,789 nt]
Pseudomonas phage vB_PaeP_p2-10_Or1 (NC_019813) [44,030 nt]
Pseudomonas phage vB_PaeP_C1-14_Or (HE983844) [45,469 nt]
Pseudomonas phage vB_PaeP_C2-10_Ab22 (NC_026599) [45,808 nt]
Pseudomonas phage TL (NC_023583) [45,696 nt]
Bruynoghevirus LUZ24 (NC_010325) [45,625 nt]
Pseudomonas phage PaP4 (NC_042343) [43,895 nt]
Pseudomonas phage philBB-PAA2 (NC_022971) [45,344 nt]
Pseudomonas phage DL54 (NC_028919) [45,673 nt]
Vibrio phage Vp_R1 (NC_070978) [112,127 nt]
Aeromonas phage LAh_9 (NC_048776) [97,988 nt]
Aeromonas phage 4_4572 (NC_048773) [102,915 nt]
Aeromonas phage LAh_8 (NC_048775) [97,408 nt]
Aeromonas phage LAh_6 (NC_048774) [101,390 nt]
Cronobacter phage vB_CsaP_009 (NC_048664) [92,122 nt]
Proteus phage Privateer (NC_048861) [90,710 nt]
Proteus phage Vb_PmiP-P59 (NC_070975) [90,187 nt]
Proteus phage 3H10_20 (NC_070976) [90,382 nt]
Aeromonas phage BUCT695 (NC_070977) [86,289 nt]
Salmonella phage SE131 (NC_070974) [89,910 nt]
Salmonella phage 7-11 (NC_015938) [89,916 nt]
Cronobacter phage vB_CsaP_GAP52 (NC_019402) [76,631 nt]
Cronobacter phage A24 (NC_070973) [75,106 nt]
Escherichia phage ECBP2 (NC_018859) [77,315 nt]
Escherichia phage EK010 (NC_070981) [78,078 nt]
Escherichia phage vB_EcoP_SU7 (NC_070980) [76,626 nt]
Escherichia phage vB_EcoP-101114UKE3 (NC_070982) [75,747 nt]
Escherichia phage IME267 (NC_070983) [76,631 nt]
Escherichia phage KBNP1711 (NC_023593) [76,184 nt]
Escherichia phage EP335 (NC_070979) [76,622 nt]
Escherichia phage vB_EcoP_EcoN5 (NC_070986) [76,083 nt]
Escherichia phage O18-011 (NC_070985) [75,646 nt]
Escherichia phage MN05 (NC_070987) [76,899 nt]
Escherichia phage NJ01 (NC_018835) [77,448 nt]
Escherichia phage 172-1 (NC_028903) [77,266 nt]
Escherichia phage vB_EcoP_WFI101126 (NC_070991) [77,307 nt]
Escherichia phage vB_EcoP_IMEP8 (NC_070993) [75,809 nt]
Escherichia phage Paul (NC_070992) [79,429 nt]
Escherichia phage ES17 (NC_070984) [75,007 nt]
Shigella phage SGF2 (NC_070988) [76,964 nt]
Escherichia phage MN03 (NC_070990) [77,187 nt]
Escherichia phage phiEco32 (NC_010324) [77,554 nt]
Escherichia phage vB_EcoP_SU10 (NC_027395) [77,327 nt]
Escherichia phage vB_EcoP-CHD5UKE1 (NC_070989) [77,359 nt]
Clostridium phage phi8074-B1 (NC_019924) [47,595 nt]
Aeromonas phage AhSzw-1 (NC_047950) [115,739 nt]
Aeromonas phage AhSzq-1 (NC_047949) [112,558 nt]
Vibrio phage VspSw_1 (NC_048151) [113,760 nt]
Vibrio phage vB_VpS_PG07 (NC_048041) [112,106 nt]
Vibrio phage phi 3 (NC_028895) [116,138 nt]
Vibrio phage JSF12 (NC_047882) [111,671 nt]
Vibrio phage JSF10 (NC_042074) [111,671 nt]
Vibrio phage pVp-1 (NC_019529) [111,506 nt]
Vibrio phage Ceto (NC_042094) [128,241 nt]
Vibrio phage Thalassa (NC_042095) [128,602 nt]
Vibrio phage River4 (NC_055847) [128,425 nt]
Vibrio phage Pontus (NC_055801) [128,289 nt]
Vibrio phage AG74 (NC_055848) [126,504 nt]
Vibrio phage Chester (NC_055830) [126,550 nt]
Vibrio phage Quinn (NC_055850) [127,684 nt]
Vibrio phage Bennett (NC_055829) [126,552 nt]
Vibrio phage Cody (NC_055849) [126,683 nt]
Vibrio phage Athena (NC_055851) [127,019 nt]
Vibrio phage Brizo (NC_055800) [120,106 nt]
Vibrio phage Gary (NC_055913) [128,602 nt]
Vibrio phage Achelous (NC_055794) [121,089 nt]
Providencia phage vB_PreS_PR1 (NC_041913) [118,537 nt]
Providencia phage PSTCR5 (NC_055910) [109,434 nt]
Proteus phage Stubb (NC_048086) [104,410 nt]
Proteus phage PM135 (NC_042090) [104,329 nt]
Pectobacterium phage My1 (NC_018837) [122,024 nt]
Pectobacterium phage DU_PP_V (NC_047887) [106,185 nt]
Klebsiella phage vB_Kpn_IME260 (NC_041899) [123,490 nt]

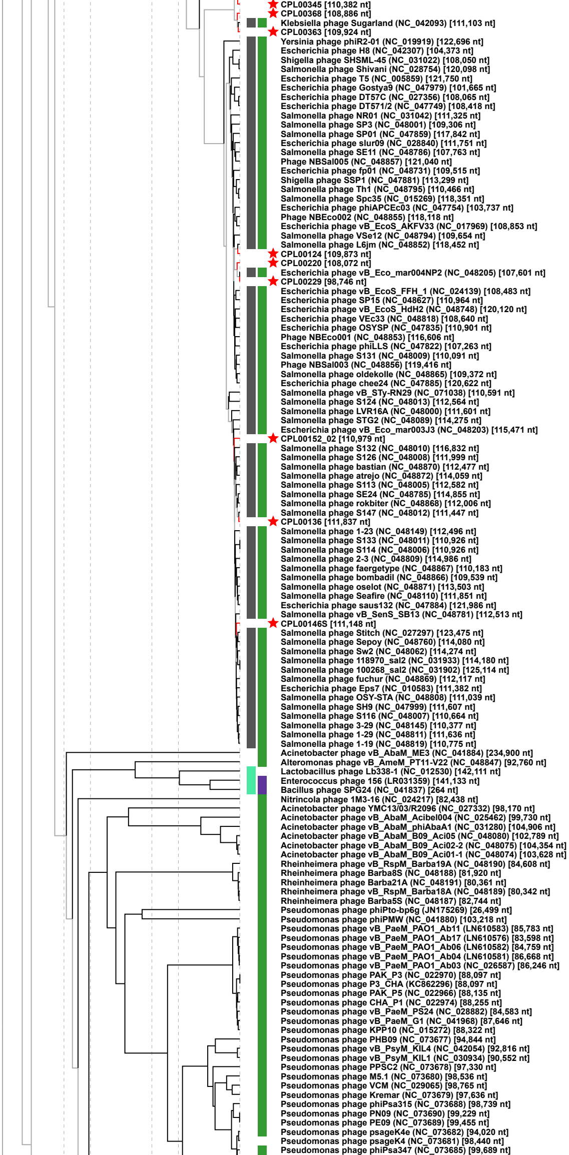
CPL00345 [110,382 nt]
CPL00368 [108,886 nt]
Klebsiella phage Sugarland (NC_042093) [111,103 nt]
CPL00363 [109,924 nt]
Yersinia phage phiR2-01 (NC_019919) [122,696 nt]
Escherichia phage H8 (NC_042307) [104,373 nt]
Shigella phage SHSML-45 (NC_031022) [108,050 nt]
Salmonella phage Shivani (NC_028754) [120,098 nt]
Escherichia phage T5 (NC_005859) [121,750 nt]
Escherichia phage Gostya9 (NC_047979) [101,665 nt]
Escherichia phage DT57C (NC_027356) [108,065 nt]
Escherichia phage DT571/2 (NC_047749) [108,418 nt]
Salmonella phage NR01 (NC_031042) [111,325 nt]
Salmonella phage SP3 (NC_048001) [109,306 nt]
Salmonella phage SP01 (NC_047859) [117,842 nt]
Escherichia phage slur09 (NC_028840) [111,751 nt]
Salmonella phage SE11 (NC_048786) [107,763 nt]
Phage NBSal005 (NC_048857) [121,040 nt]
Escherichia phage fp01 (NC_048731) [109,515 nt]
Shigella phage SSP1 (NC_047881) [113,299 nt]
Salmonella phage Th1 (NC_048795) [110,466 nt]
Salmonella phage Spc35 (NC_015269) [118,351 nt]
Escherichia phage phiAPCEc03 (NC_047754) [103,737 nt]
Phage NBEco002 (NC_048855) [118,118 nt]
Escherichia phage vB_EcoS_AKFV33 (NC_017969) [108,853 nt]
Salmonella phage VSe12 (NC_048794) [109,654 nt]
Salmonella phage L6jm (NC_048852) [118,452 nt]
CPL00124 [109,873 nt]
CPL00220 [108,072 nt]
Escherichia phage vB_Eco_mar004NP2 (NC_048205) [107,601 nt]
CPL00229 [98,746 nt]
Escherichia phage vB_EcoS_FFH_1 (NC_024139) [108,483 nt]
Escherichia phage SP15 (NC_048627) [110,964 nt]
Escherichia phage vB_EcoS_HdH2 (NC_048748) [120,120 nt]
Escherichia phage VEc33 (NC_048818) [108,640 nt]
Escherichia phage OSYSP (NC_047835) [110,901 nt]
Phage NBEco001 (NC_048853) [116,606 nt]
Escherichia phage phiLLS (NC_047822) [107,263 nt]
Salmonella phage S131 (NC_048009) [110,091 nt]
Phage NBSal003 (NC_048856) [119,416 nt]
Salmonella phage oldekolle (NC_048865) [109,372 nt]
Escherichia phage chee24 (NC_047885) [120,622 nt]
Salmonella phage vB_STy-RN29 (NC_071038) [110,591 nt]
Salmonella phage S124 (NC_048013) [112,564 nt]
Salmonella phage LVR16A (NC_048000) [111,601 nt]
Salmonella phage STG2 (NC_048089) [114,275 nt]
Escherichia phage vB_Eco_mar003J3 (NC_048203) [115,471 nt]
CPL00152_02 [110,979 nt]
Salmonella phage S132 (NC_048010) [116,832 nt]
Salmonella phage S126 (NC_048008) [111,999 nt]
Salmonella phage bastian (NC_048870) [112,477 nt]
Salmonella phage atrejo (NC_048872) [114,059 nt]
Salmonella phage S113 (NC_048005) [112,582 nt]
Salmonella phage SE24 (NC_048785) [114,855 nt]
Salmonella phage rokbiter (NC_048868) [112,006 nt]
Salmonella phage S147 (NC_048012) [111,447 nt]
CPL00136 [111,837 nt]
Salmonella phage 1-23 (NC_048149) [112,496 nt]
Salmonella phage S133 (NC_048011) [110,926 nt]
Salmonella phage S114 (NC_048006) [110,926 nt]
Salmonella phage 2-3 (NC_048809) [114,986 nt]
Salmonella phage faergetype (NC_048867) [110,183 nt]
Salmonella phage bombadil (NC_048866) [109,539 nt]
Salmonella phage oselot (NC_048871) [113,503 nt]
Salmonella phage Seafire (NC_048110) [111,851 nt]
Escherichia phage saus132 (NC_047884) [121,986 nt]
Salmonella phage vB_SenS_SB13 (NC_048781) [112,513 nt]
CPL00146S [111,148 nt]
Salmonella phage Stitch (NC_027297) [123,475 nt]
Salmonella phage Sepoy (NC_048760) [114,080 nt]
Salmonella phage Sw2 (NC_048062) [114,274 nt]
Salmonella phage 118970_sal2 (NC_031933) [114,180 nt]
Salmonella phage 100268_sal2 (NC_031902) [125,114 nt]
Salmonella phage fuchur (NC_048869) [112,117 nt]
Escherichia phage Eps7 (NC_010583) [111,382 nt]
Salmonella phage OSY-STA (NC_048808) [111,039 nt]
Salmonella phage SH9 (NC_047999) [111,607 nt]
Salmonella phage S116 (NC_048007) [110,664 nt]
Salmonella phage 3-29 (NC_048145) [110,377 nt]
Salmonella phage 1-29 (NC_048811) [111,636 nt]
Salmonella phage 1-19 (NC_048819) [110,775 nt]
Acinetobacter phage vB_AbaM_ME3 (NC_041884) [234,900 nt]
Alteromonas phage vB_AmeM_PT11-V22 (NC_048847) [92,760 nt]
Lactobacillus phage Lb338-1 (NC_012530) [142,111 nt]
Enterococcus phage 156 (LR031359) [141,133 nt]
Bacillus phage SPG24 (NC_041837) [264 nt]
Nitrincola phage 1M3-16 (NC_024217) [82,438 nt]
Acinetobacter phage YMC13/03/R2096 (NC_027332) [98,170 nt]
Acinetobacter phage vB_AbaM_Acibel004 (NC_025462) [99,730 nt]
Acinetobacter phage vB_AbaM_phiAbaA1 (NC_031280) [104,906 nt]
Acinetobacter phage vB_AbaM_B09_Aci05 (NC_048080) [102,789 nt]
Acinetobacter phage vB_AbaM_B09_Aci02-2 (NC_048075) [104,354 nt]
Acinetobacter phage vB_AbaM_B09_Aci01-1 (NC_048074) [103,628 nt]
Rheinheimera phage vB_RspM_Barba19A (NC_048190) [84,608 nt]
Rheinheimera phage Barba8S (NC_048188) [81,920 nt]
Rheinheimera phage Barba21A (NC_048191) [80,361 nt]
Rheinheimera phage vB_RspM_Barba18A (NC_048189) [80,342 nt]
Rheinheimera phage Barba5S (NC_048187) [82,744 nt]
Pseudomonas phage phiPto-bp6g (JN175269) [26,499 nt]
Pseudomonas phage phiPMW (NC_041880) [103,218 nt]
Pseudomonas phage vB_PaeM_PAO1_Ab11 (LN610583) [85,783 nt]
Pseudomonas phage vB_PaeM_PAO1_Ab17 (LN610576) [83,598 nt]
Pseudomonas phage vB_PaeM_PAO1_Ab06 (LN610582) [84,759 nt]
Pseudomonas phage vB_PaeM_PAO1_Ab04 (LN610581) [86,668 nt]
Pseudomonas phage vB_PaeM_PAO1_Ab03 (NC_026587) [86,246 nt]
Pseudomonas phage PAK_P3 (NC_022970) [88,097 nt]
Pseudomonas phage P3_CHA (KC862296) [88,097 nt]
Pseudomonas phage PAK_P5 (NC_022966) [88,135 nt]
Pseudomonas phage CHA_P1 (NC_022974) [88,255 nt]
Pseudomonas phage vB_PaeM_PS24 (NC_028882) [84,583 nt]
Pseudomonas phage vB_PaeM_G1 (NC_041968) [87,646 nt]
Pseudomonas phage KPP10 (NC_015272) [88,322 nt]
Pseudomonas phage PHB09 (NC_073677) [94,844 nt]
Pseudomonas phage vB_PsyM_KIL4 (NC_042054) [92,816 nt]
Pseudomonas phage vB_PsyM_KIL1 (NC_030934) [90,552 nt]
Pseudomonas phage PPSC2 (NC_073678) [97,330 nt]
Pseudomonas phage M5.1 (NC_073680) [98,536 nt]
Pseudomonas phage VCM (NC_029065) [98,765 nt]
Pseudomonas phage Kremar (NC_073679) [97,636 nt]
Pseudomonas phage phiPsa315 (NC_073688) [98,739 nt]
Pseudomonas phage PN09 (NC_073690) [99,229 nt]
Pseudomonas phage PE09 (NC_073689) [99,455 nt]
Pseudomonas phage psageK4e (NC_073682) [94,020 nt]
Pseudomonas phage psageK4 (NC_073681) [98,440 nt]
Pseudomonas phage phiPsa347 (NC_073685) [99,689 nt]

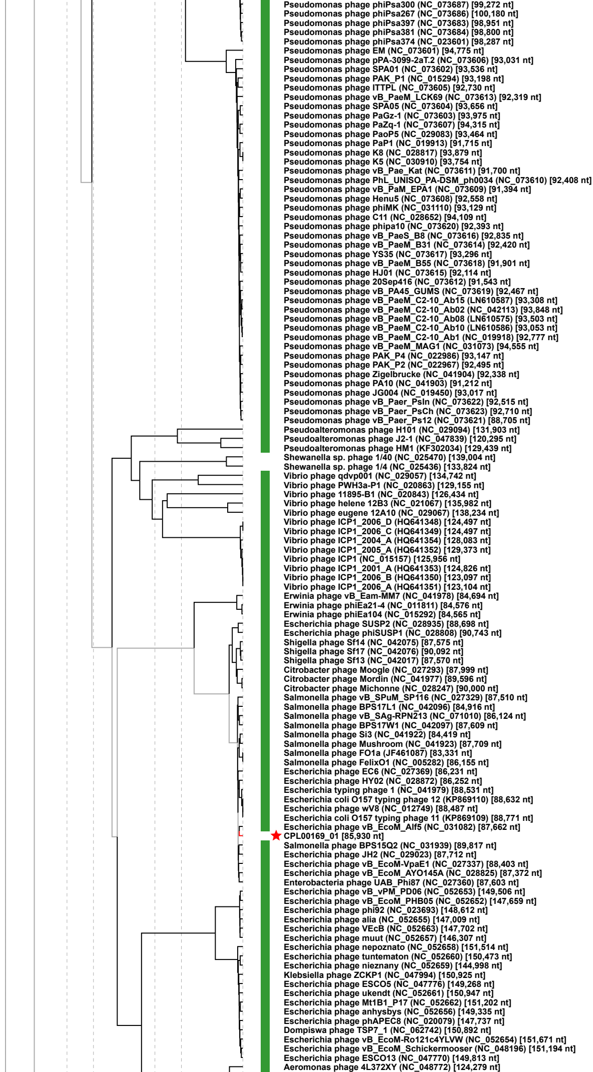

Pseudomonas phage phiPsa300 (NC_073687) [99,272 nt]
Pseudomonas phage phiPsa267 (NC_073686) [100,180 nt]
Pseudomonas phage phiPsa397 (NC_073683) [98,951 nt]
Pseudomonas phage phiPsa381 (NC_073684) [98,800 nt]
Pseudomonas phage phiPsa374 (NC_023601) [98,287 nt]
Pseudomonas phage EM (NC_073601) [94,775 nt]
Pseudomonas phage pPA-3099-2aT.2 (NC_073606) [93,031 nt]
Pseudomonas phage SPA01 (NC_073602) [93,536 nt]
Pseudomonas phage PAK_P1 (NC_015294) [93,198 nt]
Pseudomonas phage ITTPL (NC_073605) [92,730 nt]
Pseudomonas phage vB_PaeM_LCK69 (NC_073613) [92,319 nt]
Pseudomonas phage SPA05 (NC_073604) [93,656 nt]
Pseudomonas phage PaGz-1 (NC_073603) [93,975 nt]
Pseudomonas phage PaZq-1 (NC_073607) [94,315 nt]
Pseudomonas phage PaoP5 (NC_029083) [93,464 nt]
Pseudomonas phage PaP1 (NC_019913) [91,715 nt]
Pseudomonas phage K8 (NC_028817) [93,879 nt]
Pseudomonas phage K5 (NC_030910) [93,754 nt]
Pseudomonas phage vB_Pae_Kat (NC_073611) [91,700 nt]
Pseudomonas phage PhL_UNISO_PA-DSM_ph0034 (NC_073610) [92,408 nt]
Pseudomonas phage vB_PaM_EPA1 (NC_073609) [91,394 nt]
Pseudomonas phage Henu5 (NC_073608) [92,558 nt]
Pseudomonas phage phiMK (NC_031110) [93,129 nt]
Pseudomonas phage C11 (NC_028652) [94,109 nt]
Pseudomonas phage phipa10 (NC_073620) [92,393 nt]
Pseudomonas phage vB_PaeS_B8 (NC_073616) [92,835 nt]
Pseudomonas phage vB_PaeM_B31 (NC_073614) [92,420 nt]
Pseudomonas phage YS35 (NC_073617) [93,296 nt]
Pseudomonas phage vB_PaeM_B55 (NC_073618) [91,901 nt]
Pseudomonas phage HJ01 (NC_073615) [92,114 nt]
Pseudomonas phage 20Sep416 (NC_073612) [91,543 nt]
Pseudomonas phage vB_PA45_GUMS (NC_073619) [92,467 nt]
Pseudomonas phage vB_PaeM_C2-10_Ab15 (LN610587) [93,308 nt]
Pseudomonas phage vB_PaeM_C2-10_Ab02 (NC_042113) [93,848 nt]
Pseudomonas phage vB_PaeM_C2-10_Ab08 (LN610575) [93,503 nt]
Pseudomonas phage vB_PaeM_C2-10_Ab10 (LN610586) [93,053 nt]
Pseudomonas phage vB_PaeM_C2-10_Ab1 (NC_019918) [92,777 nt]
Pseudomonas phage vB_PaeM_MAG1 (NC_031073) [94,555 nt]
Pseudomonas phage PAK_P4 (NC_022986) [93,147 nt]
Pseudomonas phage PAK_P2 (NC_022967) [92,495 nt]
Pseudomonas phage Zigelbrucke (NC_041904) [92,338 nt]
Pseudomonas phage PA10 (NC_041903) [91,212 nt]
Pseudomonas phage JG004 (NC_019450) [93,017 nt]
Pseudomonas phage vB_Paer_PsIn (NC_073622) [92,515 nt]
Pseudomonas phage vB_Paer_PsCh (NC_073623) [92,710 nt]
Pseudomonas phage vB_Paer_Ps12 (NC_073621) [88,705 nt]
Pseudoalteromonas phage H101 (NC_029094) [131,903 nt]
Pseudoalteromonas phage J2-1 (NC_047839) [120,295 nt]
Pseudoalteromonas phage HM1 (KF302034) [129,439 nt]
Shewanella sp. phage 1/40 (NC_025470) [139,004 nt]
Shewanella sp. phage 1/4 (NC_025436) [133,824 nt]
Vibrio phage qdvp001 (NC_029057) [134,742 nt]
Vibrio phage PWH3a-P1 (NC_020863) [129,155 nt]
Vibrio phage 11895-B1 (NC_020843) [126,434 nt]
Vibrio phage helene 12B3 (NC_021067) [135,982 nt]
Vibrio phage eugene 12A10 (NC_029067) [138,234 nt]
Vibrio phage ICP1_2006_D (HQ641348) [124,497 nt]
Vibrio phage ICP1_2006_C (HQ641349) [124,497 nt]
Vibrio phage ICP1_2004_A (HQ641354) [128,083 nt]
Vibrio phage ICP1_2005_A (HQ641352) [129,373 nt]
Vibrio phage ICP1 (NC_015157) [125,956 nt]
Vibrio phage ICP1_2001_A (HQ641353) [124,826 nt]
Vibrio phage ICP1_2006_B (HQ641350) [123,097 nt]
Vibrio phage ICP1_2006_A (HQ641351) [123,104 nt]
Erwinia phage vB_Eam-MM7 (NC_041978) [84,694 nt]
Erwinia phage phiEa21-4 (NC_011811) [84,576 nt]
Erwinia phage phiEa104 (NC_015292) [84,565 nt]
Escherichia phage SUSP2 (NC_028935) [88,698 nt]
Escherichia phage phiSUSP1 (NC_028808) [90,743 nt]
Shigella phage Sf14 (NC_042075) [87,575 nt]
Shigella phage Sf17 (NC_042076) [90,092 nt]
Shigella phage Sf13 (NC_042017) [87,570 nt]
Citrobacter phage Moogle (NC_027293) [87,999 nt]
Citrobacter phage Mordin (NC_041977) [89,596 nt]
Citrobacter phage Michonne (NC_028247) [90,000 nt]
Salmonella phage vB_SPuM_SP116 (NC_027329) [87,510 nt]
Salmonella phage BPS17L1 (NC_042096) [84,916 nt]
Salmonella phage vB_SAg-RPN213 (NC_071010) [86,124 nt]
Salmonella phage BPS17W1 (NC_042097) [87,609 nt]
Salmonella phage Si3 (NC_041922) [84,419 nt]
Salmonella phage Mushroom (NC_041923) [87,709 nt]
Salmonella phage FO1a (JF461087) [83,331 nt]
Salmonella phage FelixO1 (NC_005282) [86,155 nt]
Escherichia phage EC6 (NC_027369) [86,231 nt]
Escherichia phage HY02 (NC_028872) [86,252 nt]
Escherichia typing phage 1 (NC_041979) [88,531 nt]
Escherichia coli O157 typing phage 12 (KP869110) [88,632 nt]
Escherichia phage wV8 (NC_012749) [88,487 nt]
Escherichia coli O157 typing phage 11 (KP869109) [88,771 nt]
Escherichia phage vB_EcoM_Alf5 (NC_031082) [87,662 nt]
CPL00169_01 [85,930 nt]
Salmonella phage BPS15Q2 (NC_031939) [89,817 nt]
Escherichia phage JH2 (NC_029023) [87,712 nt]
Escherichia phage vB_EcoM-VpaE1 (NC_027337) [88,403 nt]
Escherichia phage vB_EcoM_AYO145A (NC_028825) [87,372 nt]
Enterobacteria phage UAB_Phi87 (NC_027360) [87,603 nt]
Escherichia phage vB_vPM_PD06 (NC_052653) [149,506 nt]
Escherichia phage vB_EcoM_PHB05 (NC_052652) [147,659 nt]
Escherichia phage phi92 (NC_023693) [148,612 nt]
Escherichia phage alia (NC_052655) [147,009 nt]
Escherichia phage VEcB (NC_052663) [147,702 nt]
Escherichia phage muut (NC_052657) [146,307 nt]
Escherichia phage nepoznato (NC_052658) [151,514 nt]
Escherichia phage tuntematon (NC_052660) [150,473 nt]
Escherichia phage nieznany (NC_052659) [144,998 nt]
Klebsiella phage ZCKP1 (NC_047994) [150,925 nt]
Escherichia phage ESCO5 (NC_047776) [149,268 nt]
Escherichia phage ukendt (NC_052661) [150,947 nt]
Escherichia phage Mt1B1_P17 (NC_052662) [151,202 nt]
Escherichia phage anhysbys (NC_052656) [149,335 nt]
Escherichia phage phAPEC8 (NC_020079) [147,737 nt]
Dompiswa phage TSP7_1 (NC_062742) [150,892 nt]
Escherichia phage vB_EcoM-Ro121c4YLVW (NC_052654) [151,671 nt]
Escherichia phage vB_EcoM_Schickermooser (NC_048196) [151,194 nt]
Escherichia phage ESCO13 (NC_047770) [149,813 nt]
Aeromonas phage 4L372XY (NC_048772) [124,279 nt]

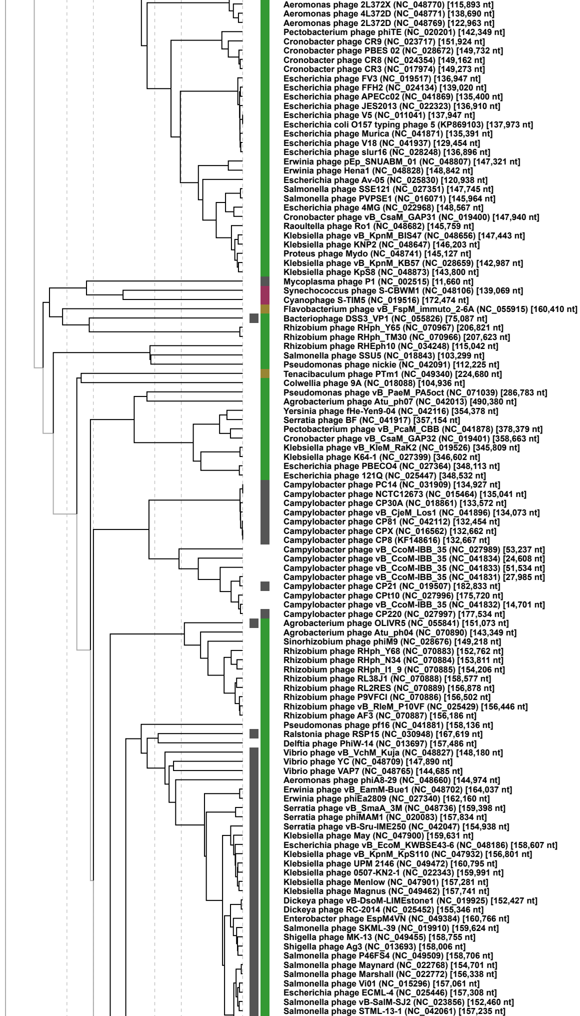
Aeromonas phage 2L372X (NC_048770) [115,893 nt]
Aeromonas phage 4L372D (NC_048771) [138,690 nt]
Aeromonas phage 2L372D (NC_048769) [122,963 nt]
Pectobacterium phage phiTE (NC_020201) [142,349 nt]
Cronobacter phage CR9 (NC_023717) [151,924 nt]
Cronobacter phage PBES 02 (NC_028672) [149,732 nt]
Cronobacter phage CR8 (NC_024354) [149,162 nt]
Cronobacter phage CR3 (NC_017974) [149,273 nt]
Escherichia phage FV3 (NC_019517) [136,947 nt]
Escherichia phage FFH2 (NC_024134) [139,020 nt]
Escherichia phage APECc02 (NC_041869) [135,400 nt]
Escherichia phage JES2013 (NC_022323) [136,910 nt]
Escherichia phage V5 (NC_011041) [137,947 nt]
Escherichia coli O157 typing phage 5 (KP869103) [137,973 nt]
Escherichia phage Murica (NC_041871) [135,391 nt]
Escherichia phage V18 (NC_041937) [129,454 nt]
Escherichia phage slur16 (NC_028248) [136,896 nt]
Erwinia phage pEp_SNUABM_01 (NC_048807) [147,321 nt]
Erwinia phage Hena1 (NC_048828) [148,842 nt]
Escherichia phage Av-05 (NC_025830) [120,938 nt]
Salmonella phage SSE121 (NC_027351) [147,745 nt]
Salmonella phage PVPSE1 (NC_016071) [145,964 nt]
Escherichia phage 4MG (NC_022968) [148,567 nt]
Cronobacter phage vB_CsaM_GAP31 (NC_019400) [147,940 nt]
Raoultella phage Ro1 (NC_048682) [145,759 nt]
Klebsiella phage vB_KpnM_BIS47 (NC_048656) [147,443 nt]
Klebsiella phage KNP2 (NC_048647) [146,203 nt]
Proteus phage Mydo (NC_048741) [145,127 nt]
Klebsiella phage vB_KpnM_KB57 (NC_028659) [142,987 nt]
Klebsiella phage KpS8 (NC_048873) [143,800 nt]
Mycoplasma phage P1 (NC_002515) [11,660 nt]
Synechococcus phage S-CBWM1 (NC_048106) [139,069 nt]
Cyanophage S-TIM5 (NC_019516) [172,474 nt]
Flavobacterium phage vB_FspM_immuto_2-6A (NC_055915) [160,410 nt]
Bacteriophage DSS3_VP1 (NC_055826) [75,087 nt]
Rhizobium phage RHph_Y65 (NC_070967) [206,821 nt]
Rhizobium phage RHph_TM30 (NC_070966) [207,623 nt]
Rhizobium phage RHEph10 (NC_034248) [115,042 nt]
Salmonella phage SSU5 (NC_018843) [103,299 nt]
Pseudomonas phage nickie (NC_042091) [112,225 nt]
Tenacibaculum phage PTm1 (NC_049340) [224,680 nt]
Colwellia phage 9A (NC_018088) [104,936 nt]
Pseudomonas phage vB_PaeM_PA5oct (NC_071039) [286,783 nt]
Agrobacterium phage Atu_ph07 (NC_042013) [490,380 nt]
Yersinia phage fHe-Yen9-04 (NC_042116) [354,378 nt]
Serratia phage BF (NC_041917) [357,154 nt]
Pectobacterium phage vB_PcaM_CBB (NC_041878) [378,379 nt]
Cronobacter phage vB_CsaM_GAP32 (NC_019401) [358,663 nt]
Klebsiella phage vB_KleM_RaK2 (NC_019526) [345,809 nt]
Klebsiella phage K64-1 (NC_027399) [346,602 nt]
Escherichia phage PBECO4 (NC_027364) [348,113 nt]
Escherichia phage 121Q (NC_025447) [348,532 nt]
Campylobacter phage PC14 (NC_031909) [134,927 nt]
Campylobacter phage NCTC12673 (NC_015464) [135,041 nt]
Campylobacter phage CP30A (NC_018861) [133,572 nt]
Campylobacter phage vB_CjeM_Los1 (NC_041896) [134,073 nt]
Campylobacter phage CP81 (NC_042112) [132,454 nt]
Campylobacter phage CPX (NC_016562) [132,662 nt]
Campylobacter phage CP8 (KF148616) [132,667 nt]
Campylobacter phage vB_CcoM-IBB_35 (NC_027989) [53,237 nt]
Campylobacter phage vB_CcoM-IBB_35 (NC_041834) [24,608 nt]
Campylobacter phage vB_CcoM-IBB_35 (NC_041833) [51,534 nt]
Campylobacter phage vB_CcoM-IBB_35 (NC_041831) [27,985 nt]
Campylobacter phage CP21 (NC_019507) [182,833 nt]
Campylobacter phage CPt10 (NC_027996) [175,720 nt]
Campylobacter phage vB_CcoM-IBB_35 (NC_041832) [14,701 nt]
Campylobacter phage CP220 (NC_027997) [177,534 nt]
Agrobacterium phage OLIVR5 (NC_055841) [151,073 nt]
Agrobacterium phage Atu_ph04 (NC_070890) [143,349 nt]
Sinorhizobium phage phiM9 (NC_028676) [149,218 nt]
Rhizobium phage RHph_Y68 (NC_070883) [152,762 nt]
Rhizobium phage RHph_N34 (NC_070884) [153,811 nt]
Rhizobium phage RHph_I1_9 (NC_070885) [154,206 nt]
Rhizobium phage RL38J1 (NC_070888) [158,577 nt]
Rhizobium phage RL2RES (NC_070889) [156,878 nt]
Rhizobium phage P9VFCI (NC_070886) [156,502 nt]
Rhizobium phage vB_RleM_P10VF (NC_025429) [156,446 nt]
Rhizobium phage AF3 (NC_070887) [156,186 nt]
Pseudomonas phage pf16 (NC_041881) [158,136 nt]
Ralstonia phage RSP15 (NC_030948) [167,619 nt]
Delftia phage PhiW-14 (NC_013697) [157,486 nt]
Vibrio phage vB_VchM_Kuja (NC_048827) [148,180 nt]
Vibrio phage YC (NC_048709) [147,890 nt]
Vibrio phage VAP7 (NC_048765) [144,685 nt]
Aeromonas phage phiA8-29 (NC_048660) [144,974 nt]
Erwinia phage vB_EamM-Bue1 (NC_048702) [164,037 nt]
Erwinia phage phiEa2809 (NC_027340) [162,160 nt]
Serratia phage vB_SmaA_3M (NC_048736) [159,398 nt]
Serratia phage phiMAM1 (NC_020083) [157,834 nt]
Serratia phage vB-Sru-IME250 (NC_042047) [154,938 nt]
Klebsiella phage May (NC_047900) [159,631 nt]
Escherichia phage vB_EcoM_KWBSE43-6 (NC_048186) [158,607 nt]
Klebsiella phage vB_KpnM_KpS110 (NC_047932) [156,801 nt]
Klebsiella phage UPM 2146 (NC_049472) [160,795 nt]
Klebsiella phage 0507-KN2-1 (NC_022343) [159,991 nt]
Klebsiella phage Menlow (NC_047901) [157,281 nt]
Klebsiella phage Magnus (NC_049462) [157,741 nt]
Dickeya phage vB-DsoM-LIMEstone1 (NC_019925) [152,427 nt]
Dickeya phage RC-2014 (NC_025452) [155,346 nt]
Enterobacter phage EspM4VN (NC_049384) [160,766 nt]
Salmonella phage SKML-39 (NC_019910) [159,624 nt]
Shigella phage MK-13 (NC_049455) [158,755 nt]
Shigella phage Ag3 (NC_013693) [158,006 nt]
Salmonella phage P46FS4 (NC_049509) [158,706 nt]
Salmonella phage Maynard (NC_022768) [154,701 nt]
Salmonella phage Marshall (NC_022772) [156,338 nt]
Salmonella phage Vi01 (NC_015296) [157,061 nt]
Escherichia phage ECML-4 (NC_025446) [157,308 nt]
Salmonella phage vB-SalM-SJ2 (NC_023856) [152,460 nt]
Salmonella phage STML-13-1 (NC_042061) [157,235 nt]

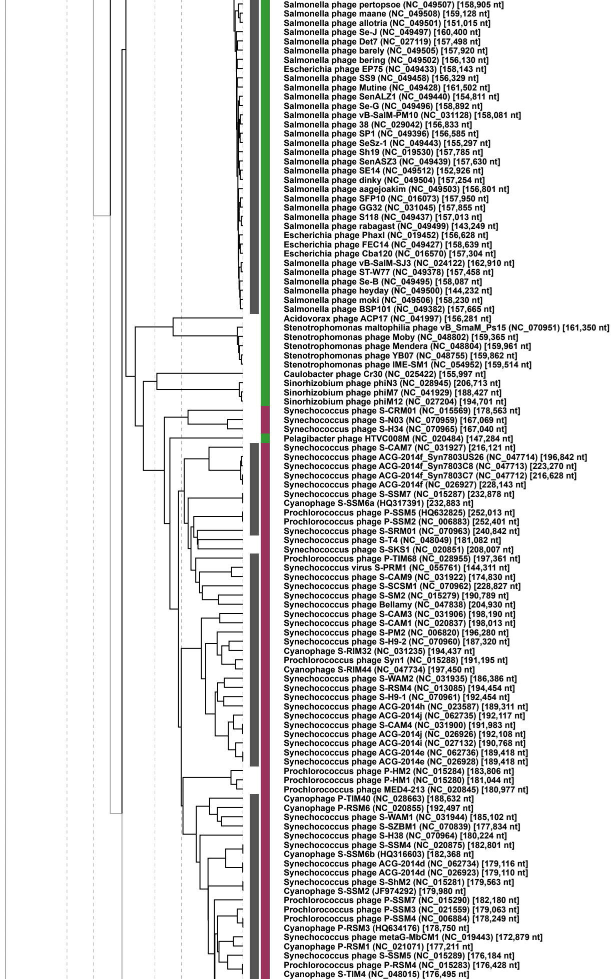

Salmonella phage pertopsoe (NC_049507) [158,905 nt]
Salmonella phage maane (NC_049508) [159,128 nt]
Salmonella phage allotria (NC_049501) [151,015 nt]
Salmonella phage Se-J (NC_049497) [160,400 nt]
Salmonella phage Det7 (NC_027119) [157,498 nt]
Salmonella phage barely (NC_049505) [157,920 nt]
Salmonella phage bering (NC_049502) [156,130 nt]
Escherichia phage EP75 (NC_049433) [158,143 nt]
Salmonella phage SS9 (NC_049458) [156,329 nt]
Salmonella phage Mutine (NC_049428) [161,502 nt]
Salmonella phage SenALZ1 (NC_049440) [154,811 nt]
Salmonella phage Se-G (NC_049496) [158,892 nt]
Salmonella phage vB-SalM-PM10 (NC_031128) [158,081 nt]
Salmonella phage 38 (NC_029042) [156,833 nt]
Salmonella phage SP1 (NC_049396) [156,585 nt]
Salmonella phage SeSz-1 (NC_049443) [155,297 nt]
Salmonella phage Sh19 (NC_019530) [157,785 nt]
Salmonella phage SenASZ3 (NC_049439) [157,630 nt]
Salmonella phage SE14 (NC_049512) [152,926 nt]
Salmonella phage dinky (NC_049504) [157,254 nt]
Salmonella phage aagejoakim (NC_049503) [156,801 nt]
Salmonella phage SFP10 (NC_016073) [157,950 nt]
Salmonella phage GG32 (NC_031045) [157,855 nt]
Salmonella phage S118 (NC_049437) [157,013 nt]
Salmonella phage rabagast (NC_049499) [143,249 nt]
Escherichia phage PhaxI (NC_019452) [156,628 nt]
Escherichia phage FEC14 (NC_049427) [158,639 nt]
Escherichia phage Cba120 (NC_016570) [157,304 nt]
Salmonella phage vB-SalM-SJ3 (NC_024122) [162,910 nt]
Salmonella phage ST-W77 (NC_049378) [157,458 nt]
Salmonella phage Se-B (NC_049495) [158,087 nt]
Salmonella phage heyday (NC_049500) [144,232 nt]
Salmonella phage moki (NC_049506) [158,230 nt]
Salmonella phage BSP101 (NC_049382) [157,665 nt]
Acidovorax phage ACP17 (NC_041997) [156,281 nt]
Stenotrophomonas maltophilia phage vB_SmaM_Ps15 (NC_070951) [161,350 nt]
Stenotrophomonas phage Moby (NC_048802) [159,365 nt]
Stenotrophomonas phage Mendera (NC_048804) [159,961 nt]
Stenotrophomonas phage YB07 (NC_048755) [159,862 nt]
Stenotrophomonas phage IME-SM1 (NC_054952) [159,514 nt]
Caulobacter phage Cr30 (NC_025422) [155,997 nt]
Sinorhizobium phage phiN3 (NC_028945) [206,713 nt]
Sinorhizobium phage phiM7 (NC_041929) [188,427 nt]
Sinorhizobium phage phiM12 (NC_027204) [194,701 nt]
Synechococcus phage S-CRM01 (NC_015569) [178,563 nt]
Synechococcus phage S-N03 (NC_070959) [167,069 nt]
Synechococcus phage S-H34 (NC_070965) [167,040 nt]
Pelagibacter phage HTVC008M (NC_020484) [147,284 nt]
Synechococcus phage S-CAM7 (NC_031927) [216,121 nt]
Synechococcus phage ACG-2014f_Syn7803US26 (NC_047714) [196,842 nt]
Synechococcus phage ACG-2014f_Syn7803C8 (NC_047713) [223,270 nt]
Synechococcus phage ACG-2014f_Syn7803C7 (NC_047712) [216,628 nt]
Synechococcus phage ACG-2014f (NC_026927) [228,143 nt]
Synechococcus phage S-SSM7 (NC_015287) [232,878 nt]
Cyanophage S-SSM6a (HQ317391) [232,883 nt]
Prochlorococcus phage P-SSM5 (HQ632825) [252,013 nt]
Prochlorococcus phage P-SSM2 (NC_006883) [252,401 nt]
Synechococcus phage S-SRM01 (NC_070963) [240,842 nt]
Synechococcus phage S-T4 (NC_048049) [181,082 nt]
Synechococcus phage S-SKS1 (NC_020851) [208,007 nt]
Prochlorococcus phage P-TIM68 (NC_028955) [197,361 nt]
Synechococcus virus S-PRM1 (NC_055761) [144,311 nt]
Synechococcus phage S-CAM9 (NC_031922) [174,830 nt]
Synechococcus phage S-SCSM1 (NC_070962) [228,827 nt]
Synechococcus phage S-SM2 (NC_015279) [190,789 nt]
Synechococcus phage Bellamy (NC_047838) [204,930 nt]
Synechococcus phage S-CAM3 (NC_031906) [198,190 nt]
Synechococcus phage S-CAM1 (NC_020837) [198,013 nt]
Synechococcus phage S-PM2 (NC_006820) [196,280 nt]
Synechococcus phage S-H9-2 (NC_070960) [187,320 nt]
Cyanophage S-RIM32 (NC_031235) [194,437 nt]
Prochlorococcus phage Syn1 (NC_015288) [191,195 nt]
Cyanophage S-RIM44 (NC_047734) [197,450 nt]
Synechococcus phage S-WAM2 (NC_031935) [186,386 nt]
Synechococcus phage S-RSM4 (NC_013085) [194,454 nt]
Synechococcus phage S-H9-1 (NC_070961) [192,454 nt]
Synechococcus phage ACG-2014h (NC_023587) [189,311 nt]
Synechococcus phage ACG-2014j (NC_062735) [192,117 nt]
Synechococcus phage S-CAM4 (NC_031900) [191,983 nt]
Synechococcus phage ACG-2014j (NC_026926) [192,108 nt]
Synechococcus phage ACG-2014i (NC_027132) [190,768 nt]
Synechococcus phage ACG-2014e (NC_062736) [189,418 nt]
Synechococcus phage ACG-2014e (NC_026928) [189,418 nt]
Prochlorococcus phage P-HM2 (NC_015284) [183,806 nt]
Prochlorococcus phage P-HM1 (NC_015280) [181,044 nt]
Prochlorococcus phage MED4-213 (NC_020845) [180,977 nt]
Cyanophage P-TIM40 (NC_028663) [188,632 nt]
Cyanophage P-RSM6 (NC_020855) [192,497 nt]
Synechococcus phage S-WAM1 (NC_031944) [185,102 nt]
Synechococcus phage S-SZBM1 (NC_070839) [177,834 nt]
Synechococcus phage S-H38 (NC_070964) [180,224 nt]
Synechococcus phage S-SSM4 (NC_020875) [182,801 nt]
Cyanophage S-SSM6b (HQ316603) [182,368 nt]
Synechococcus phage ACG-2014d (NC_062734) [179,116 nt]
Synechococcus phage ACG-2014d (NC_026923) [179,110 nt]
Synechococcus phage S-ShM2 (NC_015281) [179,563 nt]
Cyanophage S-SSM2 (JF974292) [179,980 nt]
Prochlorococcus phage P-SSM7 (NC_015290) [182,180 nt]
Prochlorococcus phage P-SSM3 (NC_021559) [179,063 nt]
Prochlorococcus phage P-SSM4 (NC_006884) [178,249 nt]
Cyanophage P-RSM3 (HQ634176) [178,750 nt]
Synechococcus phage metaG-MbCM1 (NC_019443) [172,879 nt]
Cyanophage P-RSM1 (NC_021071) [177,211 nt]
Synechococcus phage S-SSM5 (NC_015289) [176,184 nt]
Prochlorococcus phage P-RSM4 (NC_015283) [176,428 nt]
Cyanophage S-TIM4 (NC_048015) [176,495 nt]

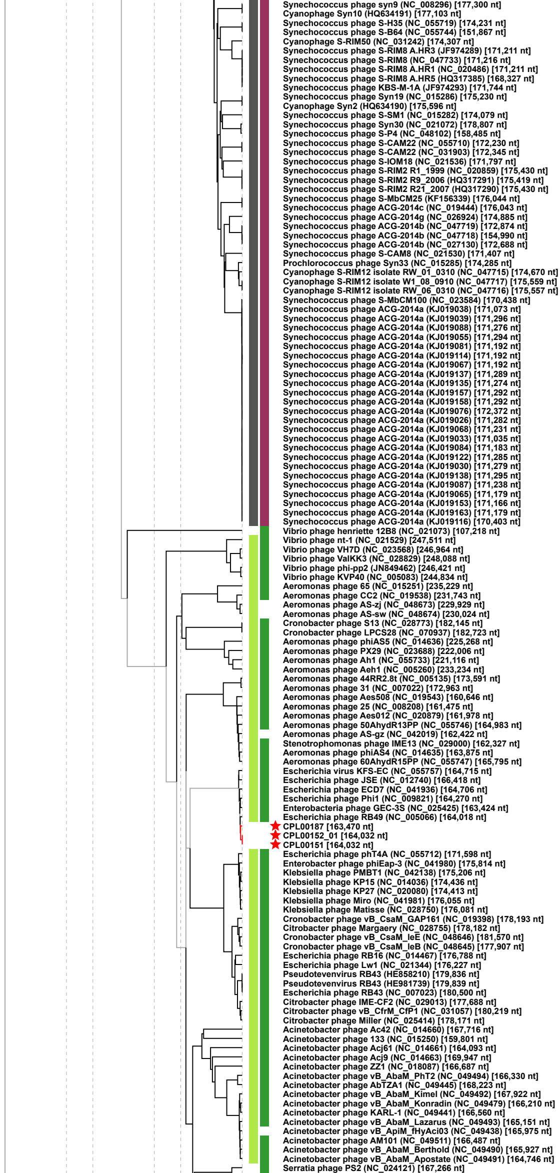

Synechococcus phage syn9 (NC_008296) [177,300 nt]
Cyanophage Syn10 (HQ634191) [177,103 nt]
Synechococcus phage S-H35 (NC_055719) [174,231 nt]
Synechococcus phage S-B64 (NC_055744) [151,867 nt]
Cyanophage S-RIM50 (NC_031242) [174,307 nt]
Synechococcus phage S-RIM8 A.HR3 (JF974289) [171,211 nt]
Synechococcus phage S-RIM8 (NC_047733) [171,216 nt]
Synechococcus phage S-RIM8 A.HR1 (NC_020486) [171,211 nt]
Synechococcus phage S-RIM8 A.HR5 (HQ317385) [168,327 nt]
Synechococcus phage KBS-M-1A (JF974293) [171,744 nt]
Synechococcus phage Syn19 (NC_015286) [175,230 nt]
Cyanophage Syn2 (HQ634190) [175,596 nt]
Synechococcus phage S-SM1 (NC_015282) [174,079 nt]
Synechococcus phage Syn30 (NC_021072) [178,807 nt]
Synechococcus phage S-P4 (NC_048102) [158,485 nt]
Synechococcus phage S-CAM22 (NC_055710) [172,230 nt]
Synechococcus phage S-CAM22 (NC_031903) [172,345 nt]
Synechococcus phage S-IOM18 (NC_021536) [171,797 nt]
Synechococcus phage S-RIM2 R1_1999 (NC_020859) [175,430 nt]
Synechococcus phage S-RIM2 R9_2006 (HQ317291) [175,419 nt]
Synechococcus phage S-RIM2 R21_2007 (HQ317290) [175,430 nt]
Synechococcus phage S-MbCM25 (KF156339) [176,044 nt]
Synechococcus phage ACG-2014c (NC_019444) [176,043 nt]
Synechococcus phage ACG-2014g (NC_026924) [174,885 nt]
Synechococcus phage ACG-2014b (NC_047719) [172,874 nt]
Synechococcus phage ACG-2014b (NC_047718) [154,990 nt]
Synechococcus phage ACG-2014b (NC_027130) [172,688 nt]
Synechococcus phage S-CAM8 (NC_021530) [171,407 nt]
Prochlorococcus phage Syn33 (NC_015285) [174,285 nt]
Cyanophage S-RIM12 isolate RW_01_0310 (NC_047715) [174,670 nt]
Cyanophage S-RIM12 isolate W1_08_0910 (NC_047717) [175,559 nt]
Cyanophage S-RIM12 isolate RW_06_0310 (NC_047716) [175,557 nt]
Synechococcus phage S-MbCM100 (NC_023584) [170,438 nt]
Synechococcus phage ACG-2014a (KJ019038) [171,073 nt]
Synechococcus phage ACG-2014a (KJ019039) [171,296 nt]
Synechococcus phage ACG-2014a (KJ019088) [171,276 nt]
Synechococcus phage ACG-2014a (KJ019055) [171,294 nt]
Synechococcus phage ACG-2014a (KJ019081) [171,192 nt]
Synechococcus phage ACG-2014a (KJ019114) [171,192 nt]
Synechococcus phage ACG-2014a (KJ019067) [171,192 nt]
Synechococcus phage ACG-2014a (KJ019137) [171,289 nt]
Synechococcus phage ACG-2014a (KJ019135) [171,274 nt]
Synechococcus phage ACG-2014a (KJ019157) [171,292 nt]
Synechococcus phage ACG-2014a (KJ019158) [171,292 nt]
Synechococcus phage ACG-2014a (KJ019076) [172,372 nt]
Synechococcus phage ACG-2014a (KJ019026) [171,282 nt]
Synechococcus phage ACG-2014a (KJ019068) [171,231 nt]
Synechococcus phage ACG-2014a (KJ019033) [171,035 nt]
Synechococcus phage ACG-2014a (KJ019084) [171,183 nt]
Synechococcus phage ACG-2014a (KJ019122) [171,285 nt]
Synechococcus phage ACG-2014a (KJ019030) [171,279 nt]
Synechococcus phage ACG-2014a (KJ019138) [171,295 nt]
Synechococcus phage ACG-2014a (KJ019087) [171,238 nt]
Synechococcus phage ACG-2014a (KJ019065) [171,179 nt]
Synechococcus phage ACG-2014a (KJ019153) [171,166 nt]
Synechococcus phage ACG-2014a (KJ019163) [171,179 nt]
Synechococcus phage ACG-2014a (KJ019116) [170,403 nt]
Vibrio phage henriette 12B8 (NC_021073) [107,218 nt]
Vibrio phage nt-1 (NC_021529) [247,511 nt]
Vibrio phage VH7D (NC_023568) [246,964 nt]
Vibrio phage ValKK3 (NC_028829) [248,088 nt]
Vibrio phage phi-pp2 (JN849462) [246,421 nt]
Vibrio phage KVP40 (NC_005083) [244,834 nt]
Aeromonas phage 65 (NC_015251) [235,229 nt]
Aeromonas phage CC2 (NC_019538) [231,743 nt]
Aeromonas phage AS-zj (NC_048673) [229,929 nt]
Aeromonas phage AS-sw (NC_048674) [230,024 nt]
Cronobacter phage S13 (NC_028773) [182,145 nt]
Cronobacter phage LPCS28 (NC_070937) [182,723 nt]
Aeromonas phage phiAS5 (NC_014636) [225,268 nt]
Aeromonas phage PX29 (NC_023688) [222,006 nt]
Aeromonas phage Ah1 (NC_055733) [221,116 nt]
Aeromonas phage Aeh1 (NC_005260) [233,234 nt]
Aeromonas phage 44RR2.8t (NC_005135) [173,591 nt]
Aeromonas phage 31 (NC_007022) [172,963 nt]
Aeromonas phage Aes508 (NC_019543) [160,646 nt]
Aeromonas phage 25 (NC_008208) [161,475 nt]
Aeromonas phage Aes012 (NC_020879) [161,978 nt]
Aeromonas phage 50AhydR13PP (NC_055746) [164,983 nt]
Aeromonas phage AS-gz (NC_042019) [162,422 nt]
Stenotrophomonas phage IME13 (NC_029000) [162,327 nt]
Aeromonas phage phiAS4 (NC_014635) [163,875 nt]
Aeromonas phage 60AhydR15PP (NC_055747) [165,795 nt]
Escherichia virus KFS-EC (NC_055757) [164,715 nt]
Escherichia phage JSE (NC_012740) [166,418 nt]
Escherichia phage ECD7 (NC_041936) [164,706 nt]
Escherichia phage Phi1 (NC_009821) [164,270 nt]
Enterobacteria phage GEC-3S (NC_025425) [163,424 nt]
Escherichia phage RB49 (NC_005066) [164,018 nt]
CPL00187 [163,470 nt]
CPL00152_01 [164,032 nt]
CPL00151 [164,032 nt]
Escherichia phage phT4A (NC_055712) [171,598 nt]
Enterobacter phage phiEap-3 (NC_041980) [175,814 nt]
Klebsiella phage PMBT1 (NC_042138) [175,206 nt]
Klebsiella phage KP15 (NC_014036) [174,436 nt]
Klebsiella phage KP27 (NC_020080) [174,413 nt]
Klebsiella phage Miro (NC_041981) [176,055 nt]
Klebsiella phage Matisse (NC_028750) [176,081 nt]
Cronobacter phage vB_CsaM_GAP161 (NC_019398) [178,193 nt]
Citrobacter phage Margaery (NC_028755) [178,182 nt]
Cronobacter phage vB_CsaM_leE (NC_048646) [181,570 nt]
Cronobacter phage vB_CsaM_leB (NC_048645) [177,907 nt]
Escherichia phage RB16 (NC_014467) [176,788 nt]
Escherichia phage Lw1 (NC_021344) [176,227 nt]
Pseudotevenvirus RB43 (HE858210) [179,836 nt]
Pseudotevenvirus RB43 (HE981739) [179,839 nt]
Escherichia phage RB43 (NC_007023) [180,500 nt]
Citrobacter phage IME-CF2 (NC_029013) [177,688 nt]
Citrobacter phage vB_CfrM_CfP1 (NC_031057) [180,219 nt]
Citrobacter phage Miller (NC_025414) [178,171 nt]
Acinetobacter phage Ac42 (NC_014660) [167,716 nt]
Acinetobacter phage 133 (NC_015250) [159,801 nt]
Acinetobacter phage Acj61 (NC_014661) [164,093 nt]
Acinetobacter phage Acj9 (NC_014663) [169,947 nt]
Acinetobacter phage ZZ1 (NC_018087) [166,687 nt]
Acinetobacter phage vB_AbaM_PhT2 (NC_049494) [166,330 nt]
Acinetobacter phage AbTZA1 (NC_049445) [168,223 nt]
Acinetobacter phage vB_AbaM_Kimel (NC_049492) [167,922 nt]
Acinetobacter phage vB_AbaM_Konradin (NC_049479) [166,210 nt]
Acinetobacter phage KARL-1 (NC_049441) [166,560 nt]
Acinetobacter phage vB_AbaM_Lazarus (NC_049493) [165,151 nt]
Acinetobacter phage vB_ApiM_fHyAci03 (NC_049438) [165,975 nt]
Acinetobacter phage AM101 (NC_049511) [166,487 nt]
Acinetobacter phage vB_AbaM_Berthold (NC_049490) [165,927 nt]
Acinetobacter phage vB_AbaM_Apostate (NC_049491) [164,746 nt]
Serratia phage PS2 (NC_024121) [167,266 nt]

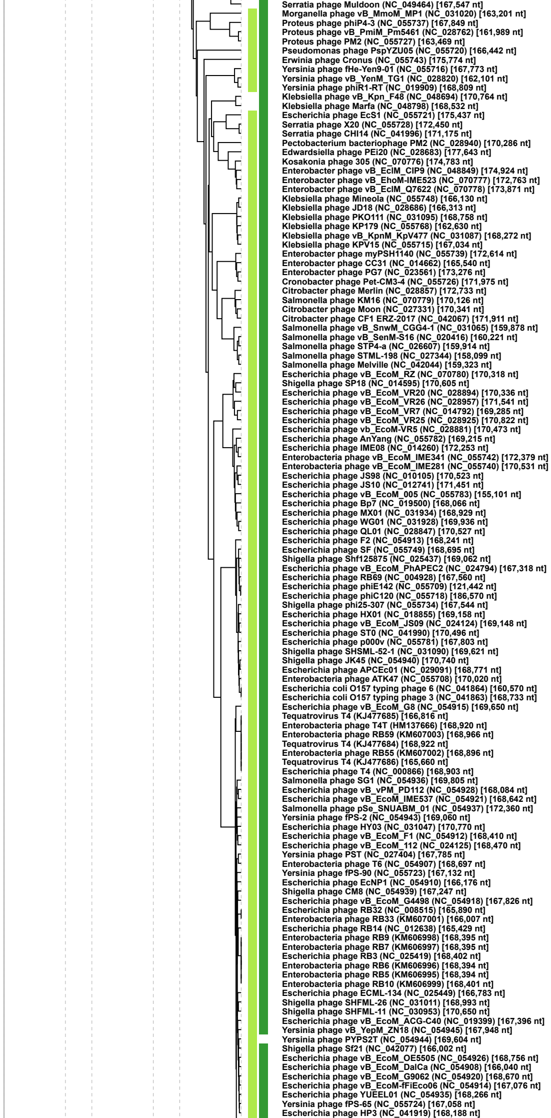
Serratia phage Muldoon (NC_049464) [167,547 nt]
Morganella phage vB_MmoM_MP1 (NC_031020) [163,201 nt]
Proteus phage phiP4-3 (NC_055737) [167,849 nt]
Proteus phage vB_PmiM_Pm5461 (NC_028762) [161,989 nt]
Proteus phage PM2 (NC_055727) [163,469 nt]
Pseudomonas phage PspYZU05 (NC_055720) [166,442 nt]
Erwinia phage Cronus (NC_055743) [175,774 nt]
Yersinia phage fHe-Yen9-01 (NC_055716) [167,773 nt]
Yersinia phage vB_YenM_TG1 (NC_028820) [162,101 nt]
Yersinia phage phiR1-RT (NC_019909) [168,809 nt]
Klebsiella phage vB_Kpn_F48 (NC_048694) [170,764 nt]
Klebsiella phage Marfa (NC_048798) [168,532 nt]
Escherichia phage EcS1 (NC_055721) [175,437 nt]
Serratia phage X20 (NC_055728) [172,450 nt]
Serratia phage CHI14 (NC_041996) [171,175 nt]
Pectobacterium bacteriophage PM2 (NC_028940) [170,286 nt]
Edwardsiella phage PEi20 (NC_028683) [177,643 nt]
Kosakonia phage 305 (NC_070776) [174,783 nt]
Enterobacter phage vB_EclM_CIP9 (NC_048849) [174,924 nt]
Enterobacter phage vB_EhoM-IME523 (NC_070777) [172,763 nt]
Enterobacter phage vB_EclM_Q7622 (NC_070778) [173,871 nt]
Klebsiella phage Mineola (NC_055748) [166,130 nt]
Klebsiella phage JD18 (NC_028686) [166,313 nt]
Klebsiella phage PKO111 (NC_031095) [168,758 nt]
Klebsiella phage KP179 (NC_055768) [162,630 nt]
Klebsiella phage vB_KpnM_KpV477 (NC_031087) [168,272 nt]
Klebsiella phage KPV15 (NC_055715) [167,034 nt]
Enterobacter phage myPSH1140 (NC_055739) [172,614 nt]
Enterobacter phage CC31 (NC_014662) [165,540 nt]
Enterobacter phage PG7 (NC_023561) [173,276 nt]
Cronobacter phage Pet-CM3-4 (NC_055726) [171,975 nt]
Citrobacter phage Merlin (NC_028857) [172,733 nt]
Salmonella phage KM16 (NC_070779) [170,126 nt]
Citrobacter phage Moon (NC_027331) [170,341 nt]
Citrobacter phage CF1 ERZ-2017 (NC_042067) [171,911 nt]
Salmonella phage vB_SnwM_CGG4-1 (NC_031065) [159,878 nt]
Salmonella phage vB_SenM-S16 (NC_020416) [160,221 nt]
Salmonella phage STP4-a (NC_026607) [159,914 nt]
Salmonella phage STML-198 (NC_027344) [158,099 nt]
Salmonella phage Melville (NC_042044) [159,323 nt]
Escherichia phage vB_EcoM_RZ (NC_070780) [170,318 nt]
Shigella phage SP18 (NC_014595) [170,605 nt]
Escherichia phage vB_EcoM_VR20 (NC_028894) [170,336 nt]
Escherichia phage vB_EcoM_VR26 (NC_028957) [171,541 nt]
Escherichia phage vB_EcoM_VR7 (NC_014792) [169,285 nt]
Escherichia phage vB_EcoM_VR25 (NC_028925) [170,822 nt]
Escherichia phage vb_EcoM-VR5 (NC_028881) [170,473 nt]
Escherichia phage AnYang (NC_055782) [169,215 nt]
Escherichia phage IME08 (NC_014260) [172,253 nt]
Enterobacteria phage vB_EcoM_IME341 (NC_055742) [172,379 nt]
Enterobacteria phage vB_EcoM_IME281 (NC_055740) [170,531 nt]
Escherichia phage JS98 (NC_010105) [170,523 nt]
Escherichia phage JS10 (NC_012741) [171,451 nt]
Escherichia phage vB_EcoM_005 (NC_055783) [155,101 nt]
Escherichia phage Bp7 (NC_019500) [168,066 nt]
Escherichia phage MX01 (NC_031934) [168,929 nt]
Escherichia phage WG01 (NC_031928) [169,936 nt]
Escherichia phage QL01 (NC_028847) [170,527 nt]
Escherichia phage F2 (NC_054913) [168,241 nt]
Escherichia phage SF (NC_055749) [168,695 nt]
Shigella phage Shf125875 (NC_025437) [169,062 nt]
Escherichia phage vB_EcoM_PhAPEC2 (NC_024794) [167,318 nt]
Escherichia phage RB69 (NC_004928) [167,560 nt]
Escherichia phage phiE142 (NC_055709) [121,442 nt]
Escherichia phage phiC120 (NC_055718) [186,570 nt]
Shigella phage phi25-307 (NC_055734) [167,544 nt]
Escherichia phage HX01 (NC_018855) [169,158 nt]
Escherichia phage vB_EcoM_JS09 (NC_024124) [169,148 nt]
Escherichia phage ST0 (NC_041990) [170,496 nt]
Escherichia phage p000v (NC_055781) [167,803 nt]
Shigella phage SHSML-52-1 (NC_031090) [169,621 nt]
Shigella phage JK45 (NC_054940) [170,740 nt]
Escherichia phage APCEc01 (NC_029091) [168,771 nt]
Enterobacteria phage ATK47 (NC_055708) [170,020 nt]
Escherichia coli O157 typing phage 6 (NC_041864) [160,570 nt]
Escherichia coli O157 typing phage 3 (NC_041863) [168,733 nt]
Escherichia phage vB_EcoM_G8 (NC_054915) [169,650 nt]
Tequatrovirus T4 (KJ477685) [166,816 nt]
Enterobacteria phage T4T (HM137666) [168,920 nt]
Enterobacteria phage RB59 (KM607003) [168,966 nt]
Tequatrovirus T4 (KJ477684) [168,922 nt]
Enterobacteria phage RB55 (KM607002) [168,896 nt]
Tequatrovirus T4 (KJ477686) [165,660 nt]
Escherichia phage T4 (NC_000866) [168,903 nt]
Salmonella phage SG1 (NC_054936) [169,805 nt]
Escherichia phage vB_vPM_PD112 (NC_054928) [168,084 nt]
Escherichia phage vB_EcoM_IME537 (NC_054921) [168,642 nt]
Salmonella phage pSe_SNUABM_01 (NC_054937) [172,360 nt]
Yersinia phage fPS-2 (NC_054943) [169,060 nt]
Escherichia phage HY03 (NC_031047) [170,770 nt]
Escherichia phage vB_EcoM_F1 (NC_054912) [168,410 nt]
Escherichia phage vB_EcoM_112 (NC_024125) [168,470 nt]
Yersinia phage PST (NC_027404) [167,785 nt]
Enterobacteria phage T6 (NC_054907) [168,697 nt]
Yersinia phage fPS-90 (NC_055723) [167,132 nt]
Escherichia phage EcNP1 (NC_054910) [166,176 nt]
Shigella phage CM8 (NC_054939) [167,247 nt]
Escherichia phage vB_EcoM_G4498 (NC_054918) [167,826 nt]
Escherichia phage RB32 (NC_008515) [165,890 nt]
Enterobacteria phage RB33 (KM607001) [166,007 nt]
Escherichia phage RB14 (NC_012638) [165,429 nt]
Enterobacteria phage RB9 (KM606998) [168,395 nt]
Enterobacteria phage RB7 (KM606997) [168,395 nt]
Escherichia phage RB3 (NC_025419) [168,402 nt]
Enterobacteria phage RB6 (KM606996) [168,394 nt]
Enterobacteria phage RB5 (KM606995) [168,394 nt]
Enterobacteria phage RB10 (KM606999) [168,401 nt]
Escherichia phage ECML-134 (NC_025449) [166,783 nt]
Shigella phage SHFML-26 (NC_031011) [168,993 nt]
Shigella phage SHFML-11 (NC_030953) [170,650 nt]
Escherichia phage vB_EcoM_ACG-C40 (NC_019399) [167,396 nt]
Yersinia phage vB_YepM_ZN18 (NC_054945) [167,948 nt]
Yersinia phage PYPS2T (NC_054944) [169,604 nt]
Shigella phage Sf21 (NC_042077) [166,002 nt]
Escherichia phage vB_EcoM_OE5505 (NC_054926) [168,756 nt]
Escherichia phage vB_EcoM_DalCa (NC_054908) [166,040 nt]
Escherichia phage vB_EcoM_G9062 (NC_054920) [168,670 nt]
Escherichia phage vB_EcoM-fFiEco06 (NC_054914) [167,076 nt]
Escherichia phage YUEEL01 (NC_054935) [168,266 nt]
Yersinia phage fPS-65 (NC_055724) [167,058 nt]
Escherichia phage HP3 (NC_041919) [168,188 nt]

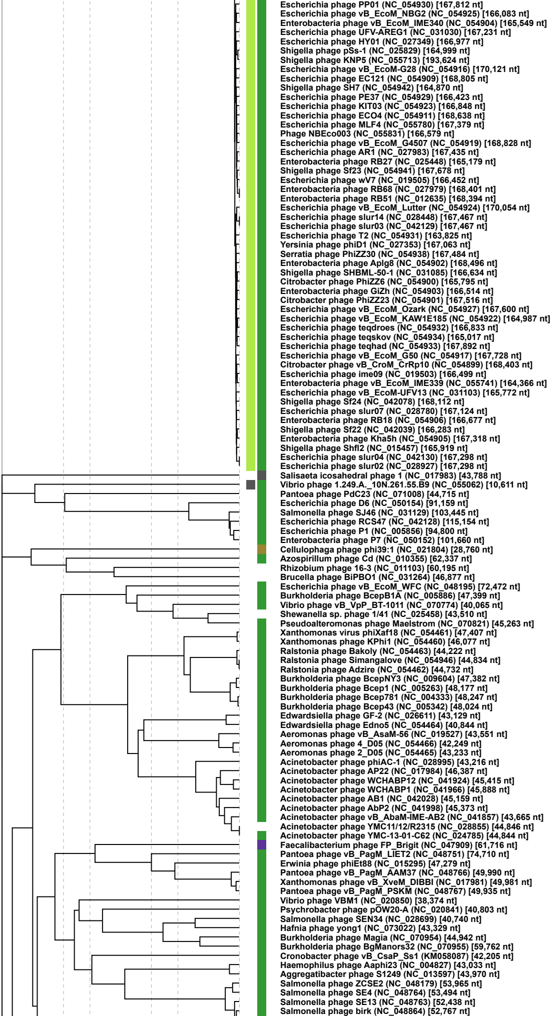

Escherichia phage PP01 (NC_054930) [167,812 nt]
Escherichia phage vB_EcoM_NBG2 (NC_054925) [166,083 nt]
Enterobacteria phage vB_EcoM_IME340 (NC_054904) [165,549 nt]
Escherichia phage UFV-AREG1 (NC_031030) [167,231 nt]
Escherichia phage HY01 (NC_027349) [166,977 nt]
Shigella phage pSs-1 (NC_025829) [164,999 nt]
Shigella phage KNP5 (NC_055713) [193,624 nt]
Escherichia phage vB_EcoM-G28 (NC_054916) [170,121 nt]
Escherichia phage EC121 (NC_054909) [168,805 nt]
Shigella phage SH7 (NC_054942) [164,870 nt]
Escherichia phage PE37 (NC_054929) [166,423 nt]
Escherichia phage KIT03 (NC_054923) [166,848 nt]
Escherichia phage ECO4 (NC_054911) [168,638 nt]
Escherichia phage MLF4 (NC_055780) [167,379 nt]
Phage NBEco003 (NC_055831) [166,579 nt]
Escherichia phage vB_EcoM_G4507 (NC_054919) [168,828 nt]
Escherichia phage AR1 (NC_027983) [167,435 nt]
Enterobacteria phage RB27 (NC_025448) [165,179 nt]
Shigella phage Sf23 (NC_054941) [167,678 nt]
Escherichia phage wV7 (NC_019505) [166,452 nt]
Enterobacteria phage RB68 (NC_027979) [168,401 nt]
Enterobacteria phage RB51 (NC_012635) [168,394 nt]
Escherichia phage vB_EcoM_Lutter (NC_054924) [170,054 nt]
Escherichia phage slur14 (NC_028448) [167,467 nt]
Escherichia phage slur03 (NC_042129) [167,467 nt]
Escherichia phage T2 (NC_054931) [163,825 nt]
Yersinia phage phiD1 (NC_027353) [167,063 nt]
Serratia phage PhiZZ30 (NC_054938) [167,484 nt]
Enterobacteria phage Aplg8 (NC_054902) [168,496 nt]
Shigella phage SHBML-50-1 (NC_031085) [166,634 nt]
Citrobacter phage PhiZZ6 (NC_054900) [165,795 nt]
Enterobacteria phage GiZh (NC_054903) [166,514 nt]
Citrobacter phage PhiZZ23 (NC_054901) [167,516 nt]
Escherichia phage vB_EcoM_Ozark (NC_054927) [167,600 nt]
Escherichia phage vB_EcoM_KAW1E185 (NC_054922) [164,987 nt]
Escherichia phage teqdroes (NC_054932) [166,833 nt]
Escherichia phage teqskov (NC_054934) [165,017 nt]
Escherichia phage teqhad (NC_054933) [167,892 nt]
Escherichia phage vB_EcoM_G50 (NC_054917) [167,728 nt]
Citrobacter phage vB_CroM_CrRp10 (NC_054899) [168,403 nt]
Escherichia phage ime09 (NC_019503) [166,499 nt]
Enterobacteria phage vB_EcoM_IME339 (NC_055741) [164,366 nt]
Escherichia phage vB_EcoM-UFV13 (NC_031103) [165,772 nt]
Shigella phage Sf24 (NC_042078) [168,112 nt]
Escherichia phage slur07 (NC_028780) [167,124 nt]
Enterobacteria phage RB18 (NC_054906) [166,677 nt]
Shigella phage Sf22 (NC_042039) [166,283 nt]
Enterobacteria phage Kha5h (NC_054905) [167,318 nt]
Shigella phage Shfl2 (NC_015457) [165,919 nt]
Escherichia phage slur04 (NC_042130) [167,298 nt]
Escherichia phage slur02 (NC_028927) [167,298 nt]
Salisaeta icosahedral phage 1 (NC_017983) [43,788 nt]
Vibrio phage 1.249.A._10N.261.55.B9 (NC_055062) [10,611 nt]
Pantoea phage PdC23 (NC_071008) [44,715 nt]
Escherichia phage D6 (NC_050154) [91,159 nt]
Salmonella phage SJ46 (NC_031129) [103,445 nt]
Escherichia phage RCS47 (NC_042128) [115,154 nt]
Escherichia phage P1 (NC_005856) [94,800 nt]
Enterobacteria phage P7 (NC_050152) [101,660 nt]
Cellulophaga phage phi39:1 (NC_021804) [28,760 nt]
Azospirillum phage Cd (NC_010355) [62,337 nt]
Rhizobium phage 16-3 (NC_011103) [60,195 nt]
Brucella phage BiPBO1 (NC_031264) [46,877 nt]
Escherichia phage vB_EcoM_WFC (NC_048195) [72,472 nt]
Burkholderia phage BcepB1A (NC_005886) [47,399 nt]
Vibrio phage vB_VpP_BT-1011 (NC_070774) [40,065 nt]
Shewanella sp. phage 1/41 (NC_025458) [43,510 nt]
Pseudoalteromonas phage Maelstrom (NC_070821) [45,263 nt]
Xanthomonas virus phiXaf18 (NC_054461) [47,407 nt]
Xanthomonas phage KPhi1 (NC_054460) [46,077 nt]
Ralstonia phage Bakoly (NC_054463) [44,222 nt]
Ralstonia phage Simangalove (NC_054946) [44,834 nt]
Ralstonia phage Adzire (NC_054462) [44,732 nt]
Burkholderia phage BcepNY3 (NC_009604) [47,382 nt]
Burkholderia phage Bcep1 (NC_005263) [48,177 nt]
Burkholderia phage Bcep781 (NC_004333) [48,247 nt]
Burkholderia phage Bcep43 (NC_005342) [48,024 nt]
Edwardsiella phage GF-2 (NC_026611) [43,129 nt]
Edwardsiella phage Edno5 (NC_054464) [40,844 nt]
Aeromonas phage vB_AsaM-56 (NC_019527) [43,551 nt]
Aeromonas phage 4_D05 (NC_054466) [42,249 nt]
Aeromonas phage 2_D05 (NC_054465) [43,233 nt]
Acinetobacter phage phiAC-1 (NC_028995) [43,216 nt]
Acinetobacter phage AP22 (NC_017984) [46,387 nt]
Acinetobacter phage WCHABP12 (NC_041924) [45,415 nt]
Acinetobacter phage WCHABP1 (NC_041966) [45,888 nt]
Acinetobacter phage AB1 (NC_042028) [45,159 nt]
Acinetobacter phage AbP2 (NC_041998) [45,373 nt]
Acinetobacter phage vB_AbaM-IME-AB2 (NC_041857) [43,665 nt]
Acinetobacter phage YMC11/12/R2315 (NC_028855) [44,846 nt]
Acinetobacter phage YMC-13-01-C62 (NC_024785) [44,844 nt]
Faecalibacterium phage FP_Brigit (NC_047909) [61,716 nt]
Pantoea phage vB_PagM_LIET2 (NC_048751) [74,710 nt]
Erwinia phage phiEt88 (NC_015295) [47,279 nt]
Pantoea phage vB_PagM_AAM37 (NC_048766) [49,990 nt]
Xanthomonas phage vB_XveM_DIBBI (NC_017981) [49,981 nt]
Pantoea phage vB_PagM_PSKM (NC_048767) [49,935 nt]
Vibrio phage VBM1 (NC_020850) [38,374 nt]
Psychrobacter phage pOW20-A (NC_020841) [40,803 nt]
Salmonella phage SEN34 (NC_028699) [40,740 nt]
Hafnia phage yong1 (NC_073022) [43,329 nt]
Burkholderia phage Magia (NC_070954) [44,942 nt]
Burkholderia phage BgManors32 (NC_070955) [59,762 nt]
Cronobacter phage vB_CsaP_Ss1 (KM058087) [42,205 nt]
Haemophilus phage Aaphi23 (NC_004827) [43,033 nt]
Aggregatibacter phage S1249 (NC_013597) [43,970 nt]
Salmonella phage ZCSE2 (NC_048179) [53,965 nt]
Salmonella phage SE4 (NC_048764) [53,494 nt]
Salmonella phage SE13 (NC_048763) [52,438 nt]
Salmonella phage birk (NC_048864) [52,767 nt]

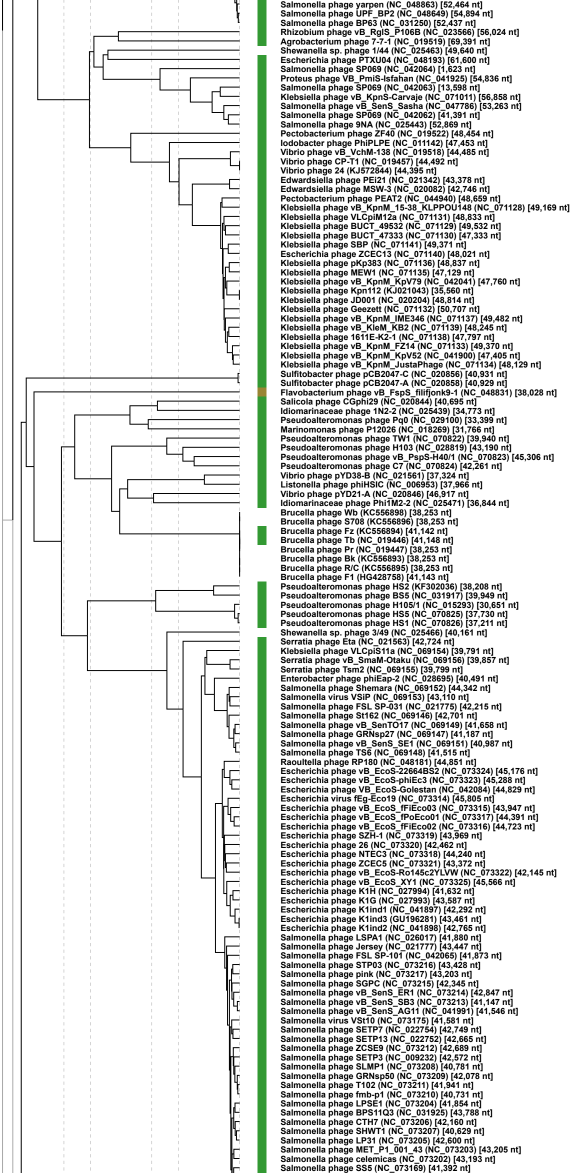

Salmonella phage yarpen (NC_048863) [52,464 nt]
Salmonella phage UPF_BP2 (NC_048649) [54,894 nt]
Salmonella phage BP63 (NC_031250) [52,437 nt]
Rhizobium phage vB_RglS_P106B (NC_023566) [56,024 nt]
Agrobacterium phage 7-7-1 (NC_019519) [69,391 nt]
Shewanella sp. phage 1/44 (NC_025463) [49,640 nt]
Escherichia phage PTXU04 (NC_048193) [61,600 nt]
Salmonella phage SP069 (NC_042064) [1,623 nt]
Proteus phage VB_PmiS-Isfahan (NC_041925) [54,836 nt]
Salmonella phage SP069 (NC_042063) [13,598 nt]
Klebsiella phage vB_KpnS-Carvaje (NC_071011) [56,858 nt]
Salmonella phage vB_SenS_Sasha (NC_047786) [53,263 nt]
Salmonella phage SP069 (NC_042062) [41,391 nt]
Salmonella phage 9NA (NC_025443) [52,869 nt]
Pectobacterium phage ZF40 (NC_019522) [48,454 nt]
Iodobacter phage PhiPLPE (NC_011142) [47,453 nt]
Vibrio phage vB_VchM-138 (NC_019518) [44,485 nt]
Vibrio phage CP-T1 (NC_019457) [44,492 nt]
Vibrio phage 24 (KJ572844) [44,395 nt]
Edwardsiella phage PEi21 (NC_021342) [43,378 nt]
Edwardsiella phage MSW-3 (NC_020082) [42,746 nt]
Pectobacterium phage PEAT2 (NC_044940) [48,659 nt]
Klebsiella phage vB_KpnM_15-38_KLPPOU148 (NC_071128) [49,169 nt]
Klebsiella phage VLCpiM12a (NC_071131) [48,833 nt]
Klebsiella phage BUCT_49532 (NC_071129) [49,532 nt]
Klebsiella phage BUCT_47333 (NC_071130) [47,333 nt]
Klebsiella phage SBP (NC_071141) [49,371 nt]
Escherichia phage ZCEC13 (NC_071140) [48,021 nt]
Klebsiella phage pKp383 (NC_071136) [48,837 nt]
Klebsiella phage MEW1 (NC_071135) [47,129 nt]
Klebsiella phage vB_KpnM_KpV79 (NC_042041) [47,760 nt]
Klebsiella phage Kpn112 (KJ021043) [35,560 nt]
Klebsiella phage JD001 (NC_020204) [48,814 nt]
Klebsiella phage Geezett (NC_071132) [50,707 nt]
Klebsiella phage vB_KpnM_IME346 (NC_071137) [49,482 nt]
Klebsiella phage vB_KleM_KB2 (NC_071139) [48,245 nt]
Klebsiella phage 1611E-K2-1 (NC_071138) [47,797 nt]
Klebsiella phage vB_KpnM_FZ14 (NC_071133) [49,370 nt]
Klebsiella phage vB_KpnM_KpV52 (NC_041900) [47,405 nt]
Klebsiella phage vB_KpnM_JustaPhage (NC_071134) [48,129 nt]
Sulfitobacter phage pCB2047-C (NC_020856) [40,931 nt]
Sulfitobacter phage pCB2047-A (NC_020858) [40,929 nt]
Flavobacterium phage vB_FspS_filifjonk9-1 (NC_048831) [38,028 nt]
Salicola phage CGphi29 (NC_020844) [40,695 nt]
Idiomarinaceae phage 1N2-2 (NC_025439) [34,773 nt]
Pseudoalteromonas phage Pq0 (NC_029100) [33,399 nt]
Marinomonas phage P12026 (NC_018269) [31,766 nt]
Pseudoalteromonas phage TW1 (NC_070822) [39,940 nt]
Pseudoalteromonas phage H103 (NC_028819) [43,190 nt]
Pseudoalteromonas phage vB_PspS-H40/1 (NC_070823) [45,306 nt]
Pseudoalteromonas phage C7 (NC_070824) [42,261 nt]
Vibrio phage pYD38-B (NC_021561) [37,324 nt]
Listonella phage phiHSIC (NC_006953) [37,966 nt]
Vibrio phage pYD21-A (NC_020846) [46,917 nt]
Idiomarinaceae phage Phi1M2-2 (NC_025471) [36,844 nt]
Brucella phage Wb (KC556898) [38,253 nt]
Brucella phage S708 (KC556896) [38,253 nt]
Brucella phage Fz (KC556894) [41,142 nt]
Brucella phage Tb (NC_019446) [41,148 nt]
Brucella phage Pr (NC_019447) [38,253 nt]
Brucella phage Bk (KC556893) [38,253 nt]
Brucella phage R/C (KC556895) [38,253 nt]
Brucella phage F1 (HG428758) [41,143 nt]
Pseudoalteromonas phage HS2 (KF302036) [38,208 nt]
Pseudoalteromonas phage BS5 (NC_031917) [39,949 nt]
Pseudoalteromonas phage H105/1 (NC_015293) [30,651 nt]
Pseudoalteromonas phage HS5 (NC_070825) [37,730 nt]
Pseudoalteromonas phage HS1 (NC_070826) [37,211 nt]
Shewanella sp. phage 3/49 (NC_025466) [40,161 nt]
Serratia phage Eta (NC_021563) [42,724 nt]
Klebsiella phage VLCpiS11a (NC_069154) [39,791 nt]
Serratia phage vB_SmaM-Otaku (NC_069156) [39,857 nt]
Serratia phage Tsm2 (NC_069155) [39,799 nt]
Enterobacter phage phiEap-2 (NC_028695) [40,491 nt]
Salmonella phage Shemara (NC_069152) [44,342 nt]
Salmonella virus VSiP (NC_069153) [43,110 nt]
Salmonella phage FSL SP-031 (NC_021775) [42,215 nt]
Salmonella phage St162 (NC_069146) [42,701 nt]
Salmonella phage vB_SenTO17 (NC_069149) [41,658 nt]
Salmonella phage GRNsp27 (NC_069147) [41,187 nt]
Salmonella phage vB_SenS_SE1 (NC_069151) [40,987 nt]
Salmonella phage TS6 (NC_069148) [41,515 nt]
Raoultella phage RP180 (NC_048181) [44,851 nt]
Escherichia phage vB_EcoS-22664BS2 (NC_073324) [45,176 nt]
Escherichia phage vB_EcoS-phiEc3 (NC_073323) [45,288 nt]
Escherichia phage VB_EcoS-Golestan (NC_042084) [44,829 nt]
Escherichia virus fEg-Eco19 (NC_073314) [45,805 nt]
Escherichia phage vB_EcoS_fFiEco03 (NC_073315) [43,947 nt]
Escherichia phage vB_EcoS_fPoEco01 (NC_073317) [44,391 nt]
Escherichia phage vB_EcoS_fFiEco02 (NC_073316) [44,723 nt]
Escherichia phage SZH-1 (NC_073319) [43,969 nt]
Escherichia phage 26 (NC_073320) [42,462 nt]
Escherichia phage NTEC3 (NC_073318) [44,240 nt]
Escherichia phage ZCEC5 (NC_073321) [43,372 nt]
Escherichia phage vB_EcoS-Ro145c2YLVW (NC_073322) [42,145 nt]
Escherichia phage vB_EcoS_XY1 (NC_073325) [45,566 nt]
Escherichia phage K1H (NC_027994) [41,632 nt]
Escherichia phage K1G (NC_027993) [43,587 nt]
Escherichia phage K1ind1 (NC_041897) [42,292 nt]
Escherichia phage K1ind3 (GU196281) [43,461 nt]
Escherichia phage K1ind2 (NC_041898) [42,765 nt]
Salmonella phage LSPA1 (NC_026017) [41,880 nt]
Salmonella phage Jersey (NC_021777) [43,447 nt]
Salmonella phage FSL SP-101 (NC_042065) [41,873 nt]
Salmonella phage STP03 (NC_073216) [43,428 nt]
Salmonella phage pink (NC_073217) [43,203 nt]
Salmonella phage SGPC (NC_073215) [42,345 nt]
Salmonella phage vB_SenS_ER1 (NC_073214) [42,847 nt]
Salmonella phage vB_SenS_SB3 (NC_073213) [41,147 nt]
Salmonella phage vB_SenS_AG11 (NC_041991) [41,546 nt]
Salmonella virus VSt10 (NC_073175) [41,581 nt]
Salmonella phage SETP7 (NC_022754) [42,749 nt]
Salmonella phage SETP13 (NC_022752) [42,665 nt]
Salmonella phage ZCSE9 (NC_073212) [42,689 nt]
Salmonella phage SETP3 (NC_009232) [42,572 nt]
Salmonella phage SLMP1 (NC_073208) [40,781 nt]
Salmonella phage GRNsp50 (NC_073209) [42,078 nt]
Salmonella phage T102 (NC_073211) [41,941 nt]
Salmonella phage fmb-p1 (NC_073210) [40,731 nt]
Salmonella phage LPSE1 (NC_073204) [41,854 nt]
Salmonella phage BPS11Q3 (NC_031925) [43,788 nt]
Salmonella phage CTH7 (NC_073206) [42,160 nt]
Salmonella phage SHWT1 (NC_073207) [40,629 nt]
Salmonella phage LP31 (NC_073205) [42,600 nt]
Salmonella phage MET_P1_001_43 (NC_073203) [43,205 nt]
Salmonella phage celemicas (NC_073202) [43,193 nt]
Salmonella phage SS5 (NC_073169) [41,392 nt]

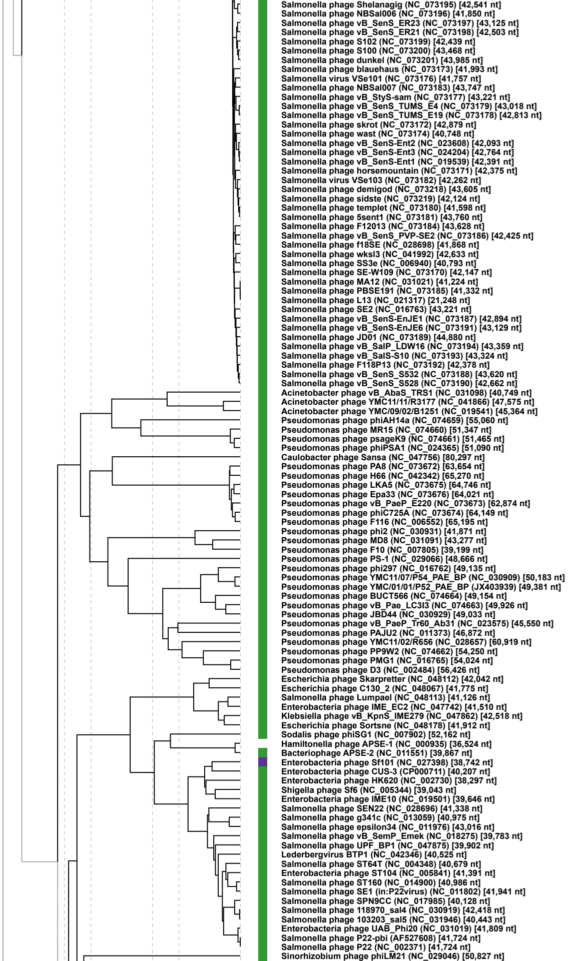

Salmonella phage Shelanagig (NC_073195) [42,541 nt]
Salmonella phage NBSal006 (NC_073196) [41,850 nt]
Salmonella phage vB_SenS_ER23 (NC_073197) [43,125 nt]
Salmonella phage vB_SenS_ER21 (NC_073198) [42,503 nt]
Salmonella phage S102 (NC_073199) [42,439 nt]
Salmonella phage S100 (NC_073200) [43,468 nt]
Salmonella phage dunkel (NC_073201) [43,985 nt]
Salmonella phage blauehaus (NC_073173) [41,993 nt]
Salmonella virus VSe101 (NC_073176) [41,757 nt]
Salmonella phage NBSal007 (NC_073183) [43,747 nt]
Salmonella phage vB_StyS-sam (NC_073177) [43,221 nt]
Salmonella phage vB_SenS_TUMS_E4 (NC_073179) [43,018 nt]
Salmonella phage vB_SenS_TUMS_E19 (NC_073178) [42,813 nt]
Salmonella phage skrot (NC_073172) [42,879 nt]
Salmonella phage wast (NC_073174) [40,748 nt]
Salmonella phage vB_SenS-Ent2 (NC_023608) [42,093 nt]
Salmonella phage vB_SenS-Ent3 (NC_024204) [42,764 nt]
Salmonella phage vB_SenS-Ent1 (NC_019539) [42,391 nt]
Salmonella phage horsemountain (NC_073171) [42,375 nt]
Salmonella virus VSe103 (NC_073182) [42,262 nt]
Salmonella phage demigod (NC_073218) [43,605 nt]
Salmonella phage sidste (NC_073219) [42,124 nt]
Salmonella phage templet (NC_073180) [41,598 nt]
Salmonella phage 5sent1 (NC_073181) [43,760 nt]
Salmonella phage F12013 (NC_073184) [43,628 nt]
Salmonella phage vB_SenS_PVP-SE2 (NC_073186) [42,425 nt]
Salmonella phage f18SE (NC_028698) [41,868 nt]
Salmonella phage wksl3 (NC_041992) [42,633 nt]
Salmonella phage SS3e (NC_006940) [40,793 nt]
Salmonella phage SE-W109 (NC_073170) [42,147 nt]
Salmonella phage MA12 (NC_031021) [41,224 nt]
Salmonella phage PBSE191 (NC_073185) [41,332 nt]
Salmonella phage L13 (NC_021317) [21,248 nt]
Salmonella phage SE2 (NC_016763) [43,221 nt]
Salmonella phage vB_SenS-EnJE1 (NC_073187) [42,894 nt]
Salmonella phage vB_SenS-EnJE6 (NC_073191) [43,129 nt]
Salmonella phage JD01 (NC_073189) [44,880 nt]
Salmonella phage vB_SalP_LDW16 (NC_073194) [43,359 nt]
Salmonella phage vB_SalS-S10 (NC_073193) [43,324 nt]
Salmonella phage F118P13 (NC_073192) [42,378 nt]
Salmonella phage vB_SenS_S532 (NC_073188) [43,620 nt]
Salmonella phage vB_SenS_S528 (NC_073190) [42,662 nt]
Acinetobacter phage vB_AbaS_TRS1 (NC_031098) [40,749 nt]
Acinetobacter phage YMC11/11/R3177 (NC_041866) [47,575 nt]
Acinetobacter phage YMC/09/02/B1251 (NC_019541) [45,364 nt]
Pseudomonas phage phiAH14a (NC_074659) [55,060 nt]
Pseudomonas phage MR15 (NC_074660) [51,347 nt]
Pseudomonas phage psageK9 (NC_074661) [51,465 nt]
Pseudomonas phage phiPSA1 (NC_024365) [51,090 nt]
Caulobacter phage Sansa (NC_047756) [80,297 nt]
Pseudomonas phage PA8 (NC_073672) [63,654 nt]
Pseudomonas phage H66 (NC_042342) [65,270 nt]
Pseudomonas phage LKA5 (NC_073675) [64,746 nt]
Pseudomonas phage Epa33 (NC_073676) [64,021 nt]
Pseudomonas phage vB_PaeP_E220 (NC_073673) [62,874 nt]
Pseudomonas phage phiC725A (NC_073674) [64,149 nt]
Pseudomonas phage F116 (NC_006552) [65,195 nt]
Pseudomonas phage phi2 (NC_030931) [41,871 nt]
Pseudomonas phage MD8 (NC_031091) [43,277 nt]
Pseudomonas phage F10 (NC_007805) [39,199 nt]
Pseudomonas phage PS-1 (NC_029066) [48,666 nt]
Pseudomonas phage phi297 (NC_016762) [49,135 nt]
Pseudomonas phage YMC11/07/P54_PAE_BP (NC_030909) [50,183 nt]
Pseudomonas phage YMC/01/01/P52_PAE_BP (JX403939) [49,381 nt]
Pseudomonas phage BUCT566 (NC_074664) [49,154 nt]
Pseudomonas phage vB_Pae_LC3I3 (NC_074663) [49,926 nt]
Pseudomonas phage JBD44 (NC_030929) [49,033 nt]
Pseudomonas phage vB_PaeP_Tr60_Ab31 (NC_023575) [45,550 nt]
Pseudomonas phage PAJU2 (NC_011373) [46,872 nt]
Pseudomonas phage YMC11/02/R656 (NC_028657) [60,919 nt]
Pseudomonas phage PP9W2 (NC_074662) [54,250 nt]
Pseudomonas phage PMG1 (NC_016765) [54,024 nt]
Pseudomonas phage D3 (NC_002484) [56,426 nt]
Escherichia phage Skarpretter (NC_048112) [42,042 nt]
Escherichia phage C130_2 (NC_048067) [41,775 nt]
Salmonella phage Lumpael (NC_048113) [41,126 nt]
Enterobacteria phage IME_EC2 (NC_047742) [41,510 nt]
Klebsiella phage vB_KpnS_IME279 (NC_047862) [42,518 nt]
Escherichia phage Sortsne (NC_048178) [41,912 nt]
Sodalis phage phiSG1 (NC_007902) [52,162 nt]
Hamiltonella phage APSE-1 (NC_000935) [36,524 nt]
Bacteriophage APSE-2 (NC_011551) [39,867 nt]
Enterobacteria phage Sf101 (NC_027398) [38,742 nt]
Enterobacteria phage CUS-3 (CP000711) [40,207 nt]
Enterobacteria phage HK620 (NC_002730) [38,297 nt]
Shigella phage Sf6 (NC_005344) [39,043 nt]
Enterobacteria phage IME10 (NC_019501) [39,646 nt]
Salmonella phage SEN22 (NC_028696) [41,338 nt]
Salmonella phage g341c (NC_013059) [40,975 nt]
Salmonella phage epsilon34 (NC_011976) [43,016 nt]
Salmonella phage vB_SemP_Emek (NC_018275) [39,783 nt]
Salmonella phage UPF_BP1 (NC_047875) [39,902 nt]
Lederbergvirus BTP1 (NC_042346) [40,525 nt]
Salmonella phage ST64T (NC_004348) [40,679 nt]
Enterobacteria phage ST104 (NC_005841) [41,391 nt]
Salmonella phage ST160 (NC_014900) [40,986 nt]
Salmonella phage SE1 (in:P22virus) (NC_011802) [41,941 nt]
Salmonella phage SPN9CC (NC_017985) [40,128 nt]
Salmonella phage 118970_sal4 (NC_030919) [42,418 nt]
Salmonella phage 103203_sal5 (NC_031946) [40,443 nt]
Enterobacteria phage UAB_Phi20 (NC_031019) [41,809 nt]
Salmonella phage P22-pbi (AF527608) [41,724 nt]
Salmonella phage P22 (NC_002371) [41,724 nt]
Sinorhizobium phage phiLM21 (NC_029046) [50,827 nt]

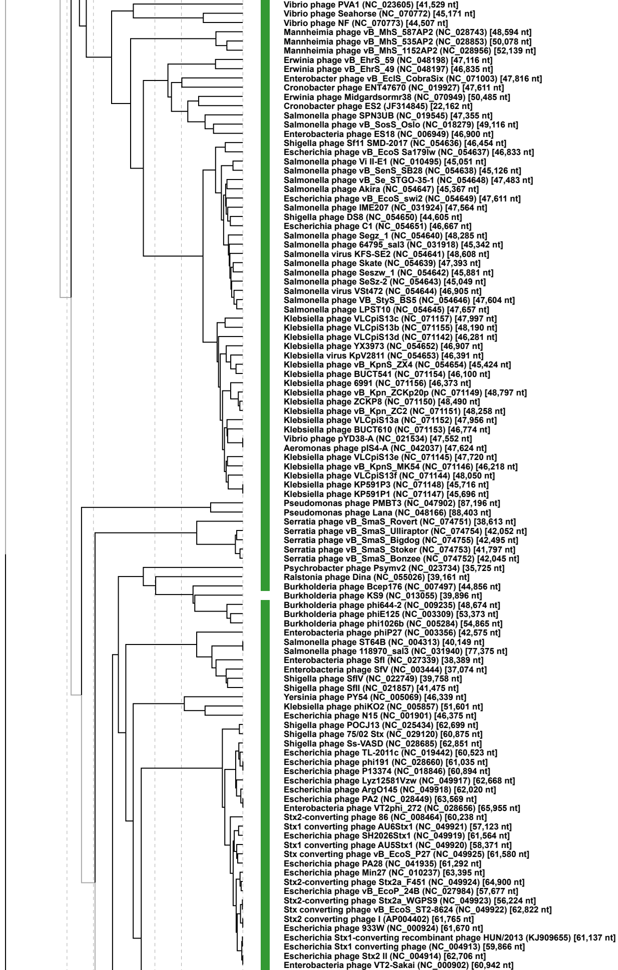
Vibrio phage PVA1 (NC_023605) [41,529 nt]
Vibrio phage Seahorse (NC_070772) [45,171 nt]
Vibrio phage NF (NC_070773) [44,507 nt]
Mannheimia phage vB_MhS_587AP2 (NC_028743) [48,594 nt]
Mannheimia phage vB_MhS_535AP2 (NC_028853) [50,078 nt]
Mannheimia phage vB_MhS_1152AP2 (NC_028956) [52,139 nt]
Erwinia phage vB_EhrS_59 (NC_048198) [47,116 nt]
Erwinia phage vB_EhrS_49 (NC_048197) [46,835 nt]
Enterobacter phage vB_EclS_CobraSix (NC_071003) [47,816 nt]
Cronobacter phage ENT47670 (NC_019927) [47,611 nt]
Erwinia phage Midgardsormr38 (NC_070949) [50,485 nt]
Cronobacter phage ES2 (JF314845) [22,162 nt]
Salmonella phage SPN3UB (NC_019545) [47,355 nt]
Salmonella phage vB_SosS_Oslo (NC_018279) [49,116 nt]
Enterobacteria phage ES18 (NC_006949) [46,900 nt]
Shigella phage Sf11 SMD-2017 (NC_054636) [46,454 nt]
Escherichia phage vB_EcoS Sa179lw (NC_054637) [46,833 nt]
Salmonella phage Vi II-E1 (NC_010495) [45,051 nt]
Salmonella phage vB_SenS_SB28 (NC_054638) [45,126 nt]
Salmonella phage vB_Se_STGO-35-1 (NC_054648) [47,483 nt]
Salmonella phage Akira (NC_054647) [45,367 nt]
Escherichia phage vB_EcoS_swi2 (NC_054649) [47,611 nt]
Salmonella phage IME207 (NC_031924) [47,564 nt]
Shigella phage DS8 (NC_054650) [44,605 nt]
Escherichia phage C1 (NC_054651) [46,667 nt]
Salmonella phage Segz_1 (NC_054640) [48,285 nt]
Salmonella phage 64795_sal3 (NC_031918) [45,342 nt]
Salmonella virus KFS-SE2 (NC_054641) [48,608 nt]
Salmonella phage Skate (NC_054639) [47,393 nt]
Salmonella phage Seszw_1 (NC_054642) [45,881 nt]
Salmonella phage SeSz-2 (NC_054643) [45,049 nt]
Salmonella virus VSt472 (NC_054644) [46,905 nt]
Salmonella phage VB_StyS_BS5 (NC_054646) [47,604 nt]
Salmonella phage LPST10 (NC_054645) [47,657 nt]
Klebsiella phage VLCpiS13c (NC_071157) [47,997 nt]
Klebsiella phage VLCpiS13b (NC_071155) [48,190 nt]
Klebsiella phage VLCpiS13d (NC_071142) [46,281 nt]
Klebsiella phage YX3973 (NC_054652) [46,907 nt]
Klebsiella virus KpV2811 (NC_054653) [46,391 nt]
Klebsiella phage vB_KpnS_ZX4 (NC_054654) [45,424 nt]
Klebsiella phage BUCT541 (NC_071154) [46,100 nt]
Klebsiella phage 6991 (NC_071156) [46,373 nt]
Klebsiella phage vB_Kpn_ZCKp20p (NC_071149) [48,797 nt]
Klebsiella phage ZCKP8 (NC_071150) [48,490 nt]
Klebsiella phage vB_Kpn_ZC2 (NC_071151) [48,258 nt]
Klebsiella phage VLCpiS13a (NC_071152) [47,956 nt]
Klebsiella phage BUCT610 (NC_071153) [46,774 nt]
Vibrio phage pYD38-A (NC_021534) [47,552 nt]
Aeromonas phage pIS4-A (NC_042037) [47,624 nt]
Klebsiella phage VLCpiS13e (NC_071145) [47,720 nt]
Klebsiella phage vB_KpnS_MK54 (NC_071146) [46,218 nt]
Klebsiella phage VLCpiS13f (NC_071144) [48,050 nt]
Klebsiella phage KP591P3 (NC_071148) [45,716 nt]
Klebsiella phage KP591P1 (NC_071147) [45,696 nt]
Pseudomonas phage PMBT3 (NC_047902) [87,196 nt]
Pseudomonas phage Lana (NC_048166) [88,403 nt]
Serratia phage vB_SmaS_Rovert (NC_074751) [38,613 nt]
Serratia phage vB_SmaS_Ulliraptor (NC_074754) [42,052 nt]
Serratia phage vB_SmaS_Bigdog (NC_074755) [42,495 nt]
Serratia phage vB_SmaS_Stoker (NC_074753) [41,797 nt]
Serratia phage vB_SmaS_Bonzee (NC_074752) [42,045 nt]
Psychrobacter phage Psymv2 (NC_023734) [35,725 nt]
Ralstonia phage Dina (NC_055026) [39,161 nt]
Burkholderia phage Bcep176 (NC_007497) [44,856 nt]
Burkholderia phage KS9 (NC_013055) [39,896 nt]
Burkholderia phage phi644-2 (NC_009235) [48,674 nt]
Burkholderia phage phiE125 (NC_003309) [53,373 nt]
Burkholderia phage phi1026b (NC_005284) [54,865 nt]
Enterobacteria phage phiP27 (NC_003356) [42,575 nt]
Salmonella phage ST64B (NC_004313) [40,149 nt]
Salmonella phage 118970_sal3 (NC_031940) [77,375 nt]
Enterobacteria phage SfI (NC_027339) [38,389 nt]
Enterobacteria phage SfV (NC_003444) [37,074 nt]
Shigella phage SfIV (NC_022749) [39,758 nt]
Shigella phage SfII (NC_021857) [41,475 nt]
Yersinia phage PY54 (NC_005069) [46,339 nt]
Klebsiella phage phiKO2 (NC_005857) [51,601 nt]
Escherichia phage N15 (NC_001901) [46,375 nt]
Shigella phage POCJ13 (NC_025434) [62,699 nt]
Shigella phage 75/02 Stx (NC_029120) [60,875 nt]
Shigella phage Ss-VASD (NC_028685) [62,851 nt]
Escherichia phage TL-2011c (NC_019442) [60,523 nt]
Escherichia phage phi191 (NC_028660) [61,035 nt]
Escherichia phage P13374 (NC_018846) [60,894 nt]
Escherichia phage Lyz12581Vzw (NC_049917) [62,668 nt]
Escherichia phage ArgO145 (NC_049918) [62,020 nt]
Escherichia phage PA2 (NC_028449) [63,569 nt]
Enterobacteria phage VT2phi_272 (NC_028656) [65,955 nt]
Stx2-converting phage 86 (NC_008464) [60,238 nt]
Stx1 converting phage AU6Stx1 (NC_049921) [57,123 nt]
Escherichia phage SH2026Stx1 (NC_049919) [61,564 nt]
Stx1 converting phage AU5Stx1 (NC_049920) [58,371 nt]
Stx converting phage vB_EcoS_P27 (NC_049925) [61,580 nt]
Escherichia phage PA28 (NC_041935) [61,292 nt]
Escherichia phage Min27 (NC_010237) [63,395 nt]
Stx2-converting phage Stx2a_F451 (NC_049924) [64,900 nt]
Escherichia phage vB_EcoP_24B (NC_027984) [57,677 nt]
Stx2-converting phage Stx2a_WGPS9 (NC_049923) [56,224 nt]
Stx converting phage vB_EcoS_ST2-8624 (NC_049922) [62,822 nt]
Stx2 converting phage I (AP004402) [61,765 nt]
Escherichia phage 933W (NC_000924) [61,670 nt]
Escherichia Stx1-converting recombinant phage HUN/2013 (KJ909655) [61,137 nt]
Escherichia Stx1 converting phage (NC_004913) [59,866 nt]
Escherichia phage Stx2 II (NC_004914) [62,706 nt]
Enterobacteria phage VT2-Sakai (NC_000902) [60,942 nt]

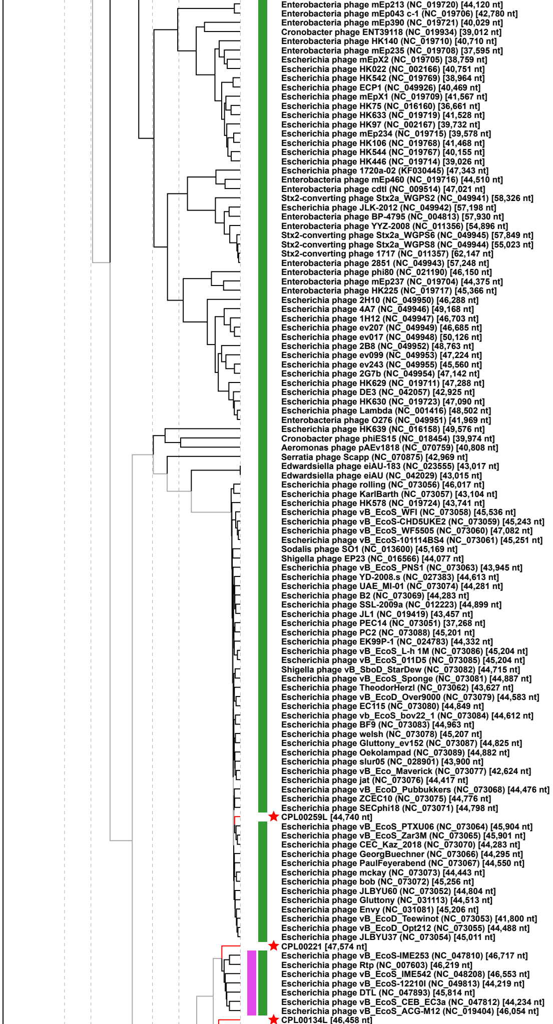
Enterobacteria phage mEp213 (NC_019720) [44,120 nt]
Enterobacteria phage mEp043 c-1 (NC_019706) [42,780 nt]
Enterobacteria phage mEp390 (NC_019721) [40,029 nt]
Cronobacter phage ENT39118 (NC_019934) [39,012 nt]
Enterobacteria phage HK140 (NC_019710) [40,710 nt]
Enterobacteria phage mEp235 (NC_019708) [37,595 nt]
Escherichia phage mEpX2 (NC_019705) [38,759 nt]
Escherichia phage HK022 (NC_002166) [40,751 nt]
Escherichia phage HK542 (NC_019769) [38,964 nt]
Escherichia phage ECP1 (NC_049926) [40,469 nt]
Escherichia phage mEpX1 (NC_019709) [41,567 nt]
Escherichia phage HK75 (NC_016160) [36,661 nt]
Escherichia phage HK633 (NC_019719) [41,528 nt]
Escherichia phage HK97 (NC_002167) [39,732 nt]
Escherichia phage mEp234 (NC_019715) [39,578 nt]
Escherichia phage HK106 (NC_019768) [41,468 nt]
Escherichia phage HK544 (NC_019767) [40,155 nt]
Escherichia phage HK446 (NC_019714) [39,026 nt]
Escherichia phage 1720a-02 (KF030445) [47,343 nt]
Enterobacteria phage mEp460 (NC_019716) [44,510 nt]
Enterobacteria phage cdtI (NC_009514) [47,021 nt]
Stx2-converting phage Stx2a_WGPS2 (NC_049941) [58,326 nt]
Escherichia phage JLK-2012 (NC_049942) [57,198 nt]
Enterobacteria phage BP-4795 (NC_004813) [57,930 nt]
Enterobacteria phage YYZ-2008 (NC_011356) [54,896 nt]
Stx2-converting phage Stx2a_WGPS6 (NC_049945) [57,849 nt]
Stx2-converting phage Stx2a_WGPS8 (NC_049944) [55,023 nt]
Stx2-converting phage 1717 (NC_011357) [62,147 nt]
Enterobacteria phage 2851 (NC_049943) [57,248 nt]
Enterobacteria phage phi80 (NC_021190) [46,150 nt]
Enterobacteria phage mEp237 (NC_019704) [44,375 nt]
Enterobacteria phage HK225 (NC_019717) [45,366 nt]
Escherichia phage 2H10 (NC_049950) [46,288 nt]
Escherichia phage 4A7 (NC_049946) [49,168 nt]
Escherichia phage 1H12 (NC_049947) [46,703 nt]
Escherichia phage ev207 (NC_049949) [46,685 nt]
Escherichia phage ev017 (NC_049948) [50,126 nt]
Escherichia phage 2B8 (NC_049952) [48,763 nt]
Escherichia phage ev099 (NC_049953) [47,224 nt]
Escherichia phage ev243 (NC_049955) [45,560 nt]
Escherichia phage 2G7b (NC_049954) [47,142 nt]
Escherichia phage HK629 (NC_019711) [47,288 nt]
Escherichia phage DE3 (NC_042057) [42,925 nt]
Escherichia phage HK630 (NC_019723) [47,090 nt]
Escherichia phage Lambda (NC_001416) [48,502 nt]
Enterobacteria phage O276 (NC_049951) [41,969 nt]
Escherichia phage HK639 (NC_016158) [49,576 nt]
Cronobacter phage phiES15 (NC_018454) [39,974 nt]
Aeromonas phage pAEv1818 (NC_070759) [40,808 nt]
Serratia phage Scapp (NC_070875) [42,969 nt]
Edwardsiella phage eiAU-183 (NC_023555) [43,017 nt]
Edwardsiella phage eiAU (NC_042029) [43,015 nt]
Escherichia phage rolling (NC_073056) [46,017 nt]
Escherichia phage KarlBarth (NC_073057) [43,104 nt]
Escherichia phage HK578 (NC_019724) [43,741 nt]
Escherichia phage vB_EcoS_WFI (NC_073058) [45,536 nt]
Escherichia phage vB_EcoS-CHD5UKE2 (NC_073059) [45,243 nt]
Escherichia phage vB_EcoS_WF5505 (NC_073060) [47,082 nt]
Escherichia phage vB_EcoS-101114BS4 (NC_073061) [45,251 nt]
Sodalis phage SO1 (NC_013600) [45,169 nt]
Shigella phage EP23 (NC_016566) [44,077 nt]
Escherichia phage vB_EcoS_PNS1 (NC_073063) [43,945 nt]
Escherichia phage YD-2008.s (NC_027383) [44,613 nt]
Escherichia phage UAE_MI-01 (NC_073074) [44,281 nt]
Escherichia phage B2 (NC_073069) [44,283 nt]
Escherichia phage SSL-2009a (NC_012223) [44,899 nt]
Escherichia phage JL1 (NC_019419) [43,457 nt]
Escherichia phage PEC14 (NC_073051) [37,268 nt]
Escherichia phage PC2 (NC_073088) [45,201 nt]
Escherichia phage EK99P-1 (NC_024783) [44,332 nt]
Escherichia phage vB_EcoS_L-h 1M (NC_073086) [45,204 nt]
Escherichia phage vB_EcoS_011D5 (NC_073085) [45,204 nt]
Shigella phage vB_SboD_StarDew (NC_073082) [44,715 nt]
Escherichia phage vB_EcoS_Sponge (NC_073081) [44,887 nt]
Escherichia phage TheodorHerzl (NC_073062) [43,627 nt]
Escherichia phage vB_EcoD_Over9000 (NC_073079) [44,583 nt]
Escherichia phage EC115 (NC_073080) [44,849 nt]
Escherichia phage vb_EcoS_bov22_1 (NC_073084) [44,612 nt]
Escherichia phage BF9 (NC_073083) [44,963 nt]
Escherichia phage welsh (NC_073078) [45,207 nt]
Escherichia phage Gluttony_ev152 (NC_073087) [44,825 nt]
Escherichia phage Oekolampad (NC_073089) [44,882 nt]
Escherichia phage slur05 (NC_028901) [43,900 nt]
Escherichia phage vB_Eco_Maverick (NC_073077) [42,624 nt]
Escherichia phage jat (NC_073076) [44,417 nt]
Escherichia phage vB_EcoD_Pubbukkers (NC_073068) [44,476 nt]
Escherichia phage ZCEC10 (NC_073075) [44,776 nt]
Escherichia phage SECphi18 (NC_073071) [44,798 nt]
CPL00259L [44,740 nt]
Escherichia phage vB_EcoS_PTXU06 (NC_073064) [45,904 nt]
Escherichia phage vB_EcoS_Zar3M (NC_073065) [45,901 nt]
Escherichia phage CEC_Kaz_2018 (NC_073070) [44,283 nt]
Escherichia phage GeorgBuechner (NC_073066) [44,295 nt]
Escherichia phage PaulFeyerabend (NC_073067) [44,550 nt]
Escherichia phage mckay (NC_073073) [44,443 nt]
Escherichia phage bob (NC_073072) [45,256 nt]
Escherichia phage JLBYU60 (NC_073052) [44,804 nt]
Escherichia phage Gluttony (NC_031113) [44,513 nt]
Escherichia phage Envy (NC_031081) [45,206 nt]
Escherichia phage vB_EcoD_Teewinot (NC_073053) [41,800 nt]
Escherichia phage vB_EcoD_Opt212 (NC_073055) [44,488 nt]
Escherichia phage JLBYU37 (NC_073054) [45,011 nt]
CPL00221 [47,574 nt]
Escherichia phage vB_EcoS-IME253 (NC_047810) [46,717 nt]
Escherichia phage Rtp (NC_007603) [46,219 nt]
Escherichia phage vB_EcoS_IME542 (NC_048208) [46,553 nt]
Escherichia phage vB_EcoS-12210I (NC_049813) [44,219 nt]
Escherichia phage DTL (NC_047893) [45,814 nt]
Escherichia phage vB_EcoS_CEB_EC3a (NC_047812) [44,234 nt]
Escherichia phage vB_EcoS_ACG-M12 (NC_019404) [46,054 nt]
CPL00134L [46,458 nt]

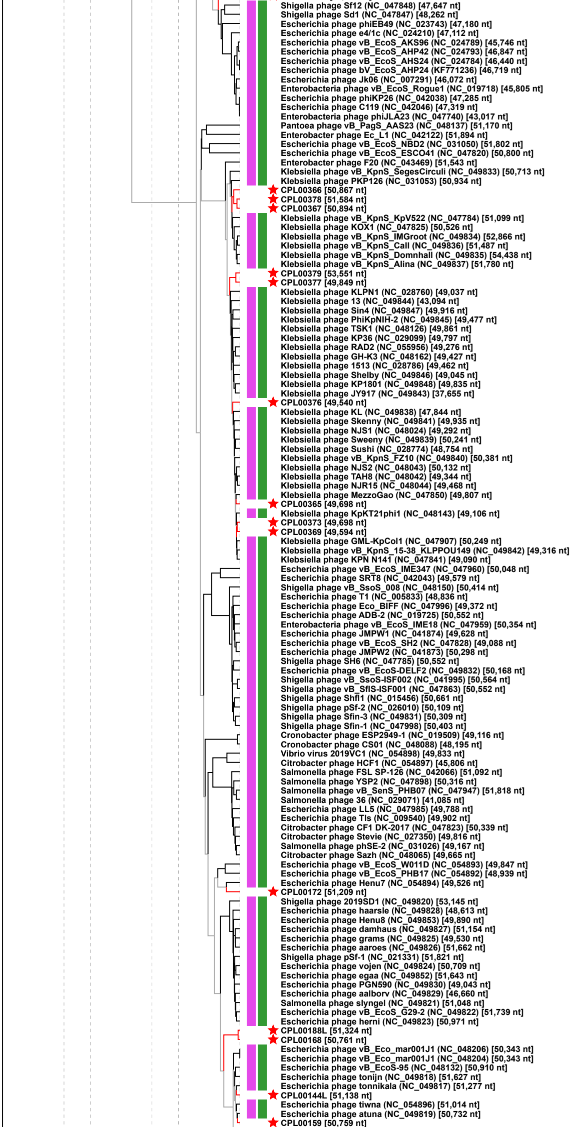

Shigella phage Sf12 (NC_047848) [47,647 nt]
Shigella phage Sd1 (NC_047847) [48,262 nt]
Escherichia phage phiEB49 (NC_023743) [47,180 nt]
Escherichia phage e4/1c (NC_024210) [47,112 nt]
Escherichia phage vB_EcoS_AKS96 (NC_024789) [45,746 nt]
Escherichia phage vB_EcoS_AHP42 (NC_024793) [46,847 nt]
Escherichia phage vB_EcoS_AHS24 (NC_024784) [46,440 nt]
Escherichia phage bV_EcoS_AHP24 (KF771236) [46,719 nt]
Escherichia phage Jk06 (NC_007291) [46,072 nt]
Enterobacteria phage vB_EcoS_Rogue1 (NC_019718) [45,805 nt]
Escherichia phage phiKP26 (NC_042038) [47,285 nt]
Escherichia phage C119 (NC_042046) [47,319 nt]
Enterobacteria phage phiJLA23 (NC_047740) [43,017 nt]
Pantoea phage vB_PagS_AAS23 (NC_048137) [51,170 nt]
Enterobacter phage Ec_L1 (NC_042122) [51,894 nt]
Escherichia phage vB_EcoS_NBD2 (NC_031050) [51,802 nt]
Escherichia phage vB_EcoS_ESCO41 (NC_047820) [50,800 nt]
Enterobacter phage F20 (NC_043469) [51,543 nt]
Klebsiella phage vB_KpnS_SegesCirculi (NC_049833) [50,713 nt]
Klebsiella phage PKP126 (NC_031053) [50,934 nt]
CPL00366 [50,867 nt]
CPL00378 [51,584 nt]
CPL00367 [50,894 nt]
Klebsiella phage vB_KpnS_KpV522 (NC_047784) [51,099 nt]
Klebsiella phage KOX1 (NC_047825) [50,526 nt]
Klebsiella phage vB_KpnS_IMGroot (NC_049834) [52,866 nt]
Klebsiella phage vB_KpnS_Call (NC_049836) [51,487 nt]
Klebsiella phage vB_KpnS_Domnhall (NC_049835) [54,438 nt]
Klebsiella phage vB_KpnS_Alina (NC_049837) [51,780 nt]
CPL00379 [53,551 nt]
CPL00377 [49,849 nt]
Klebsiella phage KLPN1 (NC_028760) [49,037 nt]
Klebsiella phage 13 (NC_049844) [43,094 nt]
Klebsiella phage Sin4 (NC_049847) [49,916 nt]
Klebsiella phage PhiKpNIH-2 (NC_049845) [49,477 nt]
Klebsiella phage TSK1 (NC_048126) [49,861 nt]
Klebsiella phage KP36 (NC_029099) [49,797 nt]
Klebsiella phage RAD2 (NC_055956) [49,276 nt]
Klebsiella phage GH-K3 (NC_048162) [49,427 nt]
Klebsiella phage 1513 (NC_028786) [49,462 nt]
Klebsiella phage Shelby (NC_049846) [49,045 nt]
Klebsiella phage KP1801 (NC_049848) [49,835 nt]
Klebsiella phage JY917 (NC_049843) [37,655 nt]
CPL00376 [49,540 nt]
Klebsiella phage KL (NC_049838) [47,844 nt]
Klebsiella phage Skenny (NC_049841) [49,935 nt]
Klebsiella phage NJS1 (NC_048024) [49,292 nt]
Klebsiella phage Sweeny (NC_049839) [50,241 nt]
Klebsiella phage Sushi (NC_028774) [48,754 nt]
Klebsiella phage vB_KpnS_FZ10 (NC_049840) [50,381 nt]
Klebsiella phage NJS2 (NC_048043) [50,132 nt]
Klebsiella phage TAH8 (NC_048042) [49,344 nt]
Klebsiella phage NJR15 (NC_048044) [49,468 nt]
Klebsiella phage MezzoGao (NC_047850) [49,807 nt]
CPL00365 [49,698 nt]
Klebsiella phage KpKT21phi1 (NC_048143) [49,106 nt]
CPL00373 [49,698 nt]
CPL00369 [49,594 nt]
Klebsiella phage GML-KpCol1 (NC_047907) [50,249 nt]
Klebsiella phage vB_KpnS_15-38_KLPPOU149 (NC_049842) [49,316 nt]
Klebsiella phage KPN N141 (NC_047841) [49,090 nt]
Escherichia phage vB_EcoS_IME347 (NC_047960) [50,048 nt]
Escherichia phage SRT8 (NC_042043) [49,579 nt]
Shigella phage vB_SsoS_008 (NC_048150) [50,414 nt]
Escherichia phage T1 (NC_005833) [48,836 nt]
Escherichia phage Eco_BIFF (NC_047996) [49,372 nt]
Escherichia phage ADB-2 (NC_019725) [50,552 nt]
Enterobacteria phage vB_EcoS_IME18 (NC_047959) [50,354 nt]
Escherichia phage JMPW1 (NC_041874) [49,628 nt]
Escherichia phage vB_EcoS_SH2 (NC_047828) [49,088 nt]
Escherichia phage JMPW2 (NC_041873) [50,298 nt]
Shigella phage SH6 (NC_047785) [50,552 nt]
Escherichia phage vB_EcoS-DELF2 (NC_049832) [50,168 nt]
Shigella phage vB_SsoS-ISF002 (NC_041995) [50,564 nt]
Shigella phage vB_SflS-ISF001 (NC_047863) [50,552 nt]
Shigella phage Shfl1 (NC_015456) [50,661 nt]
Shigella phage pSf-2 (NC_026010) [50,109 nt]
Shigella phage Sfin-3 (NC_049831) [50,309 nt]
Shigella phage Sfin-1 (NC_047998) [50,403 nt]
Cronobacter phage ESP2949-1 (NC_019509) [49,116 nt]
Cronobacter phage CS01 (NC_048088) [48,195 nt]
Vibrio virus 2019VC1 (NC_054898) [49,833 nt]
Citrobacter phage HCF1 (NC_054897) [45,806 nt]
Salmonella phage FSL SP-126 (NC_042066) [51,092 nt]
Salmonella phage YSP2 (NC_047898) [50,316 nt]
Salmonella phage vB_SenS_PHB07 (NC_047947) [51,818 nt]
Salmonella phage 36 (NC_029071) [41,085 nt]
Escherichia phage LL5 (NC_047985) [49,788 nt]
Escherichia phage Tls (NC_009540) [49,902 nt]
Citrobacter phage CF1 DK-2017 (NC_047823) [50,339 nt]
Citrobacter phage Stevie (NC_027350) [49,816 nt]
Salmonella phage phSE-2 (NC_031026) [49,167 nt]
Citrobacter phage Sazh (NC_048065) [49,665 nt]
Escherichia phage vB_EcoS_W011D (NC_054893) [49,847 nt]
Escherichia phage vB_EcoS_PHB17 (NC_054892) [48,939 nt]
Escherichia phage Henu7 (NC_054894) [49,526 nt]
CPL00172 [51,209 nt]
Shigella phage 2019SD1 (NC_049820) [53,145 nt]
Escherichia phage haarsle (NC_049828) [48,613 nt]
Escherichia phage Henu8 (NC_049853) [49,890 nt]
Escherichia phage damhaus (NC_049827) [51,154 nt]
Escherichia phage grams (NC_049825) [49,530 nt]
Escherichia phage aaroes (NC_049826) [51,662 nt]
Shigella phage pSf-1 (NC_021331) [51,821 nt]
Escherichia phage vojen (NC_049824) [50,709 nt]
Escherichia phage egaa (NC_049852) [51,643 nt]
Escherichia phage PGN590 (NC_049830) [49,043 nt]
Escherichia phage aalborv (NC_049829) [46,660 nt]
Salmonella phage slyngel (NC_049821) [51,048 nt]
Escherichia phage vB_EcoS_G29-2 (NC_049822) [51,739 nt]
Escherichia phage herni (NC_049823) [50,971 nt]
CPL00188L [51,324 nt]
CPL00168 [50,761 nt]
Escherichia phage vB_Eco_mar001J1 (NC_048206) [50,343 nt]
Escherichia phage vB_Eco_mar001J1 (NC_048204) [50,343 nt]
Escherichia phage vB_EcoS-95 (NC_048132) [50,910 nt]
Escherichia phage tonijn (NC_049818) [51,627 nt]
Escherichia phage tonnikala (NC_049817) [51,277 nt]
CPL00144L [51,138 nt]
Escherichia phage tiwna (NC_054896) [51,014 nt]
Escherichia phage atuna (NC_049819) [50,732 nt]
CPL00159 [50,759 nt]

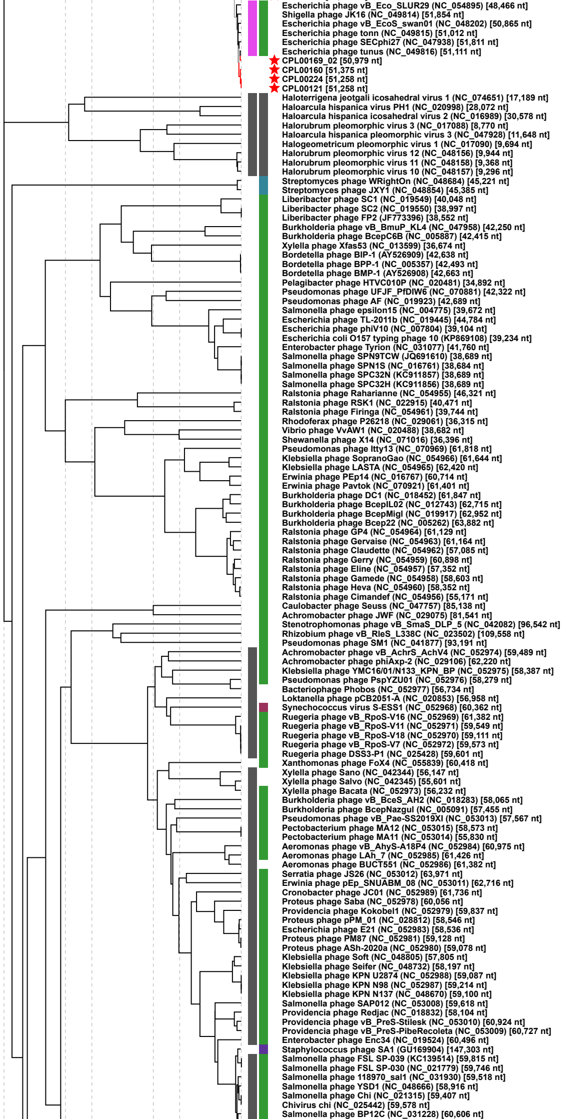
Escherichia phage vB_Eco_SLUR29 (NC_054895) [48,466 nt]
Shigella phage JK16 (NC_049814) [51,854 nt]
Escherichia phage vB_EcoS_swan01 (NC_048202) [50,865 nt]
Escherichia phage tonn (NC_049815) [51,012 nt]
Escherichia phage SECphi27 (NC_047938) [51,811 nt]
Escherichia phage tunus (NC_049816) [51,111 nt]
CPL00169_02 [50,979 nt]
CPL00160 [51,375 nt]
CPL00224 [51,258 nt]
CPL00121 [51,258 nt]
Haloterrigena jeotgali icosahedral virus 1 (NC_074651) [17,189 nt]
Haloarcula hispanica virus PH1 (NC_020998) [28,072 nt]
Haloarcula hispanica icosahedral virus 2 (NC_016989) [30,578 nt]
Halorubrum pleomorphic virus 3 (NC_017088) [8,770 nt]
Haloarcula hispanica pleomorphic virus 3 (NC_047928) [11,648 nt]
Halogeometricum pleomorphic virus 1 (NC_017090) [9,694 nt]
Halorubrum pleomorphic virus 12 (NC_048156) [9,944 nt]
Halorubrum pleomorphic virus 11 (NC_048158) [9,368 nt]
Halorubrum pleomorphic virus 10 (NC_048157) [9,296 nt]
Streptomyces phage WRightOn (NC_048684) [45,221 nt]
Streptomyces phage JXY1 (NC_048854) [45,385 nt]
Liberibacter phage SC1 (NC_019549) [40,048 nt]
Liberibacter phage SC2 (NC_019550) [38,997 nt]
Liberibacter phage FP2 (JF773396) [38,552 nt]
Burkholderia phage vB_BmuP_KL4 (NC_047958) [42,250 nt]
Burkholderia phage BcepC6B (NC_005887) [42,415 nt]
Xylella phage Xfas53 (NC_013599) [36,674 nt]
Bordetella phage BIP-1 (AY526909) [42,638 nt]
Bordetella phage BPP-1 (NC_005357) [42,493 nt]
Bordetella phage BMP-1 (AY526908) [42,663 nt]
Pelagibacter phage HTVC010P (NC_020481) [34,892 nt]
Pseudomonas phage UFJF_PfDIW6 (NC_070881) [42,322 nt]
Pseudomonas phage AF (NC_019923) [42,689 nt]
Salmonella phage epsilon15 (NC_004775) [39,672 nt]
Escherichia phage TL-2011b (NC_019445) [44,784 nt]
Escherichia phage phiV10 (NC_007804) [39,104 nt]
Escherichia coli O157 typing phage 10 (KP869108) [39,234 nt]
Enterobacter phage Tyrion (NC_031077) [41,760 nt]
Salmonella phage SPN9TCW (JQ691610) [38,689 nt]
Salmonella phage SPN1S (NC_016761) [38,684 nt]
Salmonella phage SPC32N (KC911857) [38,689 nt]
Salmonella phage SPC32H (KC911856) [38,689 nt]
Ralstonia phage Raharianne (NC_054955) [46,321 nt]
Ralstonia phage RSK1 (NC_022915) [40,471 nt]
Ralstonia phage Firinga (NC_054961) [39,744 nt]
Rhodoferax phage P26218 (NC_029061) [36,315 nt]
Vibrio phage VvAW1 (NC_020488) [38,682 nt]
Shewanella phage X14 (NC_071016) [36,396 nt]
Pseudomonas phage Itty13 (NC_070969) [61,818 nt]
Klebsiella phage SopranoGao (NC_054966) [61,644 nt]
Klebsiella phage LASTA (NC_054965) [62,420 nt]
Erwinia phage PEp14 (NC_016767) [60,714 nt]
Erwinia phage Pavtok (NC_070921) [61,401 nt]
Burkholderia phage DC1 (NC_018452) [61,847 nt]
Burkholderia phage BcepIL02 (NC_012743) [62,715 nt]
Burkholderia phage BcepMigl (NC_019917) [62,952 nt]
Burkholderia phage Bcep22 (NC_005262) [63,882 nt]
Ralstonia phage GP4 (NC_054964) [61,129 nt]
Ralstonia phage Gervaise (NC_054963) [61,164 nt]
Ralstonia phage Claudette (NC_054962) [57,085 nt]
Ralstonia phage Gerry (NC_054959) [60,898 nt]
Ralstonia phage Eline (NC_054957) [57,352 nt]
Ralstonia phage Gamede (NC_054958) [58,603 nt]
Ralstonia phage Heva (NC_054960) [58,352 nt]
Ralstonia phage Cimandef (NC_054956) [55,171 nt]
Caulobacter phage Seuss (NC_047757) [85,138 nt]
Achromobacter phage JWF (NC_029075) [81,541 nt]
Stenotrophomonas phage vB_SmaS_DLP_5 (NC_042082) [96,542 nt]
Rhizobium phage vB_RleS_L338C (NC_023502) [109,558 nt]
Pseudomonas phage SM1 (NC_041877) [93,191 nt]
Achromobacter phage vB_AchrS_AchV4 (NC_052974) [59,489 nt]
Achromobacter phage phiAxp-2 (NC_029106) [62,220 nt]
Klebsiella phage YMC16/01/N133_KPN_BP (NC_052975) [58,387 nt]
Pseudomonas phage PspYZU01 (NC_052976) [58,279 nt]
Bacteriophage Phobos (NC_052977) [56,734 nt]
Loktanella phage pCB2051-A (NC_020853) [56,958 nt]
Synechococcus virus S-ESS1 (NC_052968) [60,362 nt]
Ruegeria phage vB_RpoS-V16 (NC_052969) [61,382 nt]
Ruegeria phage vB_RpoS-V11 (NC_052971) [59,549 nt]
Ruegeria phage vB_RpoS-V18 (NC_052970) [59,111 nt]
Ruegeria phage vB_RpoS-V7 (NC_052972) [59,573 nt]
Ruegeria phage DSS3-P1 (NC_025428) [59,601 nt]
Xanthomonas phage FoX4 (NC_055839) [60,418 nt]
Xylella phage Sano (NC_042344) [56,147 nt]
Xylella phage Salvo (NC_042345) [55,601 nt]
Xylella phage Bacata (NC_052973) [56,232 nt]
Burkholderia phage vB_BceS_AH2 (NC_018283) [58,065 nt]
Burkholderia phage BcepNazgul (NC_005091) [57,455 nt]
Pseudomonas phage vB_Pae-SS2019XI (NC_053013) [57,567 nt]
Pectobacterium phage MA12 (NC_053015) [58,573 nt]
Pectobacterium phage MA11 (NC_053014) [55,830 nt]
Aeromonas phage vB_AhyS-A18P4 (NC_052984) [60,975 nt]
Aeromonas phage LAh_7 (NC_052985) [61,426 nt]
Aeromonas phage BUCT551 (NC_052986) [61,382 nt]
Serratia phage JS26 (NC_053012) [63,971 nt]
Erwinia phage pEp_SNUABM_08 (NC_053011) [62,716 nt]
Cronobacter phage JC01 (NC_052989) [61,736 nt]
Proteus phage Saba (NC_052978) [60,056 nt]
Providencia phage Kokobel1 (NC_052979) [59,837 nt]
Proteus phage pPM_01 (NC_028812) [58,546 nt]
Escherichia phage E21 (NC_052983) [58,536 nt]
Proteus phage PM87 (NC_052981) [59,128 nt]
Proteus phage ASh-2020a (NC_052980) [59,078 nt]
Klebsiella phage Soft (NC_048805) [57,805 nt]
Klebsiella phage Seifer (NC_048732) [58,197 nt]
Klebsiella phage KPN U2874 (NC_052988) [59,087 nt]
Klebsiella phage KPN N98 (NC_052987) [59,214 nt]
Klebsiella phage KPN N137 (NC_048670) [59,100 nt]
Salmonella phage SAP012 (NC_053008) [59,618 nt]
Providencia phage Redjac (NC_018832) [58,104 nt]
Providencia phage vB_PreS-Stilesk (NC_053010) [60,924 nt]
Providencia phage vB_PreS-PibeRecoleta (NC_053009) [60,727 nt]
Enterobacter phage Enc34 (NC_019524) [60,496 nt]
Staphylococcus phage SA1 (GU169904) [147,303 nt]
Salmonella phage FSL SP-039 (KC139514) [59,815 nt]
Salmonella phage FSL SP-030 (NC_021779) [59,746 nt]
Salmonella phage 118970_sal1 (NC_031930) [59,518 nt]
Salmonella phage YSD1 (NC_048666) [58,916 nt]
Salmonella phage Chi (NC_021315) [59,407 nt]
Chivirus chi (NC_025442) [59,578 nt]
Salmonella phage BP12C (NC_031228) [60,606 nt]

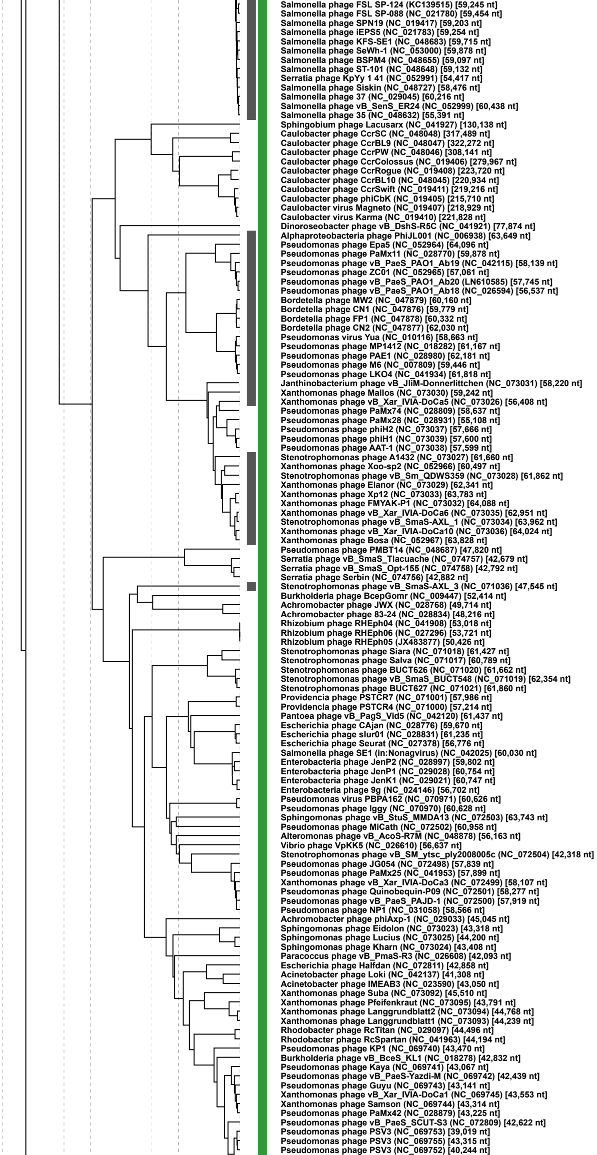

Salmonella phage FSL SP-124 (KC139515) [59,245 nt]
Salmonella phage FSL SP-088 (NC_021780) [59,454 nt]
Salmonella phage SPN19 (NC_019417) [59,203 nt]
Salmonella phage iEPS5 (NC_021783) [59,254 nt]
Salmonella phage KFS-SE1 (NC_048683) [59,715 nt]
Salmonella phage SeWh-1 (NC_053000) [59,878 nt]
Salmonella phage BSPM4 (NC_048655) [59,097 nt]
Salmonella phage ST-101 (NC_048648) [59,132 nt]
Serratia phage KpYy 1 41 (NC_052991) [54,417 nt]
Salmonella phage Siskin (NC_048727) [58,476 nt]
Salmonella phage 37 (NC_029045) [60,216 nt]
Salmonella phage vB_SenS_ER24 (NC_052999) [60,438 nt]
Salmonella phage 35 (NC_048632) [55,391 nt]
Sphingobium phage Lacusarx (NC_041927) [130,138 nt]
Caulobacter phage CcrSC (NC_048048) [317,489 nt]
Caulobacter phage CcrBL9 (NC_048047) [322,272 nt]
Caulobacter phage CcrPW (NC_048046) [308,141 nt]
Caulobacter phage CcrColossus (NC_019406) [279,967 nt]
Caulobacter phage CcrRogue (NC_019408) [223,720 nt]
Caulobacter phage CcrBL10 (NC_048045) [220,934 nt]
Caulobacter phage CcrSwift (NC_019411) [219,216 nt]
Caulobacter phage phiCbK (NC_019405) [215,710 nt]
Caulobacter virus Magneto (NC_019407) [218,929 nt]
Caulobacter virus Karma (NC_019410) [221,828 nt]
Dinoroseobacter phage vB_DshS-R5C (NC_041921) [77,874 nt]
Alphaproteobacteria phage PhiJL001 (NC_006938) [63,649 nt]
Pseudomonas phage Epa5 (NC_052964) [64,096 nt]
Pseudomonas phage PaMx11 (NC_028770) [59,878 nt]
Pseudomonas phage vB_PaeS_PAO1_Ab19 (NC_042115) [58,139 nt]
Pseudomonas phage ZC01 (NC_052965) [57,061 nt]
Pseudomonas phage vB_PaeS_PAO1_Ab20 (LN610585) [57,745 nt]
Pseudomonas phage vB_PaeS_PAO1_Ab18 (NC_026594) [56,537 nt]
Bordetella phage MW2 (NC_047879) [60,160 nt]
Bordetella phage CN1 (NC_047876) [59,779 nt]
Bordetella phage FP1 (NC_047878) [60,332 nt]
Bordetella phage CN2 (NC_047877) [62,030 nt]
Pseudomonas virus Yua (NC_010116) [58,663 nt]
Pseudomonas phage MP1412 (NC_018282) [61,167 nt]
Pseudomonas phage PAE1 (NC_028980) [62,181 nt]
Pseudomonas phage M6 (NC_007809) [59,446 nt]
Pseudomonas phage LKO4 (NC_041934) [61,818 nt]
Janthinobacterium phage vB_JliM-Donnerlittchen (NC_073031) [58,220 nt]
Xanthomonas phage Mallos (NC_073030) [59,242 nt]
Xanthomonas phage vB_Xar_IVIA-DoCa5 (NC_073026) [56,408 nt]
Pseudomonas phage PaMx74 (NC_028809) [58,637 nt]
Pseudomonas phage PaMx28 (NC_028931) [55,108 nt]
Pseudomonas phage phiH2 (NC_073037) [57,666 nt]
Pseudomonas phage phiH1 (NC_073039) [57,600 nt]
Pseudomonas phage AAT-1 (NC_073038) [57,599 nt]
Stenotrophomonas phage A1432 (NC_073027) [61,660 nt]
Xanthomonas phage Xoo-sp2 (NC_052966) [60,497 nt]
Stenotrophomonas phage vB_Sm_QDWS359 (NC_073028) [61,862 nt]
Xanthomonas phage Elanor (NC_073029) [62,341 nt]
Xanthomonas phage Xp12 (NC_073033) [63,783 nt]
Xanthomonas phage FMYAK-P1 (NC_073032) [64,088 nt]
Xanthomonas phage vB_Xar_IVIA-DoCa6 (NC_073035) [62,951 nt]
Stenotrophomonas phage vB_SmaS-AXL_1 (NC_073034) [63,962 nt]
Xanthomonas phage vB_Xar_IVIA-DoCa10 (NC_073036) [64,024 nt]
Xanthomonas phage Bosa (NC_052967) [63,828 nt]
Pseudomonas phage PMBT14 (NC_048687) [47,820 nt]
Serratia phage vB_SmaS_Tlacuache (NC_074757) [42,679 nt]
Serratia phage vB_SmaS_Opt-155 (NC_074758) [42,792 nt]
Serratia phage Serbin (NC_074756) [42,882 nt]
Stenotrophomonas phage vB_SmaS-AXL_3 (NC_071036) [47,545 nt]
Burkholderia phage BcepGomr (NC_009447) [52,414 nt]
Achromobacter phage JWX (NC_028768) [49,714 nt]
Achromobacter phage 83-24 (NC_028834) [48,216 nt]
Rhizobium phage RHEph04 (NC_041908) [53,018 nt]
Rhizobium phage RHEph06 (NC_027296) [53,721 nt]
Rhizobium phage RHEph05 (JX483877) [50,426 nt]
Stenotrophomonas phage Siara (NC_071018) [61,427 nt]
Stenotrophomonas phage Salva (NC_071017) [60,789 nt]
Stenotrophomonas phage BUCT626 (NC_071020) [61,662 nt]
Stenotrophomonas phage vB_SmaS_BUCT548 (NC_071019) [62,354 nt]
Stenotrophomonas phage BUCT627 (NC_071021) [61,860 nt]
Providencia phage PSTCR7 (NC_071001) [57,986 nt]
Providencia phage PSTCR4 (NC_071000) [57,214 nt]
Pantoea phage vB_PagS_Vid5 (NC_042120) [61,437 nt]
Escherichia phage CAjan (NC_028776) [59,670 nt]
Escherichia phage slur01 (NC_028831) [61,235 nt]
Escherichia phage Seurat (NC_027378) [56,776 nt]
Salmonella phage SE1 (in:Nonagvirus) (NC_042025) [60,030 nt]
Enterobacteria phage JenP2 (NC_028997) [59,802 nt]
Enterobacteria phage JenP1 (NC_029028) [60,754 nt]
Enterobacteria phage JenK1 (NC_029021) [60,747 nt]
Enterobacteria phage 9g (NC_024146) [56,702 nt]
Pseudomonas virus PBPA162 (NC_070971) [60,626 nt]
Pseudomonas phage Iggy (NC_070970) [60,628 nt]
Sphingomonas phage vB_StuS_MMDA13 (NC_072503) [63,743 nt]
Pseudomonas phage MiCath (NC_072502) [60,958 nt]
Alteromonas phage vB_AcoS-R7M (NC_048878) [56,163 nt]
Vibrio phage VpKK5 (NC_026610) [56,637 nt]
Stenotrophomonas phage vB_SM_ytsc_ply2008005c (NC_072504) [42,318 nt]
Pseudomonas phage JG054 (NC_072498) [57,839 nt]
Pseudomonas phage PaMx25 (NC_041953) [57,899 nt]
Xanthomonas phage vB_Xar_IVIA-DoCa3 (NC_072499) [58,107 nt]
Pseudomonas phage Quinobequin-P09 (NC_072501) [58,277 nt]
Pseudomonas phage vB_PaeS_PAJD-1 (NC_072500) [57,919 nt]
Pseudomonas phage NP1 (NC_031058) [58,566 nt]
Achromobacter phage phiAxp-1 (NC_029033) [45,045 nt]
Sphingomonas phage Eidolon (NC_073023) [43,318 nt]
Sphingomonas phage Lucius (NC_073025) [44,200 nt]
Sphingomonas phage Kharn (NC_073024) [43,408 nt]
Paracoccus phage vB_PmaS-R3 (NC_026608) [42,093 nt]
Escherichia phage Halfdan (NC_072811) [42,858 nt]
Acinetobacter phage Loki (NC_042137) [41,308 nt]
Acinetobacter phage IMEAB3 (NC_023590) [43,050 nt]
Xanthomonas phage Suba (NC_073092) [45,510 nt]
Xanthomonas phage Pfeifenkraut (NC_073095) [43,791 nt]
Xanthomonas phage Langgrundblatt2 (NC_073094) [44,768 nt]
Xanthomonas phage Langgrundblatt1 (NC_073093) [44,239 nt]
Rhodobacter phage RcTitan (NC_029097) [44,496 nt]
Rhodobacter phage RcSpartan (NC_041963) [44,194 nt]
Pseudomonas phage KP1 (NC_069740) [43,470 nt]
Burkholderia phage vB_BceS_KL1 (NC_018278) [42,832 nt]
Pseudomonas phage Kaya (NC_069741) [43,067 nt]
Pseudomonas phage vB_PaeS-Yazdi-M (NC_069742) [42,439 nt]
Pseudomonas phage Guyu (NC_069743) [43,141 nt]
Xanthomonas phage vB_Xar_IVIA-DoCa1 (NC_069745) [43,553 nt]
Xanthomonas phage Samson (NC_069744) [43,314 nt]
Pseudomonas phage PaMx42 (NC_028879) [43,225 nt]
Pseudomonas phage vB_PaeS_SCUT-S3 (NC_072809) [42,622 nt]
Pseudomonas phage PSV3 (NC_069753) [39,019 nt]
Pseudomonas phage PSV3 (NC_069755) [43,315 nt]
Pseudomonas phage PSV3 (NC_069752) [40,244 nt]

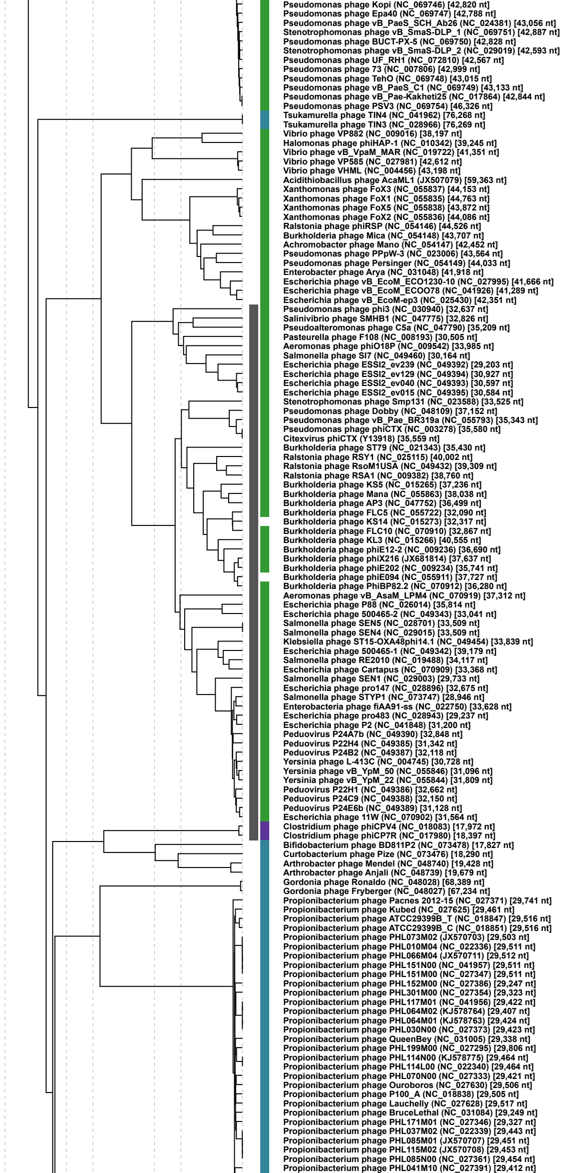

Pseudomonas phage Kopi (NC_069746) [42,820 nt]
Pseudomonas phage Epa40 (NC_069747) [42,788 nt]
Pseudomonas phage vB_PaeS_SCH_Ab26 (NC_024381) [43,056 nt]
Stenotrophomonas phage vB_SmaS-DLP_1 (NC_069751) [42,887 nt]
Pseudomonas phage BUCT-PX-5 (NC_069750) [42,828 nt]
Stenotrophomonas phage vB_SmaS-DLP_2 (NC_029019) [42,593 nt]
Pseudomonas phage UF_RH1 (NC_072810) [42,567 nt]
Pseudomonas phage 73 (NC_007806) [42,999 nt]
Pseudomonas phage TehO (NC_069748) [43,015 nt]
Pseudomonas phage vB_PaeS_C1 (NC_069749) [43,133 nt]
Pseudomonas phage vB_Pae-Kakheti25 (NC_017864) [42,844 nt]
Pseudomonas phage PSV3 (NC_069754) [46,326 nt]
Tsukamurella phage TIN4 (NC_041962) [76,268 nt]
Tsukamurella phage TIN3 (NC_028966) [76,269 nt]
Vibrio phage VP882 (NC_009016) [38,197 nt]
Halomonas phage phiHAP-1 (NC_010342) [39,245 nt]
Vibrio phage vB_VpaM_MAR (NC_019722) [41,351 nt]
Vibrio phage VP585 (NC_027981) [42,612 nt]
Vibrio phage VHML (NC_004456) [43,198 nt]
Acidithiobacillus phage AcaML1 (JX507079) [59,363 nt]
Xanthomonas phage FoX3 (NC_055837) [44,153 nt]
Xanthomonas phage FoX1 (NC_055835) [44,763 nt]
Xanthomonas phage FoX5 (NC_055838) [43,872 nt]
Xanthomonas phage FoX2 (NC_055836) [44,086 nt]
Ralstonia phage phiRSP (NC_054146) [44,526 nt]
Burkholderia phage Mica (NC_054148) [43,707 nt]
Achromobacter phage Mano (NC_054147) [42,452 nt]
Pseudomonas phage PPpW-3 (NC_023006) [43,564 nt]
Pseudomonas phage Persinger (NC_054149) [44,033 nt]
Enterobacter phage Arya (NC_031048) [41,918 nt]
Escherichia phage vB_EcoM_ECO1230-10 (NC_027995) [41,666 nt]
Escherichia phage vB_EcoM_ECOO78 (NC_041926) [41,289 nt]
Escherichia phage vB_EcoM-ep3 (NC_025430) [42,351 nt]
Pseudomonas phage phi3 (NC_030940) [32,637 nt]
Salinivibrio phage SMHB1 (NC_047775) [32,826 nt]
Pseudoalteromonas phage C5a (NC_047790) [35,209 nt]
Pasteurella phage F108 (NC_008193) [30,505 nt]
Aeromonas phage phiO18P (NC_009542) [33,985 nt]
Salmonella phage SI7 (NC_049460) [30,164 nt]
Escherichia phage ESSI2_ev239 (NC_049392) [29,203 nt]
Escherichia phage ESSI2_ev129 (NC_049394) [30,927 nt]
Escherichia phage ESSI2_ev040 (NC_049393) [30,597 nt]
Escherichia phage ESSI2_ev015 (NC_049395) [30,584 nt]
Stenotrophomonas phage Smp131 (NC_023588) [33,525 nt]
Pseudomonas phage Dobby (NC_048109) [37,152 nt]
Pseudomonas phage vB_Pae_BR319a (NC_055793) [35,343 nt]
Pseudomonas phage phiCTX (NC_003278) [35,580 nt]
Citexvirus phiCTX (Y13918) [35,559 nt]
Burkholderia phage ST79 (NC_021343) [35,430 nt]
Ralstonia phage RSY1 (NC_025115) [40,002 nt]
Ralstonia phage RsoM1USA (NC_049432) [39,309 nt]
Ralstonia phage RSA1 (NC_009382) [38,760 nt]
Burkholderia phage KS5 (NC_015265) [37,236 nt]
Burkholderia phage Mana (NC_055863) [38,038 nt]
Burkholderia phage AP3 (NC_047752) [36,499 nt]
Burkholderia phage FLC5 (NC_055722) [32,090 nt]
Burkholderia phage KS14 (NC_015273) [32,317 nt]
Burkholderia phage FLC10 (NC_070910) [32,867 nt]
Burkholderia phage KL3 (NC_015266) [40,555 nt]
Burkholderia phage phiE12-2 (NC_009236) [36,690 nt]
Burkholderia phage phiX216 (JX681814) [37,637 nt]
Burkholderia phage phiE202 (NC_009234) [35,741 nt]
Burkholderia phage phiE094 (NC_055911) [37,727 nt]
Burkholderia phage PhiBP82.2 (NC_070912) [36,280 nt]
Aeromonas phage vB_AsaM_LPM4 (NC_070919) [37,312 nt]
Escherichia phage P88 (NC_026014) [35,814 nt]
Escherichia phage 500465-2 (NC_049343) [33,041 nt]
Salmonella phage SEN5 (NC_028701) [33,509 nt]
Salmonella phage SEN4 (NC_029015) [33,509 nt]
Klebsiella phage ST15-OXA48phi14.1 (NC_049454) [33,839 nt]
Escherichia phage 500465-1 (NC_049342) [39,179 nt]
Salmonella phage RE2010 (NC_019488) [34,117 nt]
Escherichia phage Cartapus (NC_070909) [33,368 nt]
Salmonella phage SEN1 (NC_029003) [29,733 nt]
Escherichia phage pro147 (NC_028896) [32,675 nt]
Salmonella phage STYP1 (NC_073747) [28,946 nt]
Enterobacteria phage fiAA91-ss (NC_022750) [33,628 nt]
Escherichia phage pro483 (NC_028943) [29,237 nt]
Escherichia phage P2 (NC_041848) [31,200 nt]
Peduovirus P24A7b (NC_049390) [32,848 nt]
Peduovirus P22H4 (NC_049385) [31,342 nt]
Peduovirus P24B2 (NC_049387) [32,118 nt]
Yersinia phage L-413C (NC_004745) [30,728 nt]
Yersinia phage vB_YpM_50 (NC_055846) [31,096 nt]
Yersinia phage vB_YpM_22 (NC_055844) [31,809 nt]
Peduovirus P22H1 (NC_049386) [32,662 nt]
Peduovirus P24C9 (NC_049388) [32,150 nt]
Peduovirus P24E6b (NC_049389) [31,128 nt]
Escherichia phage 11W (NC_070902) [31,564 nt]
Clostridium phage phiCPV4 (NC_018083) [17,972 nt]
Clostridium phage phiCP7R (NC_017980) [18,397 nt]
Bifidobacterium phage BD811P2 (NC_073478) [17,827 nt]
Curtobacterium phage Pize (NC_073476) [18,290 nt]
Arthrobacter phage Mendel (NC_048740) [19,428 nt]
Arthrobacter phage Anjali (NC_048739) [19,679 nt]
Gordonia phage Ronaldo (NC_048028) [68,389 nt]
Gordonia phage Fryberger (NC_048027) [67,234 nt]
Propionibacterium phage Pacnes 2012-15 (NC_027371) [29,741 nt]
Propionibacterium phage Kubed (NC_027625) [29,461 nt]
Propionibacterium phage ATCC29399B_T (NC_018847) [29,516 nt]
Propionibacterium phage ATCC29399B_C (NC_018851) [29,516 nt]
Propionibacterium phage PHL073M02 (JX570703) [29,503 nt]
Propionibacterium phage PHL010M04 (NC_022336) [29,511 nt]
Propionibacterium phage PHL066M04 (JX570711) [29,512 nt]
Propionibacterium phage PHL151N00 (NC_041957) [29,511 nt]
Propionibacterium phage PHL151M00 (NC_027347) [29,511 nt]
Propionibacterium phage PHL152M00 (NC_027386) [29,247 nt]
Propionibacterium phage PHL301M00 (NC_027354) [29,323 nt]
Propionibacterium phage PHL117M01 (NC_041956) [29,422 nt]
Propionibacterium phage PHL064M02 (KJ578764) [29,407 nt]
Propionibacterium phage PHL064M01 (KJ578763) [29,424 nt]
Propionibacterium phage PHL030N00 (NC_027373) [29,423 nt]
Propionibacterium phage QueenBey (NC_031005) [29,338 nt]
Propionibacterium phage PHL199M00 (NC_027295) [29,806 nt]
Propionibacterium phage PHL114N00 (KJ578775) [29,464 nt]
Propionibacterium phage PHL114L00 (NC_022340) [29,464 nt]
Propionibacterium phage PHL070N00 (NC_027333) [29,421 nt]
Propionibacterium phage Ouroboros (NC_027630) [29,506 nt]
Propionibacterium phage P100_A (NC_018838) [29,505 nt]
Propionibacterium phage Lauchelly (NC_027628) [29,517 nt]
Propionibacterium phage BruceLethal (NC_031084) [29,249 nt]
Propionibacterium phage PHL171M01 (NC_027346) [29,327 nt]
Propionibacterium phage PHL037M02 (NC_022339) [29,443 nt]
Propionibacterium phage PHL085M01 (JX570707) [29,451 nt]
Propionibacterium phage PHL115M02 (JX570708) [29,453 nt]
Propionibacterium phage PHL085N00 (NC_027361) [29,454 nt]
Propionibacterium phage PHL041M10 (NC_027391) [29,412 nt]

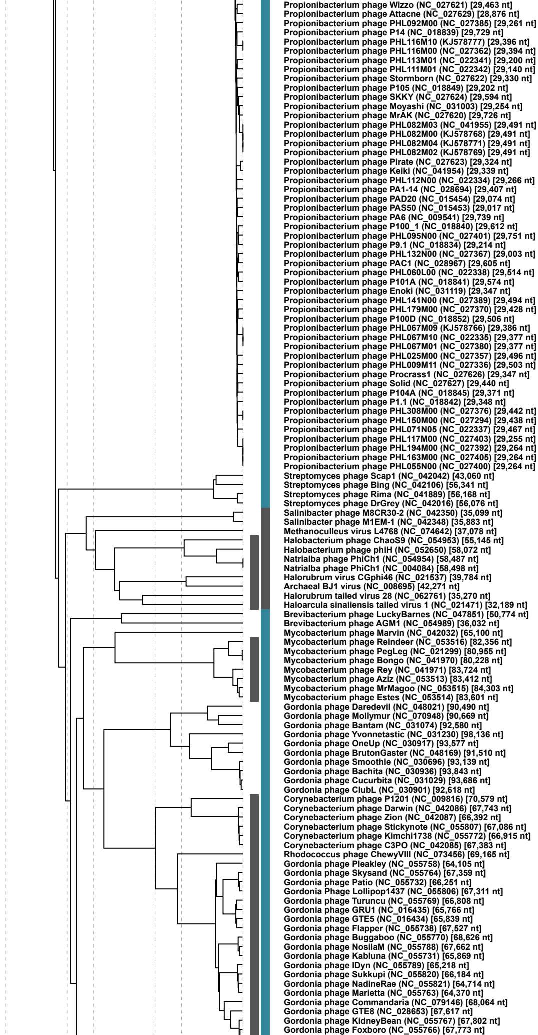

Propionibacterium phage Wizzo (NC_027621) [29,463 nt]
Propionibacterium phage Attacne (NC_027629) [28,876 nt]
Propionibacterium phage PHL092M00 (NC_027385) [29,261 nt]
Propionibacterium phage P14 (NC_018839) [29,729 nt]
Propionibacterium phage PHL116M10 (KJ578777) [29,396 nt]
Propionibacterium phage PHL116M00 (NC_027362) [29,394 nt]
Propionibacterium phage PHL113M01 (NC_022341) [29,200 nt]
Propionibacterium phage PHL111M01 (NC_022342) [29,140 nt]
Propionibacterium phage Stormborn (NC_027622) [29,330 nt]
Propionibacterium phage P105 (NC_018849) [29,202 nt]
Propionibacterium phage SKKY (NC_027624) [29,594 nt]
Propionibacterium phage Moyashi (NC_031003) [29,254 nt]
Propionibacterium phage MrAK (NC_027620) [29,726 nt]
Propionibacterium phage PHL082M03 (NC_041955) [29,491 nt]
Propionibacterium phage PHL082M00 (KJ578768) [29,491 nt]
Propionibacterium phage PHL082M04 (KJ578771) [29,491 nt]
Propionibacterium phage PHL082M02 (KJ578769) [29,491 nt]
Propionibacterium phage Pirate (NC_027623) [29,324 nt]
Propionibacterium phage Keiki (NC_041954) [29,339 nt]
Propionibacterium phage PHL112N00 (NC_022334) [29,266 nt]
Propionibacterium phage PA1-14 (NC_028694) [29,407 nt]
Propionibacterium phage PAD20 (NC_015454) [29,074 nt]
Propionibacterium phage PAS50 (NC_015453) [29,017 nt]
Propionibacterium phage PA6 (NC_009541) [29,739 nt]
Propionibacterium phage P100_1 (NC_018840) [29,612 nt]
Propionibacterium phage PHL095N00 (NC_027401) [29,751 nt]
Propionibacterium phage P9.1 (NC_018834) [29,214 nt]
Propionibacterium phage PHL132N00 (NC_027367) [29,003 nt]
Propionibacterium phage PAC1 (NC_028967) [29,605 nt]
Propionibacterium phage PHL060L00 (NC_022338) [29,514 nt]
Propionibacterium phage P101A (NC_018841) [29,574 nt]
Propionibacterium phage Enoki (NC_031119) [29,347 nt]
Propionibacterium phage PHL141N00 (NC_027389) [29,494 nt]
Propionibacterium phage PHL179M00 (NC_027370) [29,428 nt]
Propionibacterium phage P100D (NC_018852) [29,506 nt]
Propionibacterium phage PHL067M09 (KJ578766) [29,386 nt]
Propionibacterium phage PHL067M10 (NC_022335) [29,377 nt]
Propionibacterium phage PHL067M01 (NC_027380) [29,377 nt]
Propionibacterium phage PHL025M00 (NC_027357) [29,496 nt]
Propionibacterium phage PHL009M11 (NC_027336) [29,503 nt]
Propionibacterium phage Procrass1 (NC_027626) [29,347 nt]
Propionibacterium phage Solid (NC_027627) [29,440 nt]
Propionibacterium phage P104A (NC_018845) [29,371 nt]
Propionibacterium phage P1.1 (NC_018842) [29,348 nt]
Propionibacterium phage PHL308M00 (NC_027376) [29,442 nt]
Propionibacterium phage PHL150M00 (NC_027294) [29,438 nt]
Propionibacterium phage PHL071N05 (NC_022337) [29,467 nt]
Propionibacterium phage PHL117M00 (NC_027403) [29,255 nt]
Propionibacterium phage PHL194M00 (NC_027392) [29,264 nt]
Propionibacterium phage PHL163M00 (NC_027405) [29,264 nt]
Propionibacterium phage PHL055N00 (NC_027400) [29,264 nt]
Streptomyces phage Scap1 (NC_042042) [43,060 nt]
Streptomyces phage Bing (NC_042106) [56,341 nt]
Streptomyces phage Rima (NC_041889) [56,168 nt]
Streptomyces phage DrGrey (NC_042016) [56,076 nt]
Salinibacter phage M8CR30-2 (NC_042350) [35,099 nt]
Salinibacter phage M1EM-1 (NC_042348) [35,883 nt]
Methanoculleus virus L4768 (NC_074642) [37,078 nt]
Halobacterium phage ChaoS9 (NC_054953) [55,145 nt]
Halobacterium phage phiH (NC_052650) [58,072 nt]
Natrialba phage PhiCh1 (NC_054954) [58,487 nt]
Natrialba phage PhiCh1 (NC_004084) [58,498 nt]
Halorubrum virus CGphi46 (NC_021537) [39,784 nt]
Archaeal BJ1 virus (NC_008695) [42,271 nt]
Halorubrum tailed virus 28 (NC_062761) [35,270 nt]
Haloarcula sinaiiensis tailed virus 1 (NC_021471) [32,189 nt]
Brevibacterium phage LuckyBarnes (NC_047851) [50,774 nt]
Brevibacterium phage AGM1 (NC_054989) [36,032 nt]
Mycobacterium phage Marvin (NC_042032) [65,100 nt]
Mycobacterium phage Reindeer (NC_053516) [82,356 nt]
Mycobacterium phage PegLeg (NC_021299) [80,955 nt]
Mycobacterium phage Bongo (NC_041970) [80,228 nt]
Mycobacterium phage Rey (NC_041971) [83,724 nt]
Mycobacterium phage Aziz (NC_053513) [83,412 nt]
Mycobacterium phage MrMagoo (NC_053515) [84,303 nt]
Mycobacterium phage Estes (NC_053514) [83,601 nt]
Gordonia phage Daredevil (NC_048021) [90,490 nt]
Gordonia phage Mollymur (NC_070948) [90,669 nt]
Gordonia phage Bantam (NC_031074) [92,580 nt]
Gordonia phage Yvonnetastic (NC_031230) [98,136 nt]
Gordonia phage OneUp (NC_030917) [93,577 nt]
Gordonia phage BrutonGaster (NC_048169) [91,510 nt]
Gordonia phage Smoothie (NC_030696) [93,139 nt]
Gordonia phage Bachita (NC_030936) [93,843 nt]
Gordonia phage Cucurbita (NC_031029) [93,686 nt]
Gordonia phage ClubL (NC_030901) [92,618 nt]
Corynebacterium phage P1201 (NC_009816) [70,579 nt]
Corynebacterium phage Darwin (NC_042086) [67,743 nt]
Corynebacterium phage Zion (NC_042087) [66,392 nt]
Corynebacterium phage Stickynote (NC_055807) [67,086 nt]
Corynebacterium phage Kimchi1738 (NC_055772) [66,915 nt]
Corynebacterium phage C3PO (NC_042085) [67,383 nt]
Rhodococcus phage ChewyVIII (NC_073456) [69,165 nt]
Gordonia phage Pleakley (NC_055758) [64,105 nt]
Gordonia phage Skysand (NC_055764) [67,359 nt]
Gordonia phage Patio (NC_055732) [66,251 nt]
Gordonia Phage Lollipop1437 (NC_055806) [67,311 nt]
Gordonia phage Turuncu (NC_055769) [66,808 nt]
Gordonia phage GRU1 (NC_016435) [65,766 nt]
Gordonia phage GTE5 (NC_016434) [65,839 nt]
Gordonia phage Flapper (NC_055738) [67,527 nt]
Gordonia phage Buggaboo (NC_055770) [68,626 nt]
Gordonia phage NosilaM (NC_055788) [67,662 nt]
Gordonia phage Kabluna (NC_055731) [65,869 nt]
Gordonia phage IDyn (NC_055789) [65,218 nt]
Gordonia phage Sukkupi (NC_055820) [66,184 nt]
Gordonia phage NadineRae (NC_055821) [64,714 nt]
Gordonia phage Marietta (NC_055763) [64,370 nt]
Gordonia phage Commandaria (NC_079146) [68,064 nt]
Gordonia phage GTE8 (NC_028653) [67,617 nt]
Gordonia phage KidneyBean (NC_055767) [67,802 nt]
Gordonia phage Foxboro (NC_055766) [67,773 nt]

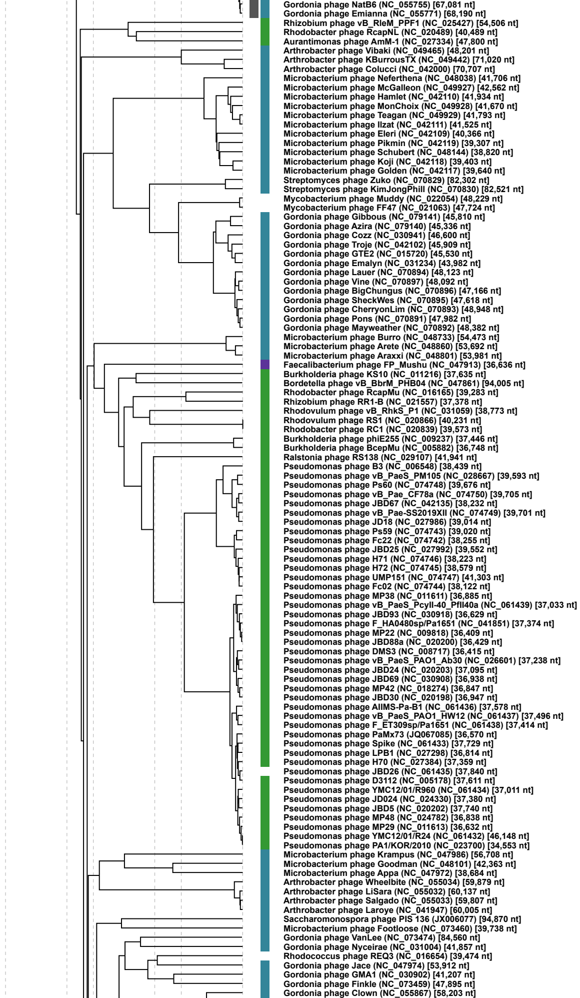
Gordonia phage NatB6 (NC_055755) [67,081 nt]
Gordonia phage Emianna (NC_055771) [68,190 nt]
Rhizobium phage vB_RleM_PPF1 (NC_025427) [54,506 nt]
Rhodobacter phage RcapNL (NC_020489) [40,489 nt]
Aurantimonas phage AmM-1 (NC_027334) [47,800 nt]
Arthrobacter phage Vibaki (NC_049465) [48,201 nt]
Arthrobacter phage KBurrousTX (NC_049442) [71,020 nt]
Arthrobacter phage Colucci (NC_042000) [70,707 nt]
Microbacterium phage Neferthena (NC_048038) [41,706 nt]
Microbacterium phage McGalleon (NC_049927) [42,562 nt]
Microbacterium phage Hamlet (NC_042110) [41,934 nt]
Microbacterium phage MonChoix (NC_049928) [41,670 nt]
Microbacterium phage Teagan (NC_049929) [41,793 nt]
Microbacterium phage Ilzat (NC_042111) [41,525 nt]
Microbacterium phage Eleri (NC_042109) [40,366 nt]
Microbacterium phage Pikmin (NC_042119) [39,307 nt]
Microbacterium phage Schubert (NC_048144) [38,820 nt]
Microbacterium phage Koji (NC_042118) [39,403 nt]
Microbacterium phage Golden (NC_042117) [39,640 nt]
Streptomyces phage Zuko (NC_070829) [82,302 nt]
Streptomyces phage KimJongPhill (NC_070830) [82,521 nt]
Mycobacterium phage Muddy (NC_022054) [48,229 nt]
Mycobacterium phage FF47 (NC_021063) [47,724 nt]
Gordonia phage Gibbous (NC_079141) [45,810 nt]
Gordonia phage Azira (NC_079140) [45,336 nt]
Gordonia phage Cozz (NC_030941) [46,600 nt]
Gordonia phage Troje (NC_042102) [45,909 nt]
Gordonia phage GTE2 (NC_015720) [45,530 nt]
Gordonia phage Emalyn (NC_031234) [43,982 nt]
Gordonia phage Lauer (NC_070894) [48,123 nt]
Gordonia phage Vine (NC_070897) [48,092 nt]
Gordonia phage BigChungus (NC_070896) [47,166 nt]
Gordonia phage SheckWes (NC_070895) [47,618 nt]
Gordonia phage CherryonLim (NC_070893) [48,948 nt]
Gordonia phage Pons (NC_070891) [47,982 nt]
Gordonia phage Mayweather (NC_070892) [48,382 nt]
Microbacterium phage Burro (NC_048733) [54,473 nt]
Microbacterium phage Arete (NC_048860) [53,692 nt]
Microbacterium phage Araxxi (NC_048801) [53,981 nt]
Faecalibacterium phage FP_Mushu (NC_047913) [36,636 nt]
Burkholderia phage KS10 (NC_011216) [37,635 nt]
Bordetella phage vB_BbrM_PHB04 (NC_047861) [94,005 nt]
Rhodobacter phage RcapMu (NC_016165) [39,283 nt]
Rhizobium phage RR1-B (NC_021557) [37,378 nt]
Rhodovulum phage vB_RhkS_P1 (NC_031059) [38,773 nt]
Rhodovulum phage RS1 (NC_020866) [40,231 nt]
Rhodobacter phage RC1 (NC_020839) [39,573 nt]
Burkholderia phage phiE255 (NC_009237) [37,446 nt]
Burkholderia phage BcepMu (NC_005882) [36,748 nt]
Ralstonia phage RS138 (NC_029107) [41,941 nt]
Pseudomonas phage B3 (NC_006548) [38,439 nt]
Pseudomonas phage vB_PaeS_PM105 (NC_028667) [39,593 nt]
Pseudomonas phage Ps60 (NC_074748) [39,676 nt]
Pseudomonas phage vB_Pae_CF78a (NC_074750) [39,705 nt]
Pseudomonas phage JBD67 (NC_042135) [38,232 nt]
Pseudomonas phage vB_Pae-SS2019XII (NC_074749) [39,701 nt]
Pseudomonas phage JD18 (NC_027986) [39,014 nt]
Pseudomonas phage Ps59 (NC_074743) [39,020 nt]
Pseudomonas phage Fc22 (NC_074742) [38,255 nt]
Pseudomonas phage JBD25 (NC_027992) [39,552 nt]
Pseudomonas phage H71 (NC_074746) [38,223 nt]
Pseudomonas phage H72 (NC_074745) [38,579 nt]
Pseudomonas phage UMP151 (NC_074747) [41,303 nt]
Pseudomonas phage Fc02 (NC_074744) [38,122 nt]
Pseudomonas phage MP38 (NC_011611) [36,885 nt]
Pseudomonas phage vB_PaeS_PcyII-40_PfII40a (NC_061439) [37,033 nt]
Pseudomonas phage JBD93 (NC_030918) [36,629 nt]
Pseudomonas phage F_HA0480sp/Pa1651 (NC_041851) [37,374 nt]
Pseudomonas phage MP22 (NC_009818) [36,409 nt]
Pseudomonas phage JBD88a (NC_020200) [36,429 nt]
Pseudomonas phage DMS3 (NC_008717) [36,415 nt]
Pseudomonas phage vB_PaeS_PAO1_Ab30 (NC_026601) [37,238 nt]
Pseudomonas phage JBD24 (NC_020203) [37,095 nt]
Pseudomonas phage JBD69 (NC_030908) [36,938 nt]
Pseudomonas phage MP42 (NC_018274) [36,847 nt]
Pseudomonas phage JBD30 (NC_020198) [36,947 nt]
Pseudomonas phage AIIMS-Pa-B1 (NC_061436) [37,578 nt]
Pseudomonas phage vB_PaeS_PAO1_HW12 (NC_061437) [37,496 nt]
Pseudomonas phage F_ET309sp/Pa1651 (NC_061438) [37,414 nt]
Pseudomonas phage PaMx73 (JQ067085) [36,570 nt]
Pseudomonas phage Spike (NC_061433) [37,729 nt]
Pseudomonas phage LPB1 (NC_027298) [36,814 nt]
Pseudomonas phage H70 (NC_027384) [37,359 nt]
Pseudomonas phage JBD26 (NC_061435) [37,840 nt]
Pseudomonas phage D3112 (NC_005178) [37,611 nt]
Pseudomonas phage YMC12/01/R960 (NC_061434) [37,011 nt]
Pseudomonas phage JD024 (NC_024330) [37,380 nt]
Pseudomonas phage JBD5 (NC_020202) [37,740 nt]
Pseudomonas phage MP48 (NC_024782) [36,838 nt]
Pseudomonas phage MP29 (NC_011613) [36,632 nt]
Pseudomonas phage YMC12/01/R24 (NC_061432) [46,148 nt]
Pseudomonas phage PA1/KOR/2010 (NC_023700) [34,553 nt]
Microbacterium phage Krampus (NC_047986) [56,708 nt]
Microbacterium phage Goodman (NC_048101) [42,363 nt]
Microbacterium phage Appa (NC_047972) [38,684 nt]
Arthrobacter phage Wheelbite (NC_055034) [59,879 nt]
Arthrobacter phage LiSara (NC_055032) [60,137 nt]
Arthrobacter phage Salgado (NC_055033) [59,807 nt]
Arthrobacter phage Laroye (NC_041947) [60,005 nt]
Saccharomonospora phage PIS 136 (JX006077) [94,870 nt]
Microbacterium phage Footloose (NC_073460) [39,738 nt]
Gordonia phage VanLee (NC_073474) [84,560 nt]
Gordonia phage Nyceirae (NC_031004) [41,857 nt]
Rhodococcus phage REQ3 (NC_016654) [39,474 nt]
Gordonia phage Jace (NC_047974) [53,912 nt]
Gordonia phage GMA1 (NC_030902) [41,207 nt]
Gordonia phage Finkle (NC_073459) [47,895 nt]
Gordonia phage Clown (NC_055867) [58,203 nt]

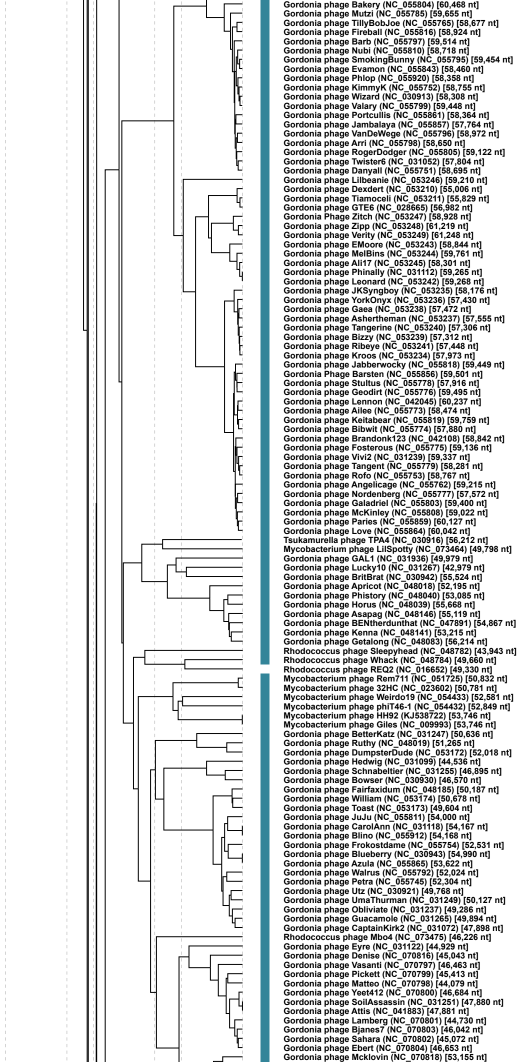
Gordonia phage Bakery (NC_055804) [60,468 nt]
Gordonia phage Mutzi (NC_055785) [59,655 nt]
Gordonia phage TillyBobJoe (NC_055765) [58,677 nt]
Gordonia phage Fireball (NC_055816) [58,924 nt]
Gordonia phage Barb (NC_055797) [59,514 nt]
Gordonia phage Nubi (NC_055810) [58,718 nt]
Gordonia phage SmokingBunny (NC_055795) [59,454 nt]
Gordonia phage Evamon (NC_055843) [58,460 nt]
Gordonia phage Phlop (NC_055920) [58,358 nt]
Gordonia phage KimmyK (NC_055752) [58,755 nt]
Gordonia phage Wizard (NC_030913) [58,308 nt]
Gordonia phage Valary (NC_055799) [59,448 nt]
Gordonia phage Portcullis (NC_055861) [58,364 nt]
Gordonia phage Jambalaya (NC_055857) [57,764 nt]
Gordonia phage VanDeWege (NC_055796) [58,972 nt]
Gordonia phage Arri (NC_055798) [58,650 nt]
Gordonia phage RogerDodger (NC_055805) [59,122 nt]
Gordonia phage Twister6 (NC_031052) [57,804 nt]
Gordonia phage Danyall (NC_055751) [58,695 nt]
Gordonia phage Lilbeanie (NC_053246) [59,210 nt]
Gordonia phage Dexdert (NC_053210) [55,006 nt]
Gordonia phage Tiamoceli (NC_053211) [55,829 nt]
Gordonia phage GTE6 (NC_028665) [56,982 nt]
Gordonia Phage Zitch (NC_053247) [58,928 nt]
Gordonia phage Zipp (NC_053248) [61,219 nt]
Gordonia phage Verity (NC_053249) [61,248 nt]
Gordonia phage EMoore (NC_053243) [58,844 nt]
Gordonia phage MelBins (NC_053244) [59,761 nt]
Gordonia phage Ali17 (NC_053245) [58,301 nt]
Gordonia phage Phinally (NC_031112) [59,265 nt]
Gordonia phage Leonard (NC_053242) [59,268 nt]
Gordonia phage JKSyngboy (NC_053235) [58,176 nt]
Gordonia phage YorkOnyx (NC_053236) [57,430 nt]
Gordonia phage Gaea (NC_053238) [57,472 nt]
Gordonia phage Ashertheman (NC_053237) [57,555 nt]
Gordonia phage Tangerine (NC_053240) [57,306 nt]
Gordonia phage Bizzy (NC_053239) [57,312 nt]
Gordonia phage Ribeye (NC_053241) [57,448 nt]
Gordonia phage Kroos (NC_053234) [57,973 nt]
Gordonia phage Jabberwocky (NC_055818) [59,449 nt]
Gordonia Phage Barsten (NC_055856) [59,501 nt]
Gordonia phage Stultus (NC_055778) [57,916 nt]
Gordonia phage Geodirt (NC_055776) [59,495 nt]
Gordonia phage Lennon (NC_042045) [60,237 nt]
Gordonia phage Ailee (NC_055773) [58,474 nt]
Gordonia phage Keitabear (NC_055819) [59,759 nt]
Gordonia phage Bibwit (NC_055774) [57,880 nt]
Gordonia phage Brandonk123 (NC_042108) [58,842 nt]
Gordonia phage Fosterous (NC_055775) [59,136 nt]
Gordonia phage Vivi2 (NC_031239) [59,337 nt]
Gordonia phage Tangent (NC_055779) [58,281 nt]
Gordonia phage Rofo (NC_055753) [58,767 nt]
Gordonia phage Angelicage (NC_055762) [59,215 nt]
Gordonia phage Nordenberg (NC_055777) [57,572 nt]
Gordonia phage Galadriel (NC_055803) [59,400 nt]
Gordonia phage McKinley (NC_055808) [59,022 nt]
Gordonia phage Paries (NC_055859) [60,127 nt]
Gordonia phage Love (NC_055864) [60,042 nt]
Tsukamurella phage TPA4 (NC_030916) [56,212 nt]
Mycobacterium phage LilSpotty (NC_073464) [49,798 nt]
Gordonia phage GAL1 (NC_031936) [49,979 nt]
Gordonia phage Lucky10 (NC_031267) [42,979 nt]
Gordonia phage BritBrat (NC_030942) [55,524 nt]
Gordonia phage Apricot (NC_048018) [52,195 nt]
Gordonia phage Phistory (NC_048040) [53,085 nt]
Gordonia phage Horus (NC_048039) [55,668 nt]
Gordonia phage Asapag (NC_048146) [55,119 nt]
Gordonia phage BENtherdunthat (NC_047891) [54,867 nt]
Gordonia phage Kenna (NC_048141) [53,215 nt]
Gordonia phage Getalong (NC_048083) [56,214 nt]
Rhodococcus phage Sleepyhead (NC_048782) [43,943 nt]
Rhodococcus phage Whack (NC_048784) [49,660 nt]
Rhodococcus phage REQ2 (NC_016652) [49,330 nt]
Mycobacterium phage Rem711 (NC_051725) [50,832 nt]
Mycobacterium phage 32HC (NC_023602) [50,781 nt]
Mycobacterium phage Weirdo19 (NC_054433) [52,581 nt]
Mycobacterium phage phiT46-1 (NC_054432) [52,849 nt]
Mycobacterium phage HH92 (KJ538722) [53,746 nt]
Mycobacterium phage Giles (NC_009993) [53,746 nt]
Gordonia phage BetterKatz (NC_031247) [50,636 nt]
Gordonia phage Ruthy (NC_048019) [51,265 nt]
Gordonia phage DumpsterDude (NC_053172) [52,018 nt]
Gordonia phage Hedwig (NC_031099) [44,536 nt]
Gordonia phage Schnabeltier (NC_031255) [46,895 nt]
Gordonia phage Bowser (NC_030930) [46,570 nt]
Gordonia phage Fairfaxidum (NC_048185) [50,187 nt]
Gordonia phage William (NC_053174) [50,678 nt]
Gordonia phage Toast (NC_053173) [49,604 nt]
Gordonia phage JuJu (NC_055811) [54,000 nt]
Gordonia phage CarolAnn (NC_031118) [54,167 nt]
Gordonia phage Blino (NC_055912) [54,168 nt]
Gordonia phage Frokostdame (NC_055754) [52,531 nt]
Gordonia phage Blueberry (NC_030943) [54,990 nt]
Gordonia phage Azula (NC_055865) [53,622 nt]
Gordonia phage Walrus (NC_055792) [52,024 nt]
Gordonia phage Petra (NC_055745) [52,304 nt]
Gordonia phage Utz (NC_030921) [49,768 nt]
Gordonia phage UmaThurman (NC_031249) [50,127 nt]
Gordonia phage Obliviate (NC_031237) [49,286 nt]
Gordonia phage Guacamole (NC_031265) [49,894 nt]
Gordonia phage CaptainKirk2 (NC_031072) [47,898 nt]
Rhodococcus phage Mbo4 (NC_073475) [46,226 nt]
Gordonia phage Eyre (NC_031122) [44,929 nt]
Gordonia phage Denise (NC_070816) [45,043 nt]
Gordonia phage Vasanti (NC_070797) [46,463 nt]
Gordonia phage Pickett (NC_070799) [45,413 nt]
Gordonia phage Matteo (NC_070798) [44,079 nt]
Gordonia phage Yeet412 (NC_070800) [46,684 nt]
Gordonia phage SoilAssassin (NC_031251) [47,880 nt]
Gordonia phage Attis (NC_041883) [47,881 nt]
Gordonia phage Lamberg (NC_070801) [44,730 nt]
Gordonia phage Bjanes7 (NC_070803) [46,042 nt]
Gordonia phage Sahara (NC_070802) [45,072 nt]
Gordonia phage Ebert (NC_070804) [46,653 nt]
Gordonia phage Mcklovin (NC_070818) [53,155 nt]

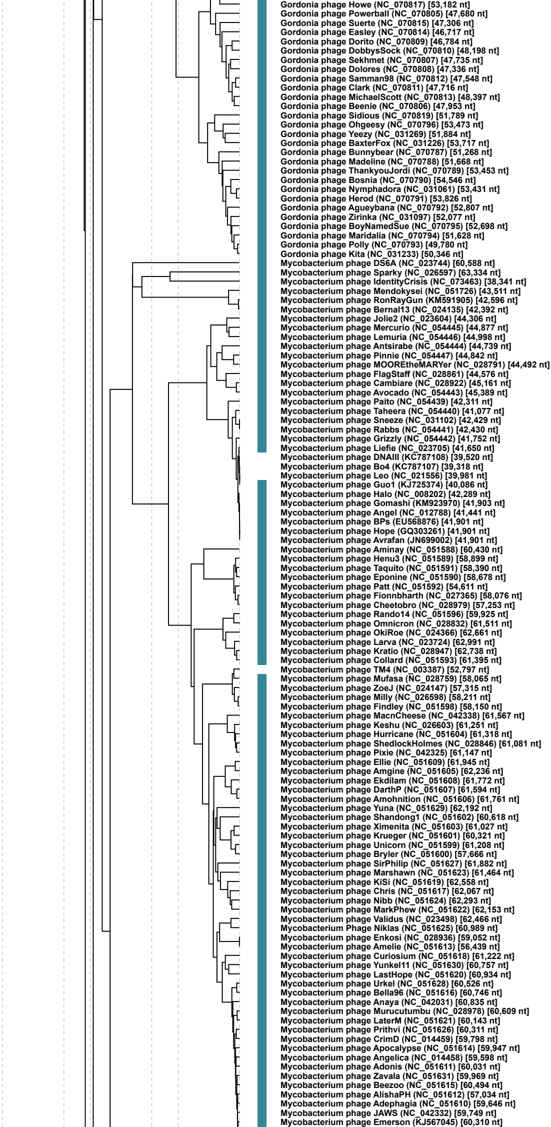

Gordonia phage Howe (NC_070817) [53,182 nt]
Gordonia phage Powerball (NC_070805) [47,680 nt]
Gordonia phage Suerte (NC_070815) [47,306 nt]
Gordonia phage Easley (NC_070814) [46,717 nt]
Gordonia phage Dorito (NC_070809) [46,784 nt]
Gordonia phage DobbysSock (NC_070810) [48,198 nt]
Gordonia phage Sekhmet (NC_070807) [47,735 nt]
Gordonia phage Dolores (NC_070808) [47,336 nt]
Gordonia phage Samman98 (NC_070812) [47,548 nt]
Gordonia phage Clark (NC_070811) [47,716 nt]
Gordonia phage MichaelScott (NC_070813) [48,397 nt]
Gordonia phage Beenie (NC_070806) [47,953 nt]
Gordonia phage Sidious (NC_070819) [51,789 nt]
Gordonia phage Ohgeesy (NC_070796) [53,473 nt]
Gordonia phage Yeezy (NC_031269) [51,884 nt]
Gordonia phage BaxterFox (NC_031226) [53,717 nt]
Gordonia phage Bunnybear (NC_070787) [51,268 nt]
Gordonia phage Madeline (NC_070788) [51,668 nt]
Gordonia phage ThankyouJordi (NC_070789) [53,453 nt]
Gordonia phage Bosnia (NC_070790) [54,546 nt]
Gordonia phage Nymphadora (NC_031061) [53,431 nt]
Gordonia phage Herod (NC_070791) [53,826 nt]
Gordonia phage Agueybana (NC_070792) [52,807 nt]
Gordonia phage Zirinka (NC_031097) [52,077 nt]
Gordonia phage BoyNamedSue (NC_070795) [52,698 nt]
Gordonia phage Maridalia (NC_070794) [51,628 nt]
Gordonia phage Polly (NC_070793) [49,780 nt]
Gordonia phage Kita (NC_031233) [50,346 nt]
Mycobacterium phage DS6A (NC_023744) [60,588 nt]
Mycobacterium phage Sparky (NC_026597) [63,334 nt]
Mycobacterium phage IdentityCrisis (NC_073463) [38,341 nt]
Mycobacterium phage Mendokysei (NC_051726) [43,511 nt]
Mycobacterium phage RonRayGun (KM591905) [42,596 nt]
Mycobacterium phage Bernal13 (NC_024135) [42,392 nt]
Mycobacterium phage Jolie2 (NC_023604) [44,306 nt]
Mycobacterium phage Mercurio (NC_054445) [44,877 nt]
Mycobacterium phage Lemuria (NC_054446) [44,998 nt]
Mycobacterium phage Antsirabe (NC_054444) [44,739 nt]
Mycobacterium phage Pinnie (NC_054447) [44,842 nt]
Mycobacterium phage MOOREtheMARYer (NC_028791) [44,492 nt]
Mycobacterium phage FlagStaff (NC_028861) [44,576 nt]
Mycobacterium phage Cambiare (NC_028922) [45,161 nt]
Mycobacterium phage Avocado (NC_054443) [45,389 nt]
Mycobacterium phage Paito (NC_054439) [42,311 nt]
Mycobacterium phage Taheera (NC_054440) [41,077 nt]
Mycobacterium phage Sneeze (NC_031102) [42,429 nt]
Mycobacterium phage Rabbs (NC_054441) [42,430 nt]
Mycobacterium phage Grizzly (NC_054442) [41,752 nt]
Mycobacterium phage Liefie (NC_023705) [41,650 nt]
Mycobacterium phage DNAIII (KC787108) [39,520 nt]
Mycobacterium phage Bo4 (KC787107) [39,318 nt]
Mycobacterium phage Leo (NC_021556) [39,981 nt]
Mycobacterium phage Guo1 (KJ725374) [40,086 nt]
Mycobacterium phage Halo (NC_008202) [42,289 nt]
Mycobacterium phage Gomashi (KM923970) [41,903 nt]
Mycobacterium phage Angel (NC_012788) [41,441 nt]
Mycobacterium phage BPs (EU568876) [41,901 nt]
Mycobacterium phage Hope (GQ303261) [41,901 nt]
Mycobacterium phage Avrafan (JN699002) [41,901 nt]
Mycobacterium phage Aminay (NC_051588) [60,430 nt]
Mycobacterium phage Henu3 (NC_051589) [58,899 nt]
Mycobacterium phage Taquito (NC_051591) [58,390 nt]
Mycobacterium phage Eponine (NC_051590) [58,678 nt]
Mycobacterium phage Patt (NC_051592) [54,611 nt]
Mycobacterium phage Fionnbharth (NC_027365) [58,076 nt]
Mycobacterium phage Cheetobro (NC_028979) [57,253 nt]
Mycobacterium phage Rando14 (NC_051596) [59,925 nt]
Mycobacterium phage Omnicron (NC_028832) [61,511 nt]
Mycobacterium phage OkiRoe (NC_024366) [62,661 nt]
Mycobacterium phage Larva (NC_023724) [62,991 nt]
Mycobacterium phage Kratio (NC_028947) [62,738 nt]
Mycobacterium phage Collard (NC_051593) [61,395 nt]
Mycobacterium phage TM4 (NC_003387) [52,797 nt]
Mycobacterium phage Mufasa (NC_028759) [58,065 nt]
Mycobacterium phage ZoeJ (NC_024147) [57,315 nt]
Mycobacterium phage Milly (NC_026598) [58,211 nt]
Mycobacterium phage Findley (NC_051598) [58,150 nt]
Mycobacterium phage MacnCheese (NC_042338) [61,567 nt]
Mycobacterium phage Keshu (NC_026603) [61,251 nt]
Mycobacterium phage Hurricane (NC_051604) [61,318 nt]
Mycobacterium phage ShedlockHolmes (NC_028846) [61,081 nt]
Mycobacterium phage Pixie (NC_042325) [61,147 nt]
Mycobacterium phage Ellie (NC_051609) [61,945 nt]
Mycobacterium phage Amgine (NC_051605) [62,236 nt]
Mycobacterium phage Ekdilam (NC_051608) [61,772 nt]
Mycobacterium phage DarthP (NC_051607) [61,594 nt]
Mycobacterium phage Amohnition (NC_051606) [61,761 nt]
Mycobacterium phage Yuna (NC_051629) [62,192 nt]
Mycobacterium phage Shandong1 (NC_051602) [60,618 nt]
Mycobacterium phage Ximenita (NC_051603) [61,027 nt]
Mycobacterium phage Krueger (NC_051601) [60,321 nt]
Mycobacterium phage Unicorn (NC_051599) [61,208 nt]
Mycobacterium phage Bryler (NC_051600) [57,666 nt]
Mycobacterium phage SirPhilip (NC_051627) [61,882 nt]
Mycobacterium phage Marshawn (NC_051623) [61,464 nt]
Mycobacterium phage KiSi (NC_051619) [62,558 nt]
Mycobacterium phage Chris (NC_051617) [62,067 nt]
Mycobacterium phage Nibb (NC_051624) [62,293 nt]
Mycobacterium phage MarkPhew (NC_051622) [62,153 nt]
Mycobacterium phage Validus (NC_023498) [62,466 nt]
Mycobacterium Phage Niklas (NC_051625) [60,989 nt]
Mycobacterium phage Enkosi (NC_028936) [59,052 nt]
Mycobacterium phage Amelie (NC_051613) [56,439 nt]
Mycobacterium phage Curiosium (NC_051618) [61,222 nt]
Mycobacterium phage Yunkel11 (NC_051630) [60,757 nt]
Mycobacterium phage LastHope (NC_051620) [60,934 nt]
Mycobacterium phage Urkel (NC_051628) [60,526 nt]
Mycobacterium phage Bella96 (NC_051616) [60,746 nt]
Mycobacterium phage Anaya (NC_042031) [60,835 nt]
Mycobacterium phage Murucutumbu (NC_028978) [60,609 nt]
Mycobacterium phage LaterM (NC_051621) [60,143 nt]
Mycobacterium phage Prithvi (NC_051626) [60,311 nt]
Mycobacterium phage CrimD (NC_014459) [59,798 nt]
Mycobacterium phage Apocalypse (NC_051614) [59,947 nt]
Mycobacterium phage Angelica (NC_014458) [59,598 nt]
Mycobacterium phage Adonis (NC_051611) [60,031 nt]
Mycobacterium phage Zavala (NC_051631) [59,969 nt]
Mycobacterium phage Beezoo (NC_051615) [60,494 nt]
Mycobacterium phage AlishaPH (NC_051612) [57,034 nt]
Mycobacterium phage Adephagia (NC_051610) [59,646 nt]
Mycobacterium phage JAWS (NC_042332) [59,749 nt]
Mycobacterium phage Emerson (KJ567045) [60,310 nt]

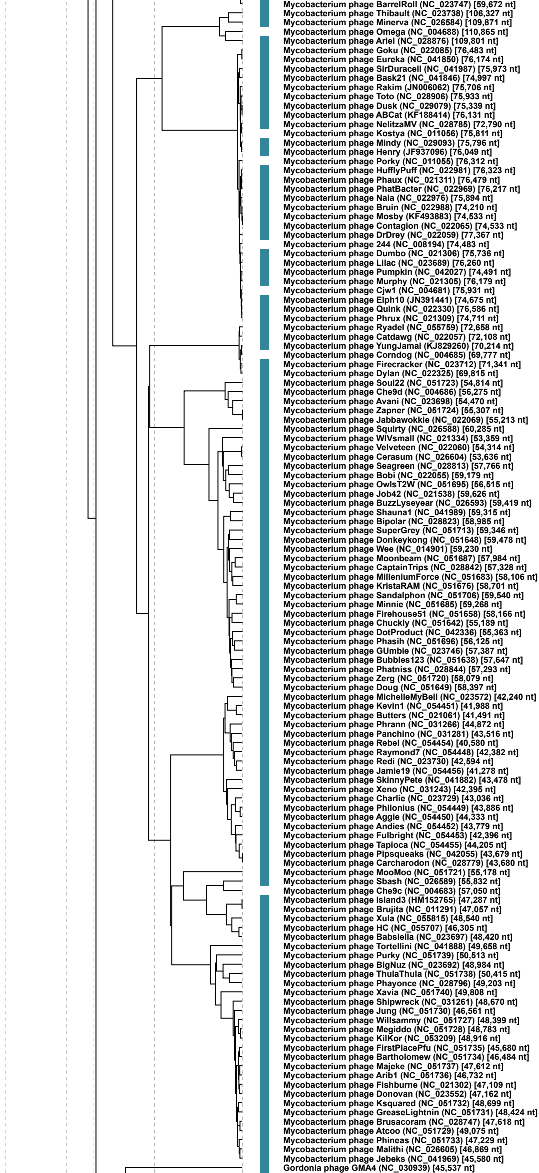

Mycobacterium phage BarrelRoll (NC_023747) [59,672 nt]
Mycobacterium phage Thibault (NC_023738) [106,327 nt]
Mycobacterium phage Minerva (NC_026584) [109,871 nt]
Mycobacterium phage Omega (NC_004688) [110,865 nt]
Mycobacterium phage Ariel (NC_028876) [109,801 nt]
Mycobacterium phage Goku (NC_022085) [76,483 nt]
Mycobacterium phage Eureka (NC_041850) [76,174 nt]
Mycobacterium phage SirDuracell (NC_041987) [75,973 nt]
Mycobacterium phage Bask21 (NC_041846) [74,997 nt]
Mycobacterium phage Rakim (JN006062) [75,706 nt]
Mycobacterium phage Toto (NC_028906) [75,933 nt]
Mycobacterium phage Dusk (NC_029079) [75,339 nt]
Mycobacterium phage ABCat (KF188414) [76,131 nt]
Mycobacterium phage NelitzaMV (NC_028785) [72,790 nt]
Mycobacterium phage Kostya (NC_011056) [75,811 nt]
Mycobacterium phage Mindy (NC_029093) [75,796 nt]
Mycobacterium phage Henry (JF937096) [76,049 nt]
Mycobacterium phage Porky (NC_011055) [76,312 nt]
Mycobacterium phage HufflyPuff (NC_022981) [76,323 nt]
Mycobacterium phage Phaux (NC_021311) [76,479 nt]
Mycobacterium phage PhatBacter (NC_022969) [76,217 nt]
Mycobacterium phage Nala (NC_022976) [75,894 nt]
Mycobacterium phage Bruin (NC_022988) [74,210 nt]
Mycobacterium phage Mosby (KF493883) [74,533 nt]
Mycobacterium phage Contagion (NC_022065) [74,533 nt]
Mycobacterium phage DrDrey (NC_022059) [77,367 nt]
Mycobacterium phage 244 (NC_008194) [74,483 nt]
Mycobacterium phage Dumbo (NC_021306) [75,736 nt]
Mycobacterium phage Lilac (NC_023689) [76,260 nt]
Mycobacterium phage Pumpkin (NC_042027) [74,491 nt]
Mycobacterium phage Murphy (NC_021305) [76,179 nt]
Mycobacterium phage Cjw1 (NC_004681) [75,931 nt]
Mycobacterium phage Elph10 (JN391441) [74,675 nt]
Mycobacterium phage Quink (NC_022330) [76,586 nt]
Mycobacterium phage Phrux (NC_021309) [74,711 nt]
Mycobacterium phage Ryadel (NC_055759) [72,658 nt]
Mycobacterium phage Catdawg (NC_022057) [72,108 nt]
Mycobacterium phage YungJamal (KJ829260) [70,214 nt]
Mycobacterium phage Corndog (NC_004685) [69,777 nt]
Mycobacterium phage Firecracker (NC_023712) [71,341 nt]
Mycobacterium phage Dylan (NC_022325) [69,815 nt]
Mycobacterium phage Soul22 (NC_051723) [54,814 nt]
Mycobacterium phage Che9d (NC_004686) [56,275 nt]
Mycobacterium phage Avani (NC_023698) [54,470 nt]
Mycobacterium phage Zapner (NC_051724) [55,307 nt]
Mycobacterium phage Jabbawokkie (NC_022069) [55,213 nt]
Mycobacterium phage Squirty (NC_026588) [60,285 nt]
Mycobacterium phage WIVsmall (NC_021334) [53,359 nt]
Mycobacterium phage Velveteen (NC_022060) [54,314 nt]
Mycobacterium phage Cerasum (NC_026604) [53,636 nt]
Mycobacterium phage Seagreen (NC_028813) [57,766 nt]
Mycobacterium phage Bobi (NC_022055) [59,179 nt]
Mycobacterium phage OwlsT2W (NC_051695) [56,515 nt]
Mycobacterium phage Job42 (NC_021538) [59,626 nt]
Mycobacterium phage BuzzLyseyear (NC_026593) [59,419 nt]
Mycobacterium phage Shauna1 (NC_041989) [59,315 nt]
Mycobacterium phage Bipolar (NC_028823) [58,985 nt]
Mycobacterium phage SuperGrey (NC_051713) [59,346 nt]
Mycobacterium phage Donkeykong (NC_051648) [59,478 nt]
Mycobacterium phage Wee (NC_014901) [59,230 nt]
Mycobacterium phage Moonbeam (NC_051687) [57,984 nt]
Mycobacterium phage CaptainTrips (NC_028842) [57,328 nt]
Mycobacterium phage MilleniumForce (NC_051683) [58,106 nt]
Mycobacterium phage KristaRAM (NC_051676) [58,701 nt]
Mycobacterium phage Sandalphon (NC_051706) [59,540 nt]
Mycobacterium phage Minnie (NC_051685) [59,268 nt]
Mycobacterium phage Firehouse51 (NC_051658) [58,166 nt]
Mycobacterium phage Chuckly (NC_051642) [55,189 nt]
Mycobacterium phage DotProduct (NC_042336) [55,363 nt]
Mycobacterium phage Phasih (NC_051696) [56,125 nt]
Mycobacterium phage GUmbie (NC_023746) [57,387 nt]
Mycobacterium phage Bubbles123 (NC_051638) [57,647 nt]
Mycobacterium phage Phatniss (NC_028844) [57,293 nt]
Mycobacterium phage Zerg (NC_051720) [58,079 nt]
Mycobacterium phage Doug (NC_051649) [58,397 nt]
Mycobacterium phage MichelleMyBell (NC_023572) [42,240 nt]
Mycobacterium phage Kevin1 (NC_054451) [41,988 nt]
Mycobacterium phage Butters (NC_021061) [41,491 nt]
Mycobacterium phage Phrann (NC_031266) [44,872 nt]
Mycobacterium phage Panchino (NC_031281) [43,516 nt]
Mycobacterium phage Rebel (NC_054454) [40,580 nt]
Mycobacterium phage Raymond7 (NC_054448) [42,382 nt]
Mycobacterium phage Redi (NC_023730) [42,594 nt]
Mycobacterium phage Jamie19 (NC_054456) [41,278 nt]
Mycobacterium phage SkinnyPete (NC_041882) [43,478 nt]
Mycobacterium phage Xeno (NC_031243) [42,395 nt]
Mycobacterium phage Charlie (NC_023729) [43,036 nt]
Mycobacterium phage Philonius (NC_054449) [43,886 nt]
Mycobacterium phage Aggie (NC_054450) [44,333 nt]
Mycobacterium phage Andies (NC_054452) [43,779 nt]
Mycobacterium phage Fulbright (NC_054453) [42,396 nt]
Mycobacterium phage Tapioca (NC_054455) [44,205 nt]
Mycobacterium phage Pipsqueaks (NC_042055) [43,679 nt]
Mycobacterium phage Carcharodon (NC_028779) [43,680 nt]
Mycobacterium phage MooMoo (NC_051721) [55,178 nt]
Mycobacterium phage Sbash (NC_026589) [55,832 nt]
Mycobacterium phage Che9c (NC_004683) [57,050 nt]
Mycobacterium phage Island3 (HM152765) [47,287 nt]
Mycobacterium phage Brujita (NC_011291) [47,057 nt]
Mycobacterium phage Xula (NC_055815) [48,540 nt]
Mycobacterium phage HC (NC_055707) [46,305 nt]
Mycobacterium phage Babsiella (NC_023697) [48,420 nt]
Mycobacterium phage Tortellini (NC_041888) [49,658 nt]
Mycobacterium phage Purky (NC_051739) [50,513 nt]
Mycobacterium phage BigNuz (NC_023692) [48,984 nt]
Mycobacterium phage ThulaThula (NC_051738) [50,415 nt]
Mycobacterium phage Phayonce (NC_028796) [49,203 nt]
Mycobacterium phage Xavia (NC_051740) [49,808 nt]
Mycobacterium phage Shipwreck (NC_031261) [48,670 nt]
Mycobacterium phage Jung (NC_051730) [46,561 nt]
Mycobacterium phage Willsammy (NC_051727) [48,399 nt]
Mycobacterium phage Megiddo (NC_051728) [48,783 nt]
Mycobacterium phage KilKor (NC_053209) [48,916 nt]
Mycobacterium phage FirstPlacePfu (NC_051735) [45,680 nt]
Mycobacterium phage Bartholomew (NC_051734) [46,484 nt]
Mycobacterium phage Majeke (NC_051737) [47,612 nt]
Mycobacterium phage Arib1 (NC_051736) [46,732 nt]
Mycobacterium phage Fishburne (NC_021302) [47,109 nt]
Mycobacterium phage Donovan (NC_023552) [47,162 nt]
Mycobacterium phage Ksquared (NC_051732) [48,699 nt]
Mycobacterium phage GreaseLightnin (NC_051731) [48,424 nt]
Mycobacterium phage Brusacoram (NC_028747) [47,618 nt]
Mycobacterium phage Atcoo (NC_051729) [49,075 nt]
Mycobacterium phage Phineas (NC_051733) [47,229 nt]
Mycobacterium phage Malithi (NC_026605) [46,869 nt]
Mycobacterium phage Jebeks (NC_041969) [45,580 nt]
Gordonia phage GMA4 (NC_030939) [45,537 nt]

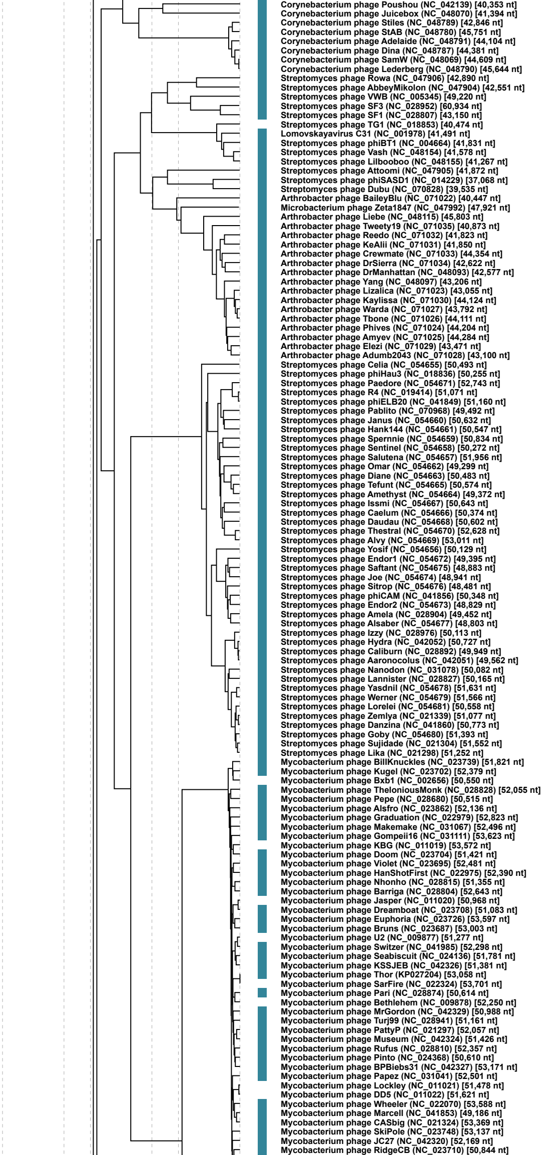

Corynebacterium phage Poushou (NC_042139) [40,353 nt]
Corynebacterium phage Juicebox (NC_048070) [41,394 nt]
Corynebacterium phage Stiles (NC_048789) [42,846 nt]
Corynebacterium phage StAB (NC_048780) [45,751 nt]
Corynebacterium phage Adelaide (NC_048791) [44,104 nt]
Corynebacterium phage Dina (NC_048787) [44,381 nt]
Corynebacterium phage SamW (NC_048069) [44,609 nt]
Corynebacterium phage Lederberg (NC_048790) [45,644 nt]
Streptomyces phage Rowa (NC_047906) [42,890 nt]
Streptomyces phage AbbeyMikolon (NC_047904) [42,551 nt]
Streptomyces phage VWB (NC_005345) [49,220 nt]
Streptomyces phage SF3 (NC_028952) [60,934 nt]
Streptomyces phage SF1 (NC_028807) [43,150 nt]
Streptomyces phage TG1 (NC_018853) [40,474 nt]
Lomovskayavirus C31 (NC_001978) [41,491 nt]
Streptomyces phage phiBT1 (NC_004664) [41,831 nt]
Streptomyces phage Vash (NC_048154) [41,578 nt]
Streptomyces phage Lilbooboo (NC_048155) [41,267 nt]
Streptomyces phage Attoomi (NC_047905) [41,872 nt]
Streptomyces phage phiSASD1 (NC_014229) [37,068 nt]
Streptomyces phage Dubu (NC_070828) [39,535 nt]
Arthrobacter phage BaileyBlu (NC_071022) [40,447 nt]
Microbacterium phage Zeta1847 (NC_047992) [47,921 nt]
Arthrobacter phage Liebe (NC_048115) [45,803 nt]
Arthrobacter phage Tweety19 (NC_071035) [40,873 nt]
Arthrobacter phage Reedo (NC_071032) [41,823 nt]
Arthrobacter phage KeAlii (NC_071031) [41,850 nt]
Arthrobacter phage Crewmate (NC_071033) [44,354 nt]
Arthrobacter phage DrSierra (NC_071034) [42,622 nt]
Arthrobacter phage DrManhattan (NC_048093) [42,577 nt]
Arthrobacter phage Yang (NC_048097) [43,206 nt]
Arthrobacter phage Lizalica (NC_071023) [43,055 nt]
Arthrobacter phage Kaylissa (NC_071030) [44,124 nt]
Arthrobacter phage Warda (NC_071027) [43,792 nt]
Arthrobacter phage Tbone (NC_071026) [44,111 nt]
Arthrobacter phage Phives (NC_071024) [44,204 nt]
Arthrobacter phage Amyev (NC_071025) [44,284 nt]
Arthrobacter phage Elezi (NC_071029) [43,471 nt]
Arthrobacter phage Adumb2043 (NC_071028) [43,100 nt]
Streptomyces phage Celia (NC_054655) [50,493 nt]
Streptomyces phage phiHau3 (NC_018836) [50,255 nt]
Streptomyces phage Paedore (NC_054671) [52,743 nt]
Streptomyces phage R4 (NC_019414) [51,071 nt]
Streptomyces phage phiELB20 (NC_041849) [51,160 nt]
Streptomyces phage Pablito (NC_070968) [49,492 nt]
Streptomyces phage Janus (NC_054660) [50,632 nt]
Streptomyces phage Hank144 (NC_054661) [50,547 nt]
Streptomyces phage Spernnie (NC_054659) [50,834 nt]
Streptomyces phage Sentinel (NC_054658) [50,272 nt]
Streptomyces phage Salutena (NC_054657) [51,956 nt]
Streptomyces phage Omar (NC_054662) [49,299 nt]
Streptomyces phage Diane (NC_054663) [50,483 nt]
Streptomyces phage Tefunt (NC_054665) [50,574 nt]
Streptomyces phage Amethyst (NC_054664) [49,372 nt]
Streptomyces phage Issmi (NC_054667) [50,643 nt]
Streptomyces phage Caelum (NC_054666) [50,374 nt]
Streptomyces phage Daudau (NC_054668) [50,602 nt]
Streptomyces phage Thestral (NC_054670) [52,628 nt]
Streptomyces phage Alvy (NC_054669) [53,011 nt]
Streptomyces phage Yosif (NC_054656) [50,129 nt]
Streptomyces phage Endor1 (NC_054672) [49,395 nt]
Streptomyces phage Saftant (NC_054675) [48,883 nt]
Streptomyces phage Joe (NC_054674) [48,941 nt]
Streptomyces phage Sitrop (NC_054676) [48,481 nt]
Streptomyces phage phiCAM (NC_041856) [50,348 nt]
Streptomyces phage Endor2 (NC_054673) [48,829 nt]
Streptomyces phage Amela (NC_028904) [49,452 nt]
Streptomyces phage Alsaber (NC_054677) [48,803 nt]
Streptomyces phage Izzy (NC_028976) [50,113 nt]
Streptomyces phage Hydra (NC_042052) [50,727 nt]
Streptomyces phage Caliburn (NC_028892) [49,949 nt]
Streptomyces phage Aaronocolus (NC_042051) [49,562 nt]
Streptomyces phage Nanodon (NC_031078) [50,082 nt]
Streptomyces phage Lannister (NC_028827) [50,165 nt]
Streptomyces phage Yasdnil (NC_054678) [51,631 nt]
Streptomyces phage Werner (NC_054679) [51,566 nt]
Streptomyces phage Lorelei (NC_054681) [50,558 nt]
Streptomyces phage Zemlya (NC_021339) [51,077 nt]
Streptomyces phage Danzina (NC_041860) [50,773 nt]
Streptomyces phage Goby (NC_054680) [51,393 nt]
Streptomyces phage Sujidade (NC_021304) [51,552 nt]
Streptomyces phage Lika (NC_021298) [51,252 nt]
Mycobacterium phage BillKnuckles (NC_023739) [51,821 nt]
Mycobacterium phage Kugel (NC_023702) [52,379 nt]
Mycobacterium phage Bxb1 (NC_002656) [50,550 nt]
Mycobacterium phage TheloniousMonk (NC_028828) [52,055 nt]
Mycobacterium phage Pepe (NC_028680) [50,515 nt]
Mycobacterium phage Alsfro (NC_023862) [52,136 nt]
Mycobacterium phage Graduation (NC_022979) [52,823 nt]
Mycobacterium phage Makemake (NC_031067) [52,496 nt]
Mycobacterium phage Gompeii16 (NC_031111) [53,623 nt]
Mycobacterium phage KBG (NC_011019) [53,572 nt]
Mycobacterium phage Doom (NC_023704) [51,421 nt]
Mycobacterium phage Violet (NC_023695) [52,481 nt]
Mycobacterium phage HanShotFirst (NC_022975) [52,390 nt]
Mycobacterium phage Nhonho (NC_028815) [51,355 nt]
Mycobacterium phage Barriga (NC_028804) [52,643 nt]
Mycobacterium phage Jasper (NC_011020) [50,968 nt]
Mycobacterium phage Dreamboat (NC_023708) [51,083 nt]
Mycobacterium phage Euphoria (NC_023726) [53,597 nt]
Mycobacterium phage Bruns (NC_023687) [53,003 nt]
Mycobacterium phage U2 (NC_009877) [51,277 nt]
Mycobacterium phage Switzer (NC_041985) [52,298 nt]
Mycobacterium phage Seabiscuit (NC_024136) [51,781 nt]
Mycobacterium phage KSSJEB (NC_042326) [51,381 nt]
Mycobacterium phage Thor (KP027204) [53,058 nt]
Mycobacterium phage SarFire (NC_022324) [53,701 nt]
Mycobacterium phage Pari (NC_028874) [50,614 nt]
Mycobacterium phage Bethlehem (NC_009878) [52,250 nt]
Mycobacterium phage MrGordon (NC_042329) [50,988 nt]
Mycobacterium phage Turj99 (NC_028941) [51,161 nt]
Mycobacterium phage PattyP (NC_021297) [52,057 nt]
Mycobacterium phage Museum (NC_042324) [51,426 nt]
Mycobacterium phage Rufus (NC_028810) [52,357 nt]
Mycobacterium phage Pinto (NC_024368) [50,610 nt]
Mycobacterium phage BPBiebs31 (NC_042327) [53,171 nt]
Mycobacterium phage Papez (NC_031041) [52,501 nt]
Mycobacterium phage Lockley (NC_011021) [51,478 nt]
Mycobacterium phage DD5 (NC_011022) [51,621 nt]
Mycobacterium phage Wheeler (NC_022070) [53,588 nt]
Mycobacterium phage Marcell (NC_041853) [49,186 nt]
Mycobacterium phage CASbig (NC_021324) [53,369 nt]
Mycobacterium phage SkiPole (NC_023748) [53,137 nt]
Mycobacterium phage JC27 (NC_042320) [52,169 nt]
Mycobacterium phage RidgeCB (NC_023710) [50,844 nt]

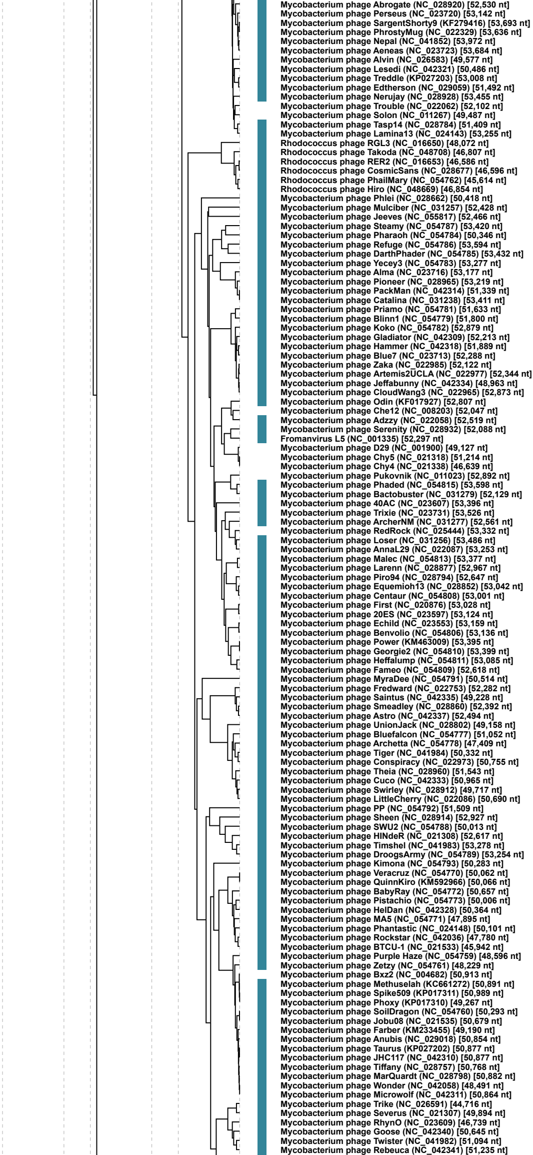
Mycobacterium phage Abrogate (NC_028920) [52,530 nt]
Mycobacterium phage Perseus (NC_023720) [53,142 nt]
Mycobacterium phage SargentShorty9 (KF279416) [53,693 nt]
Mycobacterium phage PhrostyMug (NC_022329) [53,636 nt]
Mycobacterium phage Nepal (NC_041852) [53,972 nt]
Mycobacterium phage Aeneas (NC_023723) [53,684 nt]
Mycobacterium phage Alvin (NC_026583) [49,577 nt]
Mycobacterium phage Lesedi (NC_042321) [50,486 nt]
Mycobacterium phage Treddle (KP027203) [53,008 nt]
Mycobacterium phage Edtherson (NC_029059) [51,492 nt]
Mycobacterium phage Nerujay (NC_028928) [53,455 nt]
Mycobacterium phage Trouble (NC_022062) [52,102 nt]
Mycobacterium phage Solon (NC_011267) [49,487 nt]
Mycobacterium phage Tasp14 (NC_028784) [51,409 nt]
Mycobacterium phage Lamina13 (NC_024143) [53,255 nt]
Rhodococcus phage RGL3 (NC_016650) [48,072 nt]
Rhodococcus phage Takoda (NC_048708) [46,807 nt]
Rhodococcus phage RER2 (NC_016653) [46,586 nt]
Rhodococcus phage CosmicSans (NC_028677) [46,596 nt]
Rhodococcus phage PhailMary (NC_054762) [45,614 nt]
Rhodococcus phage Hiro (NC_048669) [46,854 nt]
Mycobacterium phage Phlei (NC_028662) [50,418 nt]
Mycobacterium phage Mulciber (NC_031257) [52,428 nt]
Mycobacterium phage Jeeves (NC_055817) [52,466 nt]
Mycobacterium phage Steamy (NC_054787) [53,420 nt]
Mycobacterium phage Pharaoh (NC_054784) [50,346 nt]
Mycobacterium phage Refuge (NC_054786) [53,594 nt]
Mycobacterium phage DarthPhader (NC_054785) [53,432 nt]
Mycobacterium phage Yecey3 (NC_054783) [53,277 nt]
Mycobacterium phage Alma (NC_023716) [53,177 nt]
Mycobacterium phage Pioneer (NC_028965) [53,219 nt]
Mycobacterium phage PackMan (NC_042314) [51,339 nt]
Mycobacterium phage Catalina (NC_031238) [53,411 nt]
Mycobacterium phage Priamo (NC_054781) [51,633 nt]
Mycobacterium phage Blinn1 (NC_054779) [51,800 nt]
Mycobacterium phage Koko (NC_054782) [52,879 nt]
Mycobacterium phage Gladiator (NC_042309) [52,213 nt]
Mycobacterium phage Hammer (NC_042318) [51,889 nt]
Mycobacterium phage Blue7 (NC_023713) [52,288 nt]
Mycobacterium phage Zaka (NC_022985) [52,122 nt]
Mycobacterium phage Artemis2UCLA (NC_022977) [52,344 nt]
Mycobacterium phage Jeffabunny (NC_042334) [48,963 nt]
Mycobacterium phage CloudWang3 (NC_022965) [52,873 nt]
Mycobacterium phage Odin (KF017927) [52,807 nt]
Mycobacterium phage Che12 (NC_008203) [52,047 nt]
Mycobacterium phage Adzzy (NC_022058) [52,519 nt]
Mycobacterium phage Serenity (NC_028932) [52,088 nt]
Fromanvirus L5 (NC_001335) [52,297 nt]
Mycobacterium phage D29 (NC_001900) [49,127 nt]
Mycobacterium phage Chy5 (NC_021318) [51,214 nt]
Mycobacterium phage Chy4 (NC_021338) [46,639 nt]
Mycobacterium phage Pukovnik (NC_011023) [52,892 nt]
Mycobacterium phage Phaded (NC_054815) [53,598 nt]
Mycobacterium phage Bactobuster (NC_031279) [52,129 nt]
Mycobacterium phage 40AC (NC_023607) [53,396 nt]
Mycobacterium phage Trixie (NC_023731) [53,526 nt]
Mycobacterium phage ArcherNM (NC_031277) [52,561 nt]
Mycobacterium phage RedRock (NC_025444) [53,332 nt]
Mycobacterium phage Loser (NC_031256) [53,486 nt]
Mycobacterium phage AnnaL29 (NC_022087) [53,253 nt]
Mycobacterium phage Malec (NC_054813) [53,377 nt]
Mycobacterium phage Larenn (NC_028877) [52,967 nt]
Mycobacterium phage Piro94 (NC_028794) [52,647 nt]
Mycobacterium phage Equemioh13 (NC_028852) [53,042 nt]
Mycobacterium phage Centaur (NC_054808) [53,001 nt]
Mycobacterium phage First (NC_020876) [53,028 nt]
Mycobacterium phage 20ES (NC_023597) [53,124 nt]
Mycobacterium phage Echild (NC_023553) [53,159 nt]
Mycobacterium phage Benvolio (NC_054806) [53,136 nt]
Mycobacterium phage Power (KM463009) [53,395 nt]
Mycobacterium phage Georgie2 (NC_054810) [53,399 nt]
Mycobacterium phage Heffalump (NC_054811) [53,085 nt]
Mycobacterium phage Fameo (NC_054809) [52,618 nt]
Mycobacterium phage MyraDee (NC_054791) [50,514 nt]
Mycobacterium phage Fredward (NC_022753) [52,282 nt]
Mycobacterium phage Saintus (NC_042335) [49,228 nt]
Mycobacterium phage Smeadley (NC_028860) [52,392 nt]
Mycobacterium phage Astro (NC_042337) [52,494 nt]
Mycobacterium phage UnionJack (NC_028802) [49,158 nt]
Mycobacterium phage Bluefalcon (NC_054777) [51,052 nt]
Mycobacterium phage Archetta (NC_054778) [47,409 nt]
Mycobacterium phage Tiger (NC_041984) [50,332 nt]
Mycobacterium phage Conspiracy (NC_022973) [50,755 nt]
Mycobacterium phage Theia (NC_028960) [51,543 nt]
Mycobacterium phage Cuco (NC_042333) [50,965 nt]
Mycobacterium phage Swirley (NC_028912) [49,717 nt]
Mycobacterium phage LittleCherry (NC_022086) [50,690 nt]
Mycobacterium phage PP (NC_054792) [51,509 nt]
Mycobacterium phage Sheen (NC_028914) [52,927 nt]
Mycobacterium phage SWU2 (NC_054788) [50,013 nt]
Mycobacterium phage HINdeR (NC_021308) [52,617 nt]
Mycobacterium phage Timshel (NC_041983) [53,278 nt]
Mycobacterium phage DroogsArmy (NC_054789) [53,254 nt]
Mycobacterium phage Kimona (NC_054793) [50,283 nt]
Mycobacterium phage Veracruz (NC_054770) [50,062 nt]
Mycobacterium phage QuinnKiro (KM592966) [50,066 nt]
Mycobacterium phage BabyRay (NC_054772) [50,657 nt]
Mycobacterium phage Pistachio (NC_054773) [50,006 nt]
Mycobacterium phage HelDan (NC_042328) [50,364 nt]
Mycobacterium phage MA5 (NC_054771) [47,895 nt]
Mycobacterium phage Phantastic (NC_024148) [50,101 nt]
Mycobacterium phage Rockstar (NC_042036) [47,780 nt]
Mycobacterium phage BTCU-1 (NC_021533) [45,942 nt]
Mycobacterium phage Purple Haze (NC_054759) [48,596 nt]
Mycobacterium phage Zetzy (NC_054761) [48,229 nt]
Mycobacterium phage Bxz2 (NC_004682) [50,913 nt]
Mycobacterium phage Methuselah (KC661272) [50,891 nt]
Mycobacterium phage Spike509 (KP017311) [50,989 nt]
Mycobacterium phage Phoxy (KP017310) [49,267 nt]
Mycobacterium phage SoilDragon (NC_054760) [50,293 nt]
Mycobacterium phage Jobu08 (NC_021535) [50,679 nt]
Mycobacterium phage Farber (KM233455) [49,190 nt]
Mycobacterium phage Anubis (NC_029018) [50,854 nt]
Mycobacterium phage Taurus (KP027202) [50,877 nt]
Mycobacterium phage JHC117 (NC_042310) [50,877 nt]
Mycobacterium phage Tiffany (NC_028757) [50,768 nt]
Mycobacterium phage MarQuardt (NC_028798) [50,882 nt]
Mycobacterium phage Wonder (NC_042058) [48,491 nt]
Mycobacterium phage Microwolf (NC_042311) [50,864 nt]
Mycobacterium phage Trike (NC_026591) [44,716 nt]
Mycobacterium phage Severus (NC_021307) [49,894 nt]
Mycobacterium phage RhynO (NC_023609) [46,739 nt]
Mycobacterium phage Goose (NC_042340) [50,645 nt]
Mycobacterium phage Twister (NC_041982) [51,094 nt]
Mycobacterium phage Rebeuca (NC_042341) [51,235 nt]

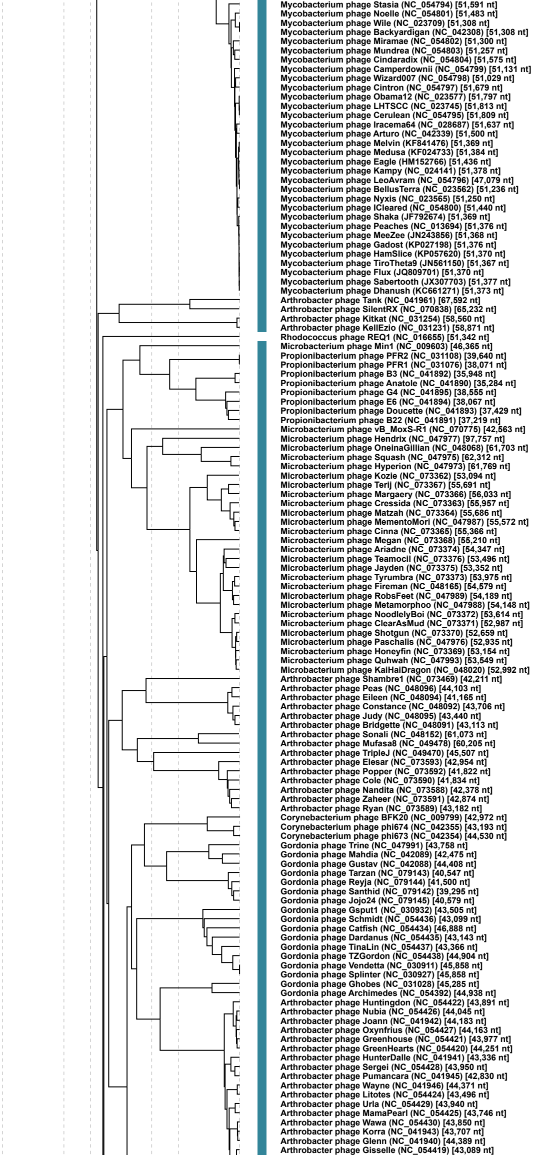

Mycobacterium phage Stasia (NC_054794) [51,591 nt]
Mycobacterium phage Noelle (NC_054801) [51,483 nt]
Mycobacterium phage Wile (NC_023709) [51,308 nt]
Mycobacterium phage Backyardigan (NC_042308) [51,308 nt]
Mycobacterium phage Miramae (NC_054802) [51,300 nt]
Mycobacterium phage Mundrea (NC_054803) [51,257 nt]
Mycobacterium phage Cindaradix (NC_054804) [51,575 nt]
Mycobacterium phage Camperdownii (NC_054799) [51,131 nt]
Mycobacterium phage Wizard007 (NC_054798) [51,029 nt]
Mycobacterium phage Cintron (NC_054797) [51,679 nt]
Mycobacterium phage Obama12 (NC_023577) [51,797 nt]
Mycobacterium phage LHTSCC (NC_023745) [51,813 nt]
Mycobacterium phage Cerulean (NC_054795) [51,809 nt]
Mycobacterium phage Iracema64 (NC_028687) [51,637 nt]
Mycobacterium phage Arturo (NC_042339) [51,500 nt]
Mycobacterium phage Melvin (KF841476) [51,369 nt]
Mycobacterium phage Medusa (KF024733) [51,384 nt]
Mycobacterium phage Eagle (HM152766) [51,436 nt]
Mycobacterium phage Kampy (NC_024141) [51,378 nt]
Mycobacterium phage LeoAvram (NC_054796) [47,079 nt]
Mycobacterium phage BellusTerra (NC_023562) [51,236 nt]
Mycobacterium phage Nyxis (NC_023565) [51,250 nt]
Mycobacterium phage ICleared (NC_054800) [51,440 nt]
Mycobacterium phage Shaka (JF792674) [51,369 nt]
Mycobacterium phage Peaches (NC_013694) [51,376 nt]
Mycobacterium phage MeeZee (JN243856) [51,368 nt]
Mycobacterium phage Gadost (KP027198) [51,376 nt]
Mycobacterium phage HamSlice (KP057620) [51,370 nt]
Mycobacterium phage TiroTheta9 (JN561150) [51,367 nt]
Mycobacterium phage Flux (JQ809701) [51,370 nt]
Mycobacterium phage Sabertooth (JX307703) [51,377 nt]
Mycobacterium phage Dhanush (KC661271) [51,373 nt]
Arthrobacter phage Tank (NC_041961) [67,592 nt]
Arthrobacter phage SilentRX (NC_070838) [65,232 nt]
Arthrobacter phage Kitkat (NC_031254) [58,560 nt]
Arthrobacter phage KellEzio (NC_031231) [58,871 nt]
Rhodococcus phage REQ1 (NC_016655) [51,342 nt]
Microbacterium phage Min1 (NC_009603) [46,365 nt]
Propionibacterium phage PFR2 (NC_031108) [39,640 nt]
Propionibacterium phage PFR1 (NC_031076) [38,071 nt]
Propionibacterium phage B3 (NC_041892) [35,948 nt]
Propionibacterium phage Anatole (NC_041890) [35,284 nt]
Propionibacterium phage G4 (NC_041895) [38,555 nt]
Propionibacterium phage E6 (NC_041894) [38,067 nt]
Propionibacterium phage Doucette (NC_041893) [37,429 nt]
Propionibacterium phage B22 (NC_041891) [37,219 nt]
Microbacterium phage vB_MoxS-R1 (NC_070775) [42,563 nt]
Microbacterium phage Hendrix (NC_047977) [97,757 nt]
Microbacterium phage OneinaGillian (NC_048068) [61,703 nt]
Microbacterium phage Squash (NC_047975) [62,312 nt]
Microbacterium phage Hyperion (NC_047973) [61,769 nt]
Microbacterium phage Kozie (NC_073362) [53,094 nt]
Microbacterium phage Terij (NC_073367) [55,691 nt]
Microbacterium phage Margaery (NC_073366) [56,033 nt]
Microbacterium phage Cressida (NC_073363) [55,957 nt]
Microbacterium phage Matzah (NC_073364) [55,686 nt]
Microbacterium phage MementoMori (NC_047987) [55,572 nt]
Microbacterium phage Cinna (NC_073365) [55,366 nt]
Microbacterium phage Megan (NC_073368) [55,210 nt]
Microbacterium phage Ariadne (NC_073374) [54,347 nt]
Microbacterium phage Teamocil (NC_073376) [53,496 nt]
Microbacterium phage Jayden (NC_073375) [53,352 nt]
Microbacterium phage Tyrumbra (NC_073373) [53,975 nt]
Microbacterium phage Fireman (NC_048165) [54,579 nt]
Microbacterium phage RobsFeet (NC_047989) [54,189 nt]
Microbacterium phage Metamorphoo (NC_047988) [54,148 nt]
Microbacterium phage NoodlelyBoi (NC_073372) [53,614 nt]
Microbacterium phage ClearAsMud (NC_073371) [52,987 nt]
Microbacterium phage Shotgun (NC_073370) [52,659 nt]
Microbacterium phage Paschalis (NC_047976) [52,935 nt]
Microbacterium phage Honeyfin (NC_073369) [53,154 nt]
Microbacterium phage Quhwah (NC_047993) [53,549 nt]
Microbacterium phage KaiHaiDragon (NC_048020) [52,992 nt]
Arthrobacter phage Shambre1 (NC_073469) [42,211 nt]
Arthrobacter phage Peas (NC_048096) [44,103 nt]
Arthrobacter phage Eileen (NC_048094) [41,165 nt]
Arthrobacter phage Constance (NC_048092) [43,706 nt]
Arthrobacter phage Judy (NC_048095) [43,440 nt]
Arthrobacter phage Bridgette (NC_048091) [43,113 nt]
Arthrobacter phage Sonali (NC_048152) [61,073 nt]
Arthrobacter phage Mufasa8 (NC_049478) [60,205 nt]
Arthrobacter phage TripleJ (NC_049470) [45,507 nt]
Arthrobacter phage Elesar (NC_073593) [42,954 nt]
Arthrobacter phage Popper (NC_073592) [41,822 nt]
Arthrobacter phage Cole (NC_073590) [41,834 nt]
Arthrobacter phage Nandita (NC_073588) [42,378 nt]
Arthrobacter phage Zaheer (NC_073591) [42,874 nt]
Arthrobacter phage Ryan (NC_073589) [43,182 nt]
Corynebacterium phage BFK20 (NC_009799) [42,972 nt]
Corynebacterium phage phi674 (NC_042355) [43,193 nt]
Corynebacterium phage phi673 (NC_042354) [44,530 nt]
Gordonia phage Trine (NC_047991) [43,758 nt]
Gordonia phage Mahdia (NC_042089) [42,475 nt]
Gordonia phage Gustav (NC_042088) [44,408 nt]
Gordonia phage Tarzan (NC_079143) [40,547 nt]
Gordonia phage Reyja (NC_079144) [41,500 nt]
Gordonia phage Santhid (NC_079142) [39,295 nt]
Gordonia phage Jojo24 (NC_079145) [40,579 nt]
Gordonia phage Gsput1 (NC_030932) [43,505 nt]
Gordonia phage Schmidt (NC_054436) [43,099 nt]
Gordonia phage Catfish (NC_054434) [46,888 nt]
Gordonia phage Dardanus (NC_054435) [43,143 nt]
Gordonia phage TinaLin (NC_054437) [43,366 nt]
Gordonia phage TZGordon (NC_054438) [44,904 nt]
Gordonia phage Vendetta (NC_030911) [45,858 nt]
Gordonia phage Splinter (NC_030927) [45,858 nt]
Gordonia phage Ghobes (NC_031028) [45,285 nt]
Gordonia phage Archimedes (NC_054392) [44,938 nt]
Arthrobacter phage Huntingdon (NC_054422) [43,891 nt]
Arthrobacter phage Nubia (NC_054426) [44,045 nt]
Arthrobacter phage Joann (NC_041942) [44,183 nt]
Arthrobacter phage Oxynfrius (NC_054427) [44,163 nt]
Arthrobacter phage Greenhouse (NC_054421) [43,977 nt]
Arthrobacter phage GreenHearts (NC_054420) [44,251 nt]
Arthrobacter phage HunterDalle (NC_041941) [43,336 nt]
Arthrobacter phage Sergei (NC_054428) [43,950 nt]
Arthrobacter phage Pumancara (NC_041945) [42,830 nt]
Arthrobacter phage Wayne (NC_041946) [44,371 nt]
Arthrobacter phage Litotes (NC_054424) [43,496 nt]
Arthrobacter phage Urla (NC_054429) [43,940 nt]
Arthrobacter phage MamaPearl (NC_054425) [43,746 nt]
Arthrobacter phage Wawa (NC_054430) [43,850 nt]
Arthrobacter phage Korra (NC_041943) [43,707 nt]
Arthrobacter phage Glenn (NC_041940) [44,389 nt]
Arthrobacter phage Gisselle (NC_054419) [43,089 nt]

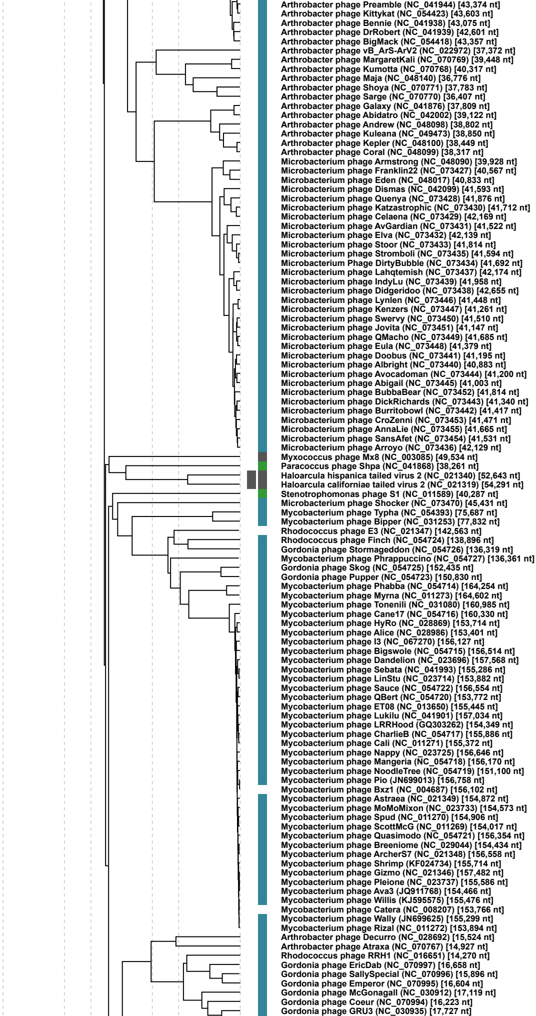

Arthrobacter phage Preamble (NC_041944) [43,374 nt]
Arthrobacter phage Kittykat (NC_054423) [43,603 nt]
Arthrobacter phage Bennie (NC_041938) [43,075 nt]
Arthrobacter phage DrRobert (NC_041939) [42,601 nt]
Arthrobacter phage BigMack (NC_054418) [43,357 nt]
Arthrobacter phage vB_ArS-ArV2 (NC_022972) [37,372 nt]
Arthrobacter phage MargaretKali (NC_070769) [39,448 nt]
Arthrobacter phage Kumotta (NC_070768) [40,317 nt]
Arthrobacter phage Maja (NC_048140) [36,776 nt]
Arthrobacter phage Shoya (NC_070771) [37,783 nt]
Arthrobacter phage Sarge (NC_070770) [36,407 nt]
Arthrobacter phage Galaxy (NC_041876) [37,809 nt]
Arthrobacter phage Abidatro (NC_042002) [39,122 nt]
Arthrobacter phage Andrew (NC_048098) [38,802 nt]
Arthrobacter phage Kuleana (NC_049473) [38,850 nt]
Arthrobacter phage Kepler (NC_048100) [38,449 nt]
Arthrobacter phage Coral (NC_048099) [38,317 nt]
Microbacterium phage Armstrong (NC_048090) [39,928 nt]
Microbacterium phage Franklin22 (NC_073427) [40,567 nt]
Microbacterium phage Eden (NC_048017) [40,833 nt]
Microbacterium phage Dismas (NC_042099) [41,593 nt]
Microbacterium phage Quenya (NC_073428) [41,876 nt]
Microbacterium phage Katzastrophic (NC_073430) [41,712 nt]
Microbacterium phage Celaena (NC_073429) [42,169 nt]
Microbacterium phage AvGardian (NC_073431) [41,522 nt]
Microbacterium phage Elva (NC_073432) [42,139 nt]
Microbacterium phage Stoor (NC_073433) [41,814 nt]
Microbacterium phage Stromboli (NC_073435) [41,594 nt]
Microbacterium Phage DirtyBubble (NC_073434) [41,692 nt]
Microbacterium phage Lahqtemish (NC_073437) [42,174 nt]
Microbacterium phage IndyLu (NC_073439) [41,958 nt]
Microbacterium phage Didgeridoo (NC_073438) [42,655 nt]
Microbacterium phage Lynlen (NC_073446) [41,448 nt]
Microbacterium phage Kenzers (NC_073447) [41,261 nt]
Microbacterium phage Swervy (NC_073450) [41,510 nt]
Microbacterium phage Jovita (NC_073451) [41,147 nt]
Microbacterium phage QMacho (NC_073449) [41,685 nt]
Microbacterium phage Eula (NC_073448) [41,379 nt]
Microbacterium phage Doobus (NC_073441) [41,195 nt]
Microbacterium phage Albright (NC_073440) [40,883 nt]
Microbacterium phage Avocadoman (NC_073444) [41,200 nt]
Microbacterium phage Abigail (NC_073445) [41,003 nt]
Microbacterium phage BubbaBear (NC_073452) [41,814 nt]
Microbacterium phage DickRichards (NC_073443) [41,340 nt]
Microbacterium phage Burritobowl (NC_073442) [41,417 nt]
Microbacterium phage CroZenni (NC_073453) [41,471 nt]
Microbacterium phage AnnaLie (NC_073455) [41,665 nt]
Microbacterium phage SansAfet (NC_073454) [41,531 nt]
Microbacterium phage Arroyo (NC_073436) [42,129 nt]
Myxococcus phage Mx8 (NC_003085) [49,534 nt]
Paracoccus phage Shpa (NC_041868) [38,261 nt]
Haloarcula hispanica tailed virus 2 (NC_021340) [52,643 nt]
Haloarcula californiae tailed virus 2 (NC_021319) [54,291 nt]
Stenotrophomonas phage S1 (NC_011589) [40,287 nt]
Microbacterium phage Shocker (NC_073470) [45,431 nt]
Mycobacterium phage Typha (NC_054393) [75,687 nt]
Mycobacterium phage Bipper (NC_031253) [77,832 nt]
Rhodococcus phage E3 (NC_021347) [142,563 nt]
Rhodococcus phage Finch (NC_054724) [138,896 nt]
Gordonia phage Stormageddon (NC_054726) [136,319 nt]
Mycobacterium phage Phrappuccino (NC_054727) [136,361 nt]
Gordonia phage Skog (NC_054725) [152,435 nt]
Gordonia phage Pupper (NC_054723) [150,830 nt]
Mycobacterium phage Phabba (NC_054714) [164,254 nt]
Mycobacterium phage Myrna (NC_011273) [164,602 nt]
Mycobacterium phage Tonenili (NC_031080) [160,985 nt]
Mycobacterium phage Cane17 (NC_054716) [160,330 nt]
Mycobacterium phage HyRo (NC_028869) [153,714 nt]
Mycobacterium phage Alice (NC_028986) [153,401 nt]
Mycobacterium phage I3 (NC_067270) [156,127 nt]
Mycobacterium phage Bigswole (NC_054715) [156,514 nt]
Mycobacterium phage Dandelion (NC_023696) [157,568 nt]
Mycobacterium phage Sebata (NC_041993) [155,286 nt]
Mycobacterium phage LinStu (NC_023714) [153,882 nt]
Mycobacterium phage Sauce (NC_054722) [156,554 nt]
Mycobacterium phage QBert (NC_054720) [153,772 nt]
Mycobacterium phage ET08 (NC_013650) [155,445 nt]
Mycobacterium phage Lukilu (NC_041901) [157,034 nt]
Mycobacterium phage LRRHood (GQ303262) [154,349 nt]
Mycobacterium phage CharlieB (NC_054717) [155,886 nt]
Mycobacterium phage Cali (NC_011271) [155,372 nt]
Mycobacterium phage Nappy (NC_023725) [156,646 nt]
Mycobacterium phage Mangeria (NC_054718) [156,170 nt]
Mycobacterium phage NoodleTree (NC_054719) [151,100 nt]
Mycobacterium phage Pio (JN699013) [156,758 nt]
Mycobacterium phage Bxz1 (NC_004687) [156,102 nt]
Mycobacterium phage Astraea (NC_021349) [154,872 nt]
Mycobacterium phage MoMoMixon (NC_023733) [154,573 nt]
Mycobacterium phage Spud (NC_011270) [154,906 nt]
Mycobacterium phage ScottMcG (NC_011269) [154,017 nt]
Mycobacterium phage Quasimodo (NC_054721) [156,354 nt]
Mycobacterium phage Breeniome (NC_029044) [154,434 nt]
Mycobacterium phage ArcherS7 (NC_021348) [156,558 nt]
Mycobacterium phage Shrimp (KF024734) [155,714 nt]
Mycobacterium phage Gizmo (NC_021346) [157,482 nt]
Mycobacterium phage Pleione (NC_023737) [155,586 nt]
Mycobacterium phage Ava3 (JQ911768) [154,466 nt]
Mycobacterium phage Willis (KJ595575) [155,476 nt]
Mycobacterium phage Catera (NC_008207) [153,766 nt]
Mycobacterium phage Wally (JN699625) [155,299 nt]
Mycobacterium phage Rizal (NC_011272) [153,894 nt]
Arthrobacter phage Decurro (NC_028692) [15,524 nt]
Arthrobacter phage Atraxa (NC_070767) [14,927 nt]
Rhodococcus phage RRH1 (NC_016651) [14,270 nt]
Gordonia phage EricDab (NC_070997) [16,658 nt]
Gordonia phage SallySpecial (NC_070996) [15,896 nt]
Gordonia phage Emperor (NC_070995) [16,604 nt]
Gordonia phage McGonagall (NC_030912) [17,119 nt]
Gordonia phage Coeur (NC_070994) [16,223 nt]
Gordonia phage GRU3 (NC_030935) [17,727 nt]

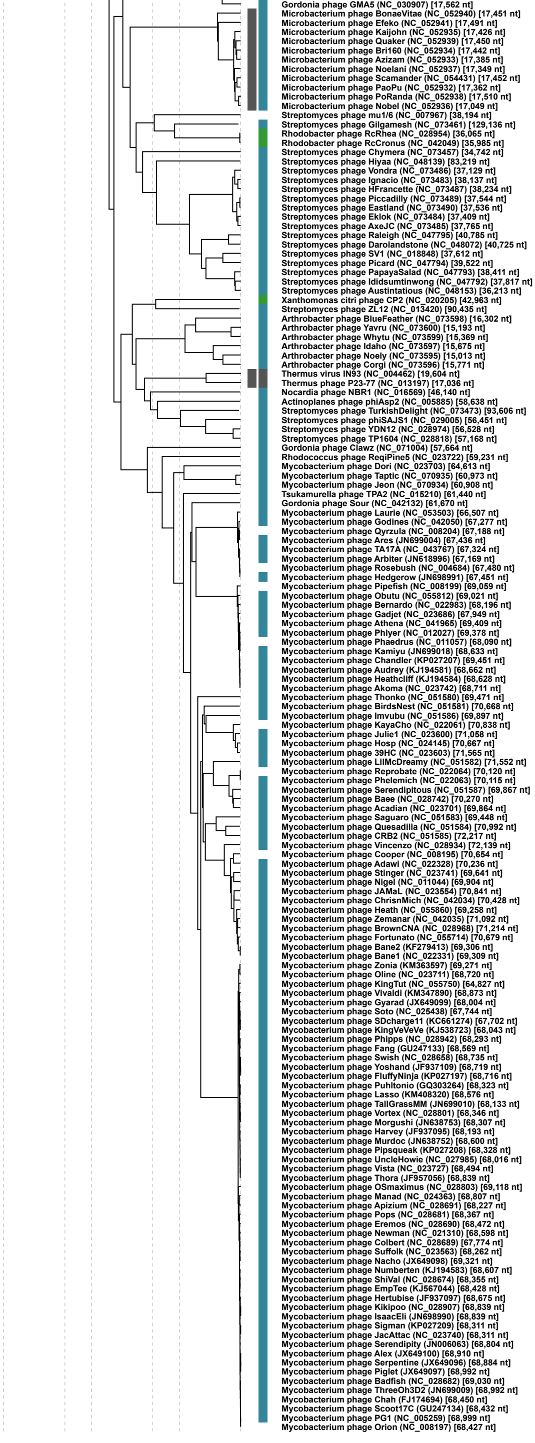

Gordonia phage GMA5 (NC_030907) [17,562 nt]
Microbacterium phage BonaeVitae (NC_052940) [17,451 nt]
Microbacterium phage Efeko (NC_052941) [17,491 nt]
Microbacterium phage Kaijohn (NC_052935) [17,426 nt]
Microbacterium phage Quaker (NC_052939) [17,450 nt]
Microbacterium phage Bri160 (NC_052934) [17,442 nt]
Microbacterium phage Azizam (NC_052933) [17,385 nt]
Microbacterium phage Noelani (NC_052937) [17,349 nt]
Microbacterium phage Scamander (NC_054431) [17,452 nt]
Microbacterium phage PaoPu (NC_052932) [17,362 nt]
Microbacterium phage PoRanda (NC_052938) [17,510 nt]
Microbacterium phage Nobel (NC_052936) [17,049 nt]
Streptomyces phage mu1/6 (NC_007967) [38,194 nt]
Streptomyces phage Gilgamesh (NC_073461) [129,136 nt]
Rhodobacter phage RcRhea (NC_028954) [36,065 nt]
Rhodobacter phage RcCronus (NC_042049) [35,985 nt]
Streptomyces phage Chymera (NC_073457) [34,742 nt]
Streptomyces phage Hiyaa (NC_048139) [83,219 nt]
Streptomyces phage Vondra (NC_073486) [37,129 nt]
Streptomyces phage Ignacio (NC_073483) [38,137 nt]
Streptomyces phage HFrancette (NC_073487) [38,234 nt]
Streptomyces phage Piccadilly (NC_073489) [37,544 nt]
Streptomyces phage Eastland (NC_073490) [37,536 nt]
Streptomyces phage Eklok (NC_073484) [37,409 nt]
Streptomyces phage AxeJC (NC_073485) [37,765 nt]
Streptomyces phage Raleigh (NC_047795) [40,785 nt]
Streptomyces phage Darolandstone (NC_048072) [40,725 nt]
Streptomyces phage SV1 (NC_018848) [37,612 nt]
Streptomyces phage Picard (NC_047794) [39,522 nt]
Streptomyces phage PapayaSalad (NC_047793) [38,411 nt]
Streptomyces phage Ididsumtinwong (NC_047792) [37,817 nt]
Streptomyces phage Austintatious (NC_048153) [36,213 nt]
Xanthomonas citri phage CP2 (NC_020205) [42,963 nt]
Streptomyces phage ZL12 (NC_013420) [90,435 nt]
Arthrobacter phage BlueFeather (NC_073598) [16,302 nt]
Arthrobacter phage Yavru (NC_073600) [15,193 nt]
Arthrobacter phage Whytu (NC_073599) [15,369 nt]
Arthrobacter phage Idaho (NC_073597) [15,675 nt]
Arthrobacter phage Noely (NC_073595) [15,013 nt]
Arthrobacter phage Corgi (NC_073596) [15,771 nt]
Thermus virus IN93 (NC_004462) [19,604 nt]
Thermus phage P23-77 (NC_013197) [17,036 nt]
Nocardia phage NBR1 (NC_016569) [46,140 nt]
Actinoplanes phage phiAsp2 (NC_005885) [58,638 nt]
Streptomyces phage TurkishDelight (NC_073473) [93,606 nt]
Streptomyces phage phiSAJS1 (NC_029005) [56,451 nt]
Streptomyces phage YDN12 (NC_028974) [56,528 nt]
Streptomyces phage TP1604 (NC_028818) [57,168 nt]
Gordonia phage Clawz (NC_071004) [57,664 nt]
Rhodococcus phage ReqiPine5 (NC_023722) [59,231 nt]
Mycobacterium phage Dori (NC_023703) [64,613 nt]
Mycobacterium phage Taptic (NC_070935) [60,973 nt]
Mycobacterium phage Jeon (NC_070934) [60,908 nt]
Tsukamurella phage TPA2 (NC_015210) [61,440 nt]
Gordonia phage Sour (NC_042132) [61,670 nt]
Mycobacterium phage Laurie (NC_053503) [66,507 nt]
Mycobacterium phage Godines (NC_042050) [67,277 nt]
Mycobacterium phage Qyrzula (NC_008204) [67,188 nt]
Mycobacterium phage Ares (JN699004) [67,436 nt]
Mycobacterium phage TA17A (NC_043767) [67,324 nt]
Mycobacterium phage Arbiter (JN618996) [67,169 nt]
Mycobacterium phage Rosebush (NC_004684) [67,480 nt]
Mycobacterium phage Hedgerow (JN698991) [67,451 nt]
Mycobacterium phage Pipefish (NC_008199) [69,059 nt]
Mycobacterium phage Obutu (NC_055812) [69,021 nt]
Mycobacterium phage Bernardo (NC_022983) [68,196 nt]
Mycobacterium phage Gadjet (NC_023686) [67,949 nt]
Mycobacterium phage Athena (NC_041965) [69,409 nt]
Mycobacterium phage Phlyer (NC_012027) [69,378 nt]
Mycobacterium phage Phaedrus (NC_011057) [68,090 nt]
Mycobacterium phage Kamiyu (JN699018) [68,633 nt]
Mycobacterium phage Chandler (KP027207) [69,451 nt]
Mycobacterium phage Audrey (KJ194581) [68,662 nt]
Mycobacterium phage Heathcliff (KJ194584) [68,628 nt]
Mycobacterium phage Akoma (NC_023742) [68,711 nt]
Mycobacterium phage Thonko (NC_051580) [69,471 nt]
Mycobacterium phage BirdsNest (NC_051581) [70,668 nt]
Mycobacterium phage Imvubu (NC_051586) [69,897 nt]
Mycobacterium phage KayaCho (NC_022061) [70,838 nt]
Mycobacterium phage Julie1 (NC_023600) [71,058 nt]
Mycobacterium phage Hosp (NC_024145) [70,667 nt]
Mycobacterium phage 39HC (NC_023603) [71,565 nt]
Mycobacterium phage LilMcDreamy (NC_051582) [71,552 nt]
Mycobacterium phage Reprobate (NC_022064) [70,120 nt]
Mycobacterium phage Phelemich (NC_022063) [70,115 nt]
Mycobacterium phage Serendipitous (NC_051587) [69,867 nt]
Mycobacterium phage Baee (NC_028742) [70,270 nt]
Mycobacterium phage Acadian (NC_023701) [69,864 nt]
Mycobacterium phage Saguaro (NC_051583) [69,448 nt]
Mycobacterium phage Quesadilla (NC_051584) [70,992 nt]
Mycobacterium phage CRB2 (NC_051585) [72,217 nt]
Mycobacterium phage Vincenzo (NC_028934) [72,139 nt]
Mycobacterium phage Cooper (NC_008195) [70,654 nt]
Mycobacterium phage Adawi (NC_022328) [70,236 nt]
Mycobacterium phage Stinger (NC_023741) [69,641 nt]
Mycobacterium phage Nigel (NC_011044) [69,904 nt]
Mycobacterium phage JAMaL (NC_023554) [70,841 nt]
Mycobacterium phage ChrisnMich (NC_042034) [70,428 nt]
Mycobacterium phage Heath (NC_055860) [69,258 nt]
Mycobacterium phage Zemanar (NC_042035) [71,092 nt]
Mycobacterium phage BrownCNA (NC_028968) [71,214 nt]
Mycobacterium phage Fortunato (NC_055714) [70,679 nt]
Mycobacterium phage Bane2 (KF279413) [69,306 nt]
Mycobacterium phage Bane1 (NC_022331) [69,309 nt]
Mycobacterium phage Zonia (KM363597) [69,271 nt]
Mycobacterium phage Oline (NC_023711) [68,720 nt]
Mycobacterium phage KingTut (NC_055750) [64,827 nt]
Mycobacterium phage Vivaldi (KM347890) [68,873 nt]
Mycobacterium phage Gyarad (JX649099) [68,004 nt]
Mycobacterium phage Soto (NC_025438) [67,744 nt]
Mycobacterium phage SDcharge11 (KC661274) [67,702 nt]
Mycobacterium phage KingVeVeVe (KJ538723) [68,043 nt]
Mycobacterium phage Phipps (NC_028942) [68,293 nt]
Mycobacterium phage Fang (GU247133) [68,569 nt]
Mycobacterium phage Swish (NC_028658) [68,735 nt]
Mycobacterium phage Yoshand (JF937109) [68,719 nt]
Mycobacterium phage FluffyNinja (KP027197) [68,716 nt]
Mycobacterium phage Puhltonio (GQ303264) [68,323 nt]
Mycobacterium phage Lasso (KM408320) [68,576 nt]
Mycobacterium phage TallGrassMM (JN699010) [68,133 nt]
Mycobacterium phage Vortex (NC_028801) [68,346 nt]
Mycobacterium phage Morgushi (JN638753) [68,307 nt]
Mycobacterium phage Harvey (JF937095) [68,193 nt]
Mycobacterium phage Murdoc (JN638752) [68,600 nt]
Mycobacterium phage Pipsqueak (KP027208) [68,328 nt]
Mycobacterium phage UncleHowie (NC_027985) [68,016 nt]
Mycobacterium phage Vista (NC_023727) [68,494 nt]
Mycobacterium phage Thora (JF957056) [68,839 nt]
Mycobacterium phage OSmaximus (NC_028803) [69,118 nt]
Mycobacterium phage Manad (NC_024363) [68,807 nt]
Mycobacterium phage Apizium (NC_028691) [68,227 nt]
Mycobacterium phage Pops (NC_028681) [68,367 nt]
Mycobacterium phage Eremos (NC_028690) [68,472 nt]
Mycobacterium phage Newman (NC_021310) [68,598 nt]
Mycobacterium phage Colbert (NC_028689) [67,774 nt]
Mycobacterium phage Suffolk (NC_023563) [68,262 nt]
Mycobacterium phage Nacho (JX649098) [69,321 nt]
Mycobacterium phage Numberten (KJ194583) [68,607 nt]
Mycobacterium phage ShiVal (NC_028674) [68,355 nt]
Mycobacterium phage EmpTee (KJ567044) [68,428 nt]
Mycobacterium phage Hertubise (JF937097) [68,675 nt]
Mycobacterium phage Kikipoo (NC_028907) [68,839 nt]
Mycobacterium phage IsaacEli (JN698990) [68,839 nt]
Mycobacterium phage Sigman (KP027209) [68,311 nt]
Mycobacterium phage JacAttac (NC_023740) [68,311 nt]
Mycobacterium phage Serendipity (JN006063) [68,804 nt]
Mycobacterium phage Alex (JX649100) [68,910 nt]
Mycobacterium phage Serpentine (JX649096) [68,884 nt]
Mycobacterium phage Piglet (JX649097) [68,992 nt]
Mycobacterium phage Badfish (NC_028682) [69,030 nt]
Mycobacterium phage ThreeOh3D2 (JN699009) [68,992 nt]
Mycobacterium phage Chah (FJ174694) [68,450 nt]
Mycobacterium phage Scoot17C (GU247134) [68,432 nt]
Mycobacterium phage PG1 (NC_005259) [68,999 nt]
Mycobacterium phage Orion (NC_008197) [68,427 nt]
