# Supplementary material for: The Citizen Phage Library: Rapid Isolation of Phages for the Treatment of Antibiotic Resistant Infections in the UK
Source: Microorganisms. 2024 Jan 25;12(2):253. doi: 10.3390/microorganisms12020253 (PMC10893117; doi:10.3390/microorganisms12020253)
Supplement: Supplementary file 1 [file microorganisms-12-00253-s001.zip › microorganisms-2789885 Supplementary Material_01.pdf]

The Citizen Phage Library: rapid isolation of phages for the treatment of antibiotic resistant infections in the UK

Supplementary Material

Methods

Table S1. Antibiotic susceptibility profiles for *K. pneumoniae*, *E. cloacae* and *E. coli* strains (susceptibility information provided by the clinical microbiology laboratory).

	Carba-5-IMP Test	Carba-5-KPC Test	Carba-5-NDM Test	Carba-5-OXA-48-Test	Carba-5-VIM Test	Cefiderocol	Ceftazidime-Avibactam	Ceftolozane-Tazobactam	Chloramphenicol	Ciprofloxacin	Co-Trimoxazole	Gentamicin	Amikacin
Klebsiella pneumoniae (BPRG 1484)	Pos	Neg	Neg	Neg	Neg	S	R	R	S	R	R	S	S
Enterobacter cloacae (BPRG 1482)	Pos	Neg	Neg	Neg	Neg	S	R	R	S	S	R	R	S
Escherichia coli (BPRG 1486)	Pos	Neg	Neg	Neg	Neg	S	R	R	S	R	R	S	S

Axenic phage purification

For axenic phage purification two methods were used. For the first approach, phage suspensions in SM buffer were serially diluted (1:10) in sterile PBS in a microtitre plate and 5 µL of each dilution was spotted onto inoculated top agar overlays. Following overnight incubation at 37 °C, a well isolated plaque was picked from the highest dilution yielding plaques. The core was placed in 100 µL of SM buffer and the process was repeated a further two times. At the end of three rounds of purification, the phage suspension in SM buffer was considered to be axenic. For the second approach, the phage suspension was serially diluted (1:10) in sterile PBS in a microtitre plate as before. Each dilution was then spotted (5 µL) onto inoculated top agar and incubated overnight at 37 °C. The dilution plate was stored at 4 °C. The highest dilution giving plaques (2-10 individual plaques within the 5 µL spot) was then noted. Using the original microtitre dilution plate, 50-100 µL of the selected dilution was added to 3 mL of molten top agar along with 1 mL of mid-logarithimic

culture and poured over the surface of a 9 cm diameter bottom agar plate. After overnight incubation, well isolated plaques were usually obtained with good clearance around each plaque. A single well isolated plaque was selected and transferred to SM buffer. At this point the phage was considered axenic.

In vivo Galleria mellonella infection assay

The efficacy of *K. pneumoniae* phage cocktails was assessed using an *in vivo G. mellonella* model described by Champion *et al.* (40). The bacterial inoculum was prepared as follows. A mid-logarithmic culture of *K. pneumoniae* BPRG 1484 grown in sLB was centrifuged at 5,000 $\times$ *g* for 10 min and the resulting pellet resuspended in sterile saline to give an OD_{600} of 1 ±0.05. Viable counts were carried out on the bacterial suspension according to the method of Miles and Misra (57). A *K. pneumoniae* suspension with an OD_{600} = 1 was expected to contain approximately 5×10^8 CFU/mL. Phage seed lots were first checked for sterility by spotting a 5 µL drop of the preparation onto LB agar. Plates were checked for microbial growth following overnight incubation at 37 °C. PFU/mL counts were carried out to determine the titre of the phage seed lots as described previously. Dilutions of the phage seed lots were prepared in sterile saline as required. Research-grade *G. mellonella* larvae in their final-instar stage were obtained from the *Galleria mellonella* Research Centre (University of Exeter, UK). Larvae were stored in the dark at 15 °C and used within the week of arrival. Larvae were divided into treatment groups and any that were discoloured or appeared to be in poor health were discarded. A 250 µL Hamilton syringe (Merck, Darmstadt, Germany) was used for inoculations. The needle was prepared before and between treatments by washing twice in 70% ethanol and twice in 1$\times$ PBS. To check needle sterility before and after injections, PBS washes were spotted onto LB agar, placed at 37 °C and checked for microbial growth following overnight incubation. Separate needles were used for bacterial and phage inoculums. Larvae were injected with 10 µL of inoculum in the first left proleg. Where two injections were necessary, the second injection was delivered into the first right proleg after a 30 min rest period. Injected larvae were placed on filter paper in petri dishes and any larvae showing signs of injury or loss of haemolymph from the injection site were replaced. No-stab controls were observed alongside each experiment. Once injections were complete, the larvae were transferred to 3D printed plastic trays with wells to separate and contain individual larvae (Figure S1). Trays were covered with fitted lids and incubated at 37 °C.

Table S2. Treatment groups for the experiment to determine effect of phage cocktails on the melanisation of *K. pneumoniae* infected *G. mellonella* larvae (n = 10). Experiment carried out once only. The dose for the *K. pneumoniae* inoculum was 4.0×10^6 CFU in saline delivered in a 10 µL volume. The dose for the phage cocktails was 2×10^7 PFU in saline delivered in a 10 µL volume (with 1×10^7 PFU for each phage in the two-phage cocktails and 5×10^6 PFU for each phage in the four-phage cocktail). Larvae receiving no injections were kept in a separate petri-dish.

Treatment group (n = 10)	Injection 1 (10 µL)	Injection 2 (10 µL)
1 (uninfected)	saline	saline
2 (uninfected)	saline	cocktail 1 (CPL00362 and CPL00368)
3 (uninfected)	saline	cocktail 2 (CPL00369 and CPL00379)
4 (uninfected)	saline	cocktail 3 (CPL00362, CPL00368, CPL00369 and CPL00379)
5 (infected)	*K. pneumoniae* BPRG 1484	saline
6 (infected)	*K. pneumoniae* BPRG 1484	cocktail 1 (CPL00362 and CPL00368)
7 (infected)	*K. pneumoniae* BPRG 1484	cocktail 2 (CPL00369 and CPL00379)
8 (infected)	*K. pneumoniae* BPRG 1484	cocktail 3 (CPL00362, CPL00368, CPL00369 and CPL00379)

Table S3. Plate layouts for the 8 treatment groups for the experiment to determine effect of phage cocktails on the survival and melanisation of *K. pneumoniae* BPRG 1484 infected *G. mellonella* larvae (n = 10).

a) Plate 1 layout

J	1	1	1	1	1
I	5	5	5	5	5
H	6	6	6	6	6
G	7	7	7	7	7
F	8	8	8	8	8
E	1	1	1	1	1
D	5	5	5	5	5
C	6	6	6	6	6
B	7	7	7	7	7
A	8	8	8	8	8

b) Plate 2 layout

J					
I	3	3	3	3	3
H	4	4	4	4	4
G	5	5	5	5	5
F					
E					
D	3	3	3	3	3
C	4	4	4	4	4
B	5	5	5	5	5
A					

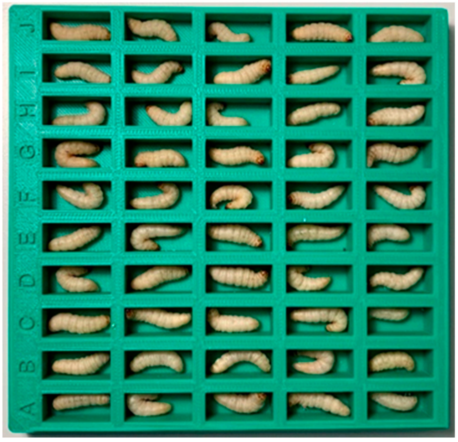

Figure S1. 3D printed plastic trays with wells to separate and contain individual *G. mellonella* larvae. Plastic lids were securely fitted to the tray to prevent escape during the assay.

Commented [M1]: Please merge subtables a,b into one table if possible.

Commented [JF2R1]: No, these tables represent 96 well plates and it would not make sense to merge them.

Results

Table S4a. Characteristics of the 20 phages active against the *K. pneumoniae* BPRG 1484 strain and their citizen scientist given names.

Phage	Isolating host	Given name	🟠 Novel species<br>🔵 Novel strain	ANI‡ (%)	Closest known phage (accession number)	Family	Genus	Genome length (bp)	Accession number
CPL00345	*K. pneumoniae* DFI 20.19	GlastoCabaret	🟠	91.7	Klebsiella phage vB_Kpn_B01 (MT380195)	*Demerecviridae*	*Sugarlandvirus*	110,382	OR896840
CPL00361	*K. pneumoniae* BPRG 1484	Identical to CPL00362		94.1	Klebsiella phage NL_ZS_3 (MT813142)	*Autographiviridae*	*Teetrevirus*	38,263	OR896841
CPL00362	*K. pneumoniae* BPRG 1484	Bobalons	🟠	94.1	Klebsiella phage NL_ZS_3 (MT813142)	*Autographiviridae*	*Teetrevirus*	38,265	OR896842
CPL00363	*K. pneumoniae* BPRG 1484		🔵	95.2	Klebsiella phage Sugarland (NC_042093)	*Demerecviridae*	*Sugarlandvirus*	110,303*	OR896843
CPL00364	*K. pneumoniae* BPRG 1484	PoeticCupcake	🟠	94.7	Klebsiella phage KPP-5 (MW600722)	*Autographiviridae*	*Teetrevirus*	38,959	OR896844
CPL00365	*K. pneumoniae* BPRG 1484	SmellyBerry	🟠	91.0	Klebsiella phage PhiKpNIH-10 (MN395285)	*Drexlerviridae*	*Webervirus*	49,698	OR896845
CPL00366	*K. pneumoniae* BPRG 1484	RareGolfball	🟠	86.0	Klebsiella phage KOX1 (NC_047825)	*Drexlerviridae*	*Webervirus*	50,866*	OR896846
CPL00367	*K. pneumoniae* BPRG 1484	HelplessSquare	🟠	92.4	Klebsiella phage cp25 (OX335410)	*Drexlerviridae*	*Webervirus*	50,894	OR896847
CPL00368	*K. pneumoniae* BPRG 1484	DevonBitter	🟠	93.6	Klebsiella phage Sugarland (NC_042093)	*Demerecviridae*	*Sugarlandvirus*	109,055*	OR896848
CPL00369	*K. pneumoniae* BPRG 1484		🔵	95.5	Klebsiella phage pK8 (OL702938)	*Drexlerviridae*	*Webervirus*	49,594*	OR896849
CPL00370	*K. pneumoniae* BPRG 1484	ViciousJeremy	🟠	94.5	Klebsiella phage KPP-5 (MW600722)	*Autographiviridae*	*Teetrevirus*	38,798	OR896850
CPL00371	*K. pneumoniae* BPRG 1484	MegaDucksbill	🟠	93.7	Klebsiella phage KPP-5 (MW600722)	*Autographiviridae*	*Teetrevirus*	38,471	OR896851
CPL00372	*K. pneumoniae* BPRG 1484	Bumbleweed	🟠	91.8	Klebsiella phage KPP-5 (MW600722)	*Autographiviridae*	*Teetrevirus*	38,833	OR896852
CPL00373	*K. pneumoniae* BPRG 1484		🔵	96.6	Klebsiella phage mtp25 (OX335391)	*Drexlerviridae*	*Webervirus*	48,297*	OR896853
CPL00374	*K. pneumoniae* BPRG 1484	Keithsmous	🟠	91.1	Klebsiella phage KPP-5 (MW600722)	*Autographiviridae*	*Teetrevirus*	39,057	OR896854
CPL00375	*K. pneumoniae* BPRG 1484	Keithstache	🟠	94.2	Klebsiella phage NL_ZS_3 (MT813142)	*Autographiviridae*	*Teetrevirus*	39,044	OR896855
CPL00376	*K. pneumoniae* BPRG 1484	SlimeyKevin	🟠	90.3	Klebsiella phage KpKT21phi1 (MK278861)	*Drexlerviridae*	*Webervirus*	49,643*	OR896856
CPL00377	*K. pneumoniae* BPRG 1484	MagicalPorter	🟠	84.9	Klebsiella phage B1 (MW672037)	*Drexlerviridae*	*Webervirus*	49,849	OR896857
CPL00378	*K. pneumoniae* BPRG 1484	AloofButler	🟠	90.4	Klebsiella phage cp25 (OX335410)	*Drexlerviridae*	*Webervirus*	51,584	OR896858
CPL00379	*K. pneumoniae* BPRG 1484	StarXobjector	🟠	88.7	Klebsiella phage mtp25 (OX335391)	*Drexlerviridae*	*Webervirus*	49,862*	OR896859

‡ ANI – average nucleotide identity (%) to nearest neighbour, calculated using VIRIDIC
*denotes original contig length as redundant termini were not found with apc

Commented [M3]: Please merge subtables a,b into one table if possible.

Commented [JF4R3]: No, these tables fit nicely onto one page each. I would prefer not to merge them.

Table S4b. Characteristics of the 21 phages active against the *E. coli* BPRG 1486 strain and their citizen scientist given names.

Phage	Isolating host	Given name	● Novel species	● Novel strain	ANI‡ (%)	Closest known phage (accession number)	Family	Genus	Genome length (bp)	Accession number
CPL00121	*E. coli* BW25113			●	95.2	Escherichia phage tunus (NC_049816)	*Drexlerviridae*	*Warwickvirus*	51,258	OR896819
CPL00124	*E. coli* BW25113	Stokescottia	●		91.2	Salmonella phage L6jm (NC_048852)	*Demerecviridae*	*Tequintavirus*	109,873	OR896820
CPL00134L	*E. coli* BW25113	Phagiculus	●		47.0	Escherichia phage vB_EcoS_MM0 (MK373793)	*Drexlerviridae*	*Rogunavirinae* (subfamily)	46,458	OR896821
CPL00136	*E. coli* BW25113			●	96.5	Salmonella phage vB_SenS-3 (MT004791)	*Demerecviridae*	*Epseptimavirus*	111,837	OR896822
CPL00144L	*E. coli* BW25113	NorthRox	●		93.8	Escherichia phage vB_Eco_Sip (OU734268)	*Drexlerviridae*	*Warwickvirus*	51,138	OR896823
CPL00146S	*E. coli* BW25113	SmurfNell	●		91.0	Escherichia phage SuperGirl (MZ501105)	*Demerecviridae*	*Epseptimavirus*	111,148	OR896824
CPL00151	*E. coli* BW25113			●	96.8	Escherichia phage W115 (ON286974)	*Straboviridae*	*Krischvirus*	164,032	OR896825
CPL00152	*E. coli* BW25113	Aragogtheria	●		87.7	Salmonella phage ende (MT074454)	*Demerecviridae*	*Epseptimavirus*	111,070*	OR896826
CPL00159	*E. coli* BW25113	MikeNSara	●		94.5	Escherichia phage atuna (MN850620)	*Drexlerviridae*	*Warwickvirus*	50,759	OR896827
CPL00160	*E. coli* BW25113			●	97.0	Escherichia phage tuinn	*Drexlerviridae*	*Warwickvirus*	51,375	OR896828
CPL00168	*E. coli* BW25113	MatMar	●		94.6	Escherichia phage Jahat_MG145 (MK552105)	*Drexlerviridae*	*Warwickvirus*	50,761	OR896829
CPL00169 (contained 2 phages)	*E. coli* BW25113	LinBro	●		91.6	Escherichia phage momo (MN850580)	*Ounavirinae (subfamily)*	*Felixounavirus*	85,930	OR896830
				●	97.5	Escherichia phage tunus (NC_049816)	*Drexlerviridae*	*Warwickvirus*	50,979	OR912983
CPL00172	*E. coli* BW25113	BubbaBully	●		76.5	Enterobacter phage N5822 (MW032452)	*Drexlerviridae*	*Henuseptimavirus*	51,300	OR896831
CPL00187	*E. coli* BW25113			●	96.5	Escherichia phage W115 (ON286974)	*Straboviridae*	*Krischvirus*	163,470	OR896832
CPL00188L	*E. coli* BW25113	WaterSpirit	●		92.5	Escherichia phage Jahat_MG145 (MK552105)	*Drexlerviridae*	*Warwickvirus*	51,324	OR896833
CPL00220	*E. coli* BW25113	Baret	●		92.8	Escherichia phage vB_Eco_mar004NP2 (LR027384)	*Demerecviridae*	*Tequintavirus*	108,072	OR896834
CPL00221	*E. coli* BW25113	RobRod40	●		69.3	Escherichia phage AugustePiccard (MZ501051)	*Drexlerviridae*	*Christensenvirus*	47,574	OR896835
CPL00224	*E. coli* BW25113	Identical to CPL00121			95.2	Escherichia phage tunus (NC_049816)	*Drexlerviridae*	*Warwickvirus*	51,258	OR896836
CPL00229	*E. coli* BW25113			●	95.0	Escherichia phage vB_Eco_mar004NP2 (LR027384)	*Demerecviridae*	*Tequintavirus*	98,746	OR896837
CPL00259L	*E. coli* BW25113	McMelon	●		89.9	Escherichia virus TH11 (MT446389)		*Dhillonvirus*	44,740	OR896838
CPL00262	*E. coli* BW25113			●	95.1	Salmonella phage S147	*Demerecviridae*	*Epseptimavirus*	38,265	OR896839

‡ ANI – average nucleotide identity (%) to nearest neighbour, calculated using VIRIDIC
*denotes original contig length as redundant termini were not found with apc

Table S5. Virulence Index (V_p) for each of the phages against the target strains *K. pneumoniae* BPRG 1484 and *E. coli* BPRG 1486. The closer the V_p is to one, the greater the virulence of the phage. Phages selected for the preparation of seed lots are highlighted.

Target strain	Phage	Virulence Index (V_p)
K. pneumoniae BPRG 1484	CPL00345	0.100826
K. pneumoniae BPRG 1484	CPL00361	0.689978
K. pneumoniae BPRG 1484	CPL00362	0.817532
K. pneumoniae BPRG 1484	CPL00363	0.982511
K. pneumoniae BPRG 1484	CPL00364	0.723433
K. pneumoniae BPRG 1484	CPL00365	0.590229
K. pneumoniae BPRG 1484	CPL00366	0.306778
K. pneumoniae BPRG 1484	CPL00367	0.323811
K. pneumoniae BPRG 1484	CPL00368	1.004923
K. pneumoniae BPRG 1484	CPL00369	0.992067
K. pneumoniae BPRG 1484	CPL00370	0.721374
K. pneumoniae BPRG 1484	CPL00371	0.736925
K. pneumoniae BPRG 1484	CPL00372	0.668646
K. pneumoniae BPRG 1484	CPL00373	0.634391
K. pneumoniae BPRG 1484	CPL00374	0.662472
K. pneumoniae BPRG 1484	CPL00375	0.79346
K. pneumoniae BPRG 1484	CPL00376	0.863438
K. pneumoniae BPRG 1484	CPL00377	0.248178
K. pneumoniae BPRG 1484	CPL00378	0.257166
K. pneumoniae BPRG 1484	CPL00379	0.912943
K. pneumoniae BPRG 1484	No phage	0

Target strain	Phage	Virulence Index (V_p)
E. coli BPRG 1486	CPL00121	0.891691
E. coli BPRG 1486	CPL00124	0.222727
E. coli BPRG 1486	CPL00134L	-0.069
E. coli BPRG 1486	CPL00136	0.257928
E. coli BPRG 1486	CPL00144L	0.32647
E. coli BPRG 1486	CPL00146S	0.99137
E. coli BPRG 1486	CPL00151	1.044243
E. coli BPRG 1486	CPL00152	0.531361
E. coli BPRG 1486	CPL00159	0.96467
E. coli BPRG 1486	CPL00160	0.355708
E. coli BPRG 1486	CPL00168	1.044042
E. coli BPRG 1486	CPL00169L	0.935219
E. coli BPRG 1486	CPL00172	0.969007
E. coli BPRG 1486	CPL00187	1.012859
E. coli BPRG 1486	CPL00188L	1.05294
E. coli BPRG 1486	CPL00220	0.950268
E. coli BPRG 1486	CPL00221	-0.09499
E. coli BPRG 1486	CPL00224	1.031495
E. coli BPRG 1486	CPL00229	0.364842
E. coli BPRG 1486	CPL00259L	0.606492
E. coli BPRG 1486	CPL00262	0.585566
E. coli BPRG 1486	T7	1.009949
E. coli BPRG 1486	No phage	0

Table S6. Titre (PFU/mL) of phage lysates at each stage of the preparation of the four *K. pneumoniae* seed lots.

Phage	Mini phage lysate	Midi phage lysate	Maxi phage lysate	Precipitated phage suspension (in 20 mL saline)	CsCl phage band before dialysis	Phage band after dialysis (final seed lots)
CPL00362	10^{10}	1.2×10^{9}	TNTC	TNTC	TNTC	2.0×10^{11}
CPL00368	1.2×10^{9}	2.4×10^{11}	5.0×10^{12}	2.8×10^{14}	3.8×10^{16}	2.0×10^{10}
CPL00369	2.6×10^{10}	2.7×10^{14}	2.5×10^{9}	Not recorded	4.9×10^{15}	5.2×10^{11}
CPL00379	4.9×10^{13}	1.2×10^{8}	TNTC	3.8×10^{11}	4.3×10^{17}	4.0×10^{11}

Too numerous to count (TNTC)

Table S7. Purity of the four *K. pneumoniae* phage seed lots as determined by short-read DNA sequencing.

Phage	Number of reads	Coverage of phage genome (x-fold)	% host reads	% unmapped reads	Evidence of prophage
CPL00362	2,895,997	10,868	1.13	0.14	No
CPL00368	2,508,024	3,341	0.12	0.18	No
CPL00369	4,407,409	10,946	15.92	0.16	No
CPL00379	2,677,102	5,799	25.3	0.61	No

Galleria mellonella infection assays

The first stage in the *G. mellonella* infection model experiments was to establish the dose of *K. pneumoniae* required to induce a measurable effect on melanisation and survival of the larvae so that any rescue effect of the phage cocktails could be observed and these results are presented in Figures S2 and S3. Challenging *G. mellonella* larvae with *K. pneumoniae* at the highest dose tested of 4.6×10^6 CFU induced melanisation of the larvae and caused larval death. After 8 h, melanisation was significantly greater in larvae receiving the highest dose (Figure S2) and after 24 h, 60% of these larvae had died (Figure S3). No melanisation occurred in the larvae receiving PBS in place of *K. pneumoniae* and all larvae in this treatment group survived the duration of the experiment (24 h post-infection).

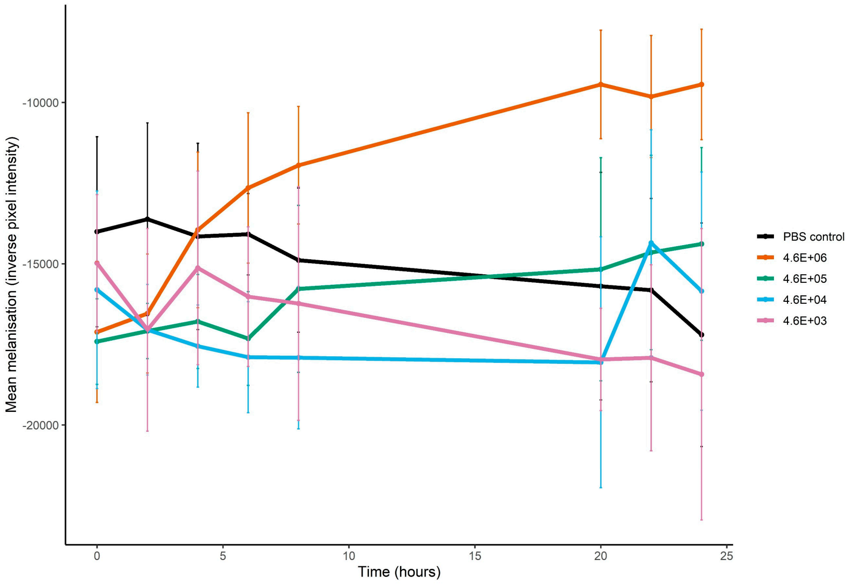

Figure S2. Effect of *K. pneumoniae* dose (CFU/mL) on melanisation of *G. mellonella* larvae. *K. pneumoniae* dose was administered in 10 µL, control larvae received 10 µL of sterile PBS.

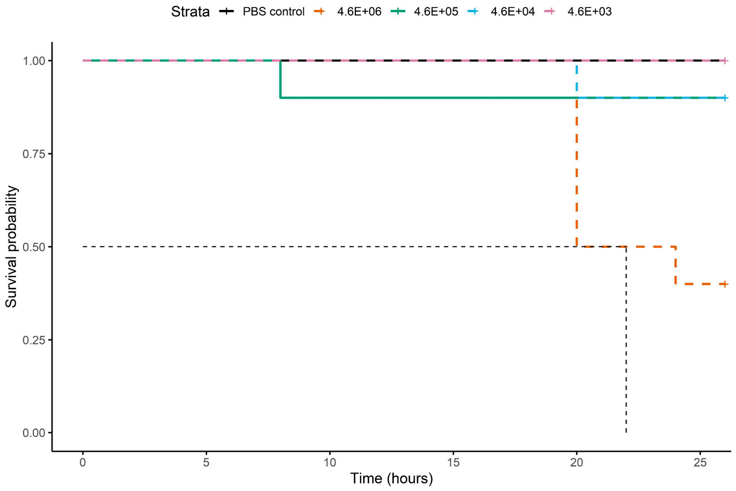

Figure S3. Effect of *K. pneumoniae* dose (CFU/mL) on survival of *G. mellonella* larvae. *K. pneumoniae* dose was administered in 10 µL, control larvae received 10 µL of sterile PBS.

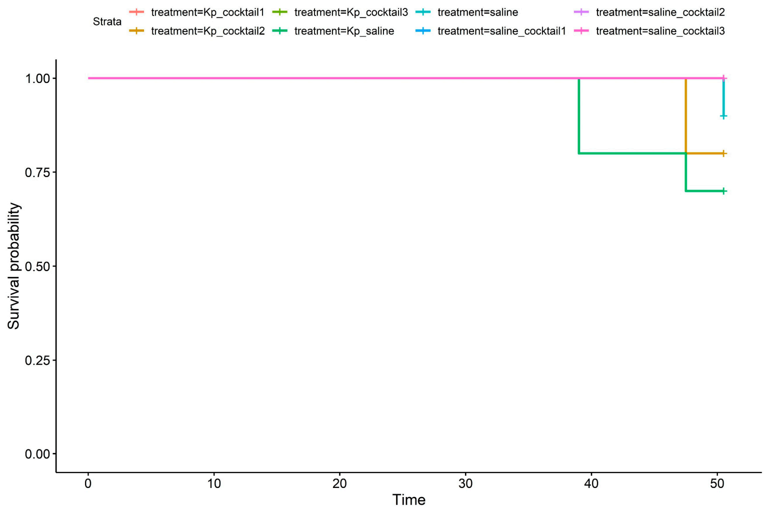

Figure S4. Effect of phage cocktails on survival of *K. pneumoniae* infected *G. mellonella* larvae. Each larvae received 2 injections: 10 µL of *K. pneumoniae* bacterial cells or 10 µL of saline and 10 µl of phage cocktail or 10 µL saline. *K. pneumoniae* dose = 4.0×10^6 CFU delivered in a 10 µL volume. Phage cocktail dose = 2×10^7 PFU in saline delivered in a 10 µL volume (with 1×10^7 PFU for each phage in the two-phage cocktails and 5×10^6 PFU for each phage in the four-phage cocktail).

Table S8. Full linear model showing the effect of treatment (saline, phage cocktail 1, phage cocktail 2, phage cocktail 3) and time on the melanisation of *K. pneumoniae* infected larvae (n = 10 per treatment group). *K. pneumoniae* infected larvae treated with saline was set as the reference group. Significance codes: 0 '***' 0.001 '**' 0.01 '*' 0.05 '.'

Commented [M5]: Revised numeration, please confirm.

Commented [JF6R5]: Accepted, thank you.

Variable	Estimate	Standard error	t value	p value
Intercept	-17121.2	1013.4	-16.9	<0.001***
Infected larvae + saline	491.3	1433.1	0.34	0.73
Infected larvae + cocktail 1	536.3	1433.1	0.37	0.70
Infected larvae + cocktail 2	119.6	1433.1	0.08	0.93
Infected larvae + cocktail 3	1382.9	1433.1	0.97	0.33
Time 14hrs	4571.1	1433.1	3.19	0.002**
Time 17hrs	4964.6	1433.1	3.46	<0.001***
Time 19hrs	5548.5	1433.1	3.87	<0.001***
Time 21hrs	5961.1	1433.1	4.16	<0.001***
Time 24hrs	6744.6	1433.1	4.71	<0.001***
**Interactions treatment:time**				
Infected larvae + saline:14hrs	-6340.1	2026.7	-3.13	0.002**
Infected larvae + cocktail 1:14hrs	-1988.2	2026.7	-0.98	0.33
Infected larvae + cocktail 2:14hrs	-2075.6	2026.7	-1.02	0.31
Infected larvae + cocktail 3:14hrs	-4751.4	2026.7	-2.34	0.02*
Infected larvae + saline:17hrs	-5.945.0	2026.7	-2.93	0..004**
Infected larvae + cocktail 1:17hrs	-2697.5	2026.7	-1.33	0.18
Infected larvae + cocktail 2:17hrs	-2409.0	2026.7	-1.19	0.24
Infected larvae + cocktail 3:17hrs	-2931.3	2026.7	-1.45	0.15
Infected larvae + saline:19hrs	-7687.1	2026.7	-3.80	<0.001***
Infected larvae + cocktail 1:19hrs	-2789.1	2026.7	-1.38	0.17
Infected larvae + cocktail 2:19hrs	-1765.4	2026.7	-0.87	0.38
Infected larvae + cocktail 3:19hrs	-4726.5	2026.7	-2.33	0.02*
Infected larvae + saline:21hrs	-6031.6	2026.7	-2.98	0.003**
Infected larvae + cocktail 1:21hrs	-2342.5	2026.7	-1.16	0.24
Infected larvae + cocktail 2:21hrs	-2007.2	2026.7	-0.99	0.32
Infected larvae + cocktail 3:21hrs	-3814.9	2026.7	-1.88	0.06
Infected larvae + saline:24hrs	-7368.7	2026.7	-3.64	<0.001***
Infected larvae + cocktail 1:24hrs	-2829.4	2026.7	-1.40	0.16
Infected larvae + cocktail 2:24hrs	-3363.4	2026.7	-1.66	0.09
Infected larvae + cocktail 3:24hrs	-5563.6	2026.7	-2.75	0.006**

(a) CPL00362

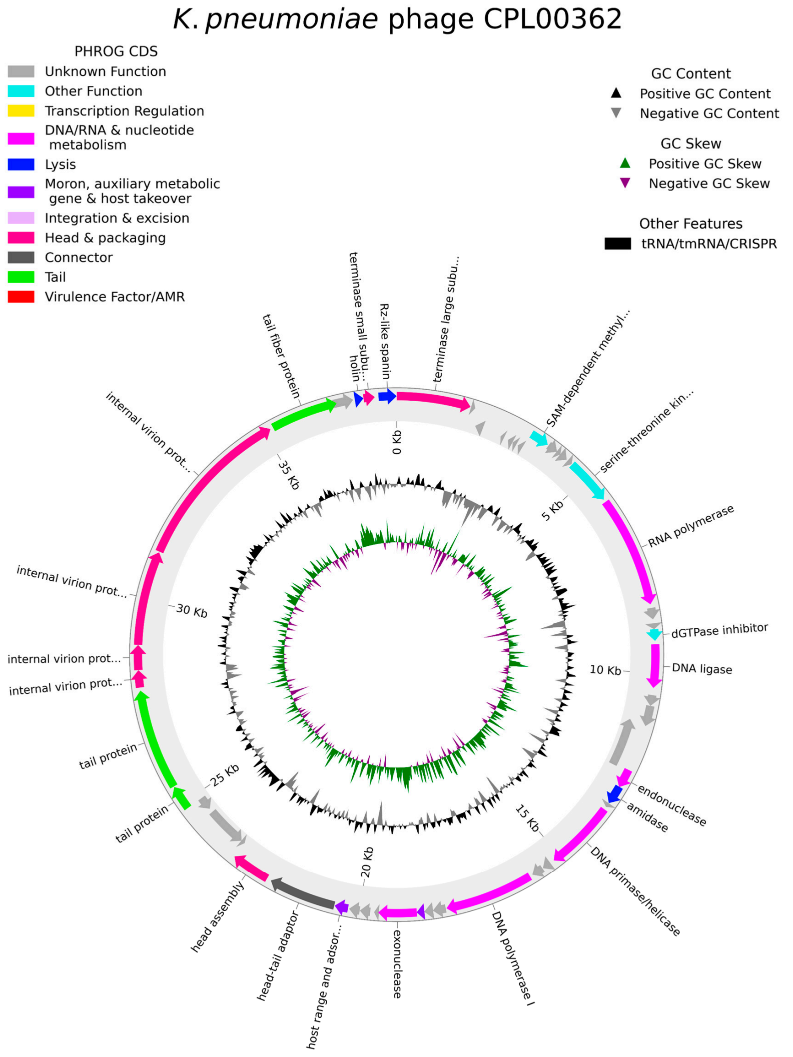
K. pneumoniae phage CPL00362
PHROG CDS
Unknown Function
Other Function
Transcription Regulation
DNA/RNA & nucleotide metabolism
Lysis
Moron, auxiliary metabolic gene & host takeover
Integration & excision
Head & packaging
Connector
Tail
Virulence Factor/AMR
GC Content
Positive GC Content
Negative GC Content
GC Skew
Positive GC Skew
Negative GC Skew
Other Features
tRNA/tmRNA/CRISPR
terminase small subu...
holin
Rz-like spanin
terminase large subu...
SAM-dependent methyl...
serine-threonine kin...
RNA polymerase
dGTPase inhibitor
DNA ligase
endonuclease
amidase
DNA primase/helicase
DNA polymerase I
exonuclease
host range and adsor...
head-tail adaptor
head assembly
tail protein
tail protein
internal virion prot...
internal virion prot...
internal virion prot...
internal virion prot...
tail fiber protein
0 Kb
5 Kb
10 Kb
15 Kb
20 Kb
25 Kb
30 Kb
35 Kb

(b) CPL00368

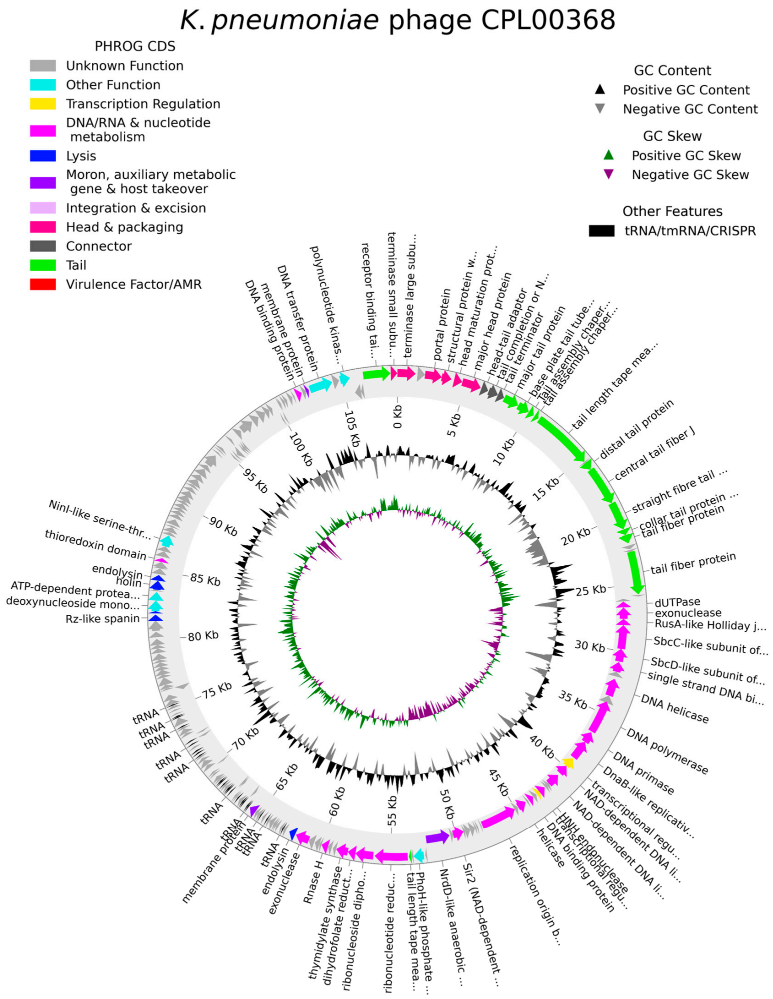
K. pneumoniae phage CPL00368
PHROG CDS
Unknown Function
Other Function
Transcription Regulation
DNA/RNA & nucleotide metabolism
Lysis
Moron, auxiliary metabolic gene & host takeover
Integration & excision
Head & packaging
Connector
Tail
Virulence Factor/AMR
GC Content
Positive GC Content
Negative GC Content
GC Skew
Positive GC Skew
Negative GC Skew
Other Features
tRNA/tmRNA/CRISPR
0 Kb
5 Kb
10 Kb
15 Kb
20 Kb
25 Kb
30 Kb
35 Kb
40 Kb
45 Kb
50 Kb
55 Kb
60 Kb
65 Kb
70 Kb
75 Kb
80 Kb
85 Kb
90 Kb
95 Kb
100 Kb
105 Kb
terminase small subu...
terminase large subu...
portal protein
structural protein w...
head maturation prot...
major head protein
head-tail adaptor
tail completion or N...
tail terminator
major tail protein
base plate tail tube...
tail assembly chaper...
tail assembly chaper...
tail length tape mea...
distal tail protein
central tail fiber)
straight fibre tail ...
collar tail protein ...
tail fiber protein
tail fiber protein
dUTPase
exonuclease
RusA-like Holliday j...
SbcC-like subunit of...
SbcD-like subunit of...
single strand DNA bi...
DNA helicase
DNA polymerase
DNA primase
DnaB-like replicativ...
transcriptional regu...
NAD-dependent DNA li...
HNH endonuclease
transcriptional regu...
DNA binding protein
helicase
replication origin b...
Sir2 (NAD-dependent ...
NrdD-like anaerobic ...
PhoH-like phosphate ...
tail length tape mea...
ribonucleotide reduc...
ribonucleoside dipho...
dihydrofolate reduct...
thymidylate synthase
Rnase H
exonuclease
endolysin
tRNA
membrane protein
tRNA
tRNA
tRNA
tRNA
tRNA
tRNA
tRNA
tRNA
tRNA
Rz-like spanin
deoxynucleoside mono...
ATP-dependent protea...
holin
endolysin
thioredoxin domain
NinI-like serine-thr...
DNA binding protein
membrane protein
DNA transfer protein
polynucleotide kinas...
receptor binding tai...

(c) CPL00369

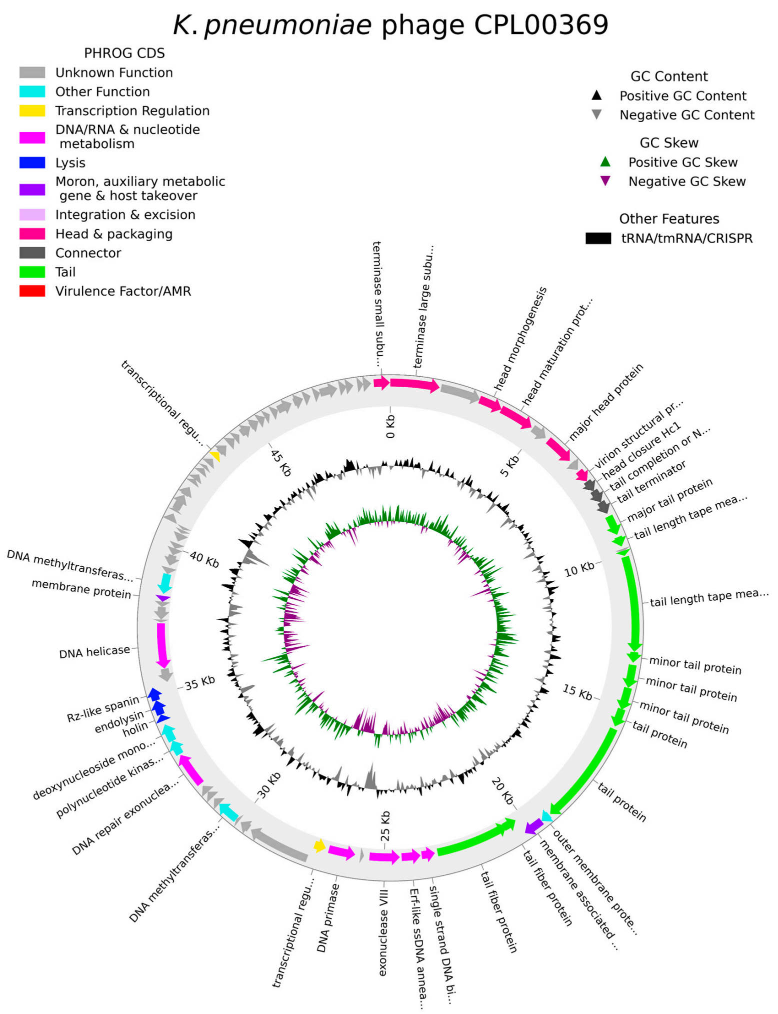

K. pneumoniae phage CPL00369
PHROG CDS
Unknown Function
Other Function
Transcription Regulation
DNA/RNA & nucleotide metabolism
Lysis
Moron, auxiliary metabolic gene & host takeover
Integration & excision
Head & packaging
Connector
Tail
Virulence Factor/AMR
GC Content
Positive GC Content
Negative GC Content
GC Skew
Positive GC Skew
Negative GC Skew
Other Features
tRNA/tmRNA/CRISPR
terminase small subu...
terminase large subu...
head morphogenesis
head maturation prot...
major head protein
virion structural pr...
head closure Hc1
tail completion or N...
tail terminator
major tail protein
tail length tape mea...
tail length tape mea...
minor tail protein
minor tail protein
minor tail protein
tail protein
tail protein
outer membrane prote...
membrane associated ...
tail fiber protein
tail fiber protein
single strand DNA bi...
Erf-like ssDNA annea...
exonuclease VIII
DNA primase
transcriptional regu...
DNA methyltransferas...
DNA repair exonuclea...
polynucleotide kinas...
deoxynucleoside mono...
holin
endolysin
Rz-like spanin
DNA helicase
membrane protein
DNA methyltransferas...
transcriptional regu...
0 Kb
5 Kb
10 Kb
15 Kb
20 Kb
25 Kb
30 Kb
35 Kb
40 Kb
45 Kb

(d) CPL00379

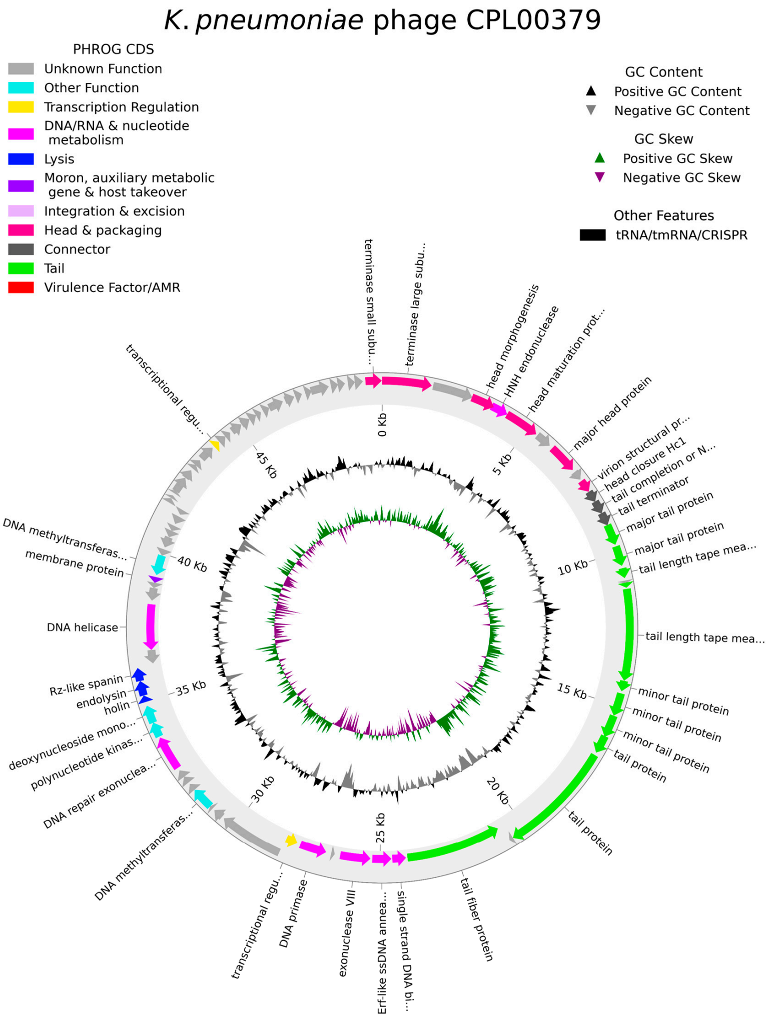

Figure S5. *K. pneumoniae* phage genomes for (**a**) CPL00362 (**b**) CPL00368 (**c**) CPL00369 and (**d**) CPL00379 annotated using pharokka (https://github.com/gbouras13/pharokka)
